# Genome-wide comparative and evolutionary analysis of Calmodulin-binding Transcription Activator (CAMTA) family in *Gossypium* species

**DOI:** 10.1038/s41598-018-23846-w

**Published:** 2018-04-03

**Authors:** Poonam Pant, Zahra Iqbal, Bhoopendra K. Pandey, Samir V. Sawant

**Affiliations:** 1 Council of Scientific and Industrial Research - National Botanical Research Institute (CSIR-NBRI), Rana Pratap Marg, Lucknow, 226001 India; 2 Academy of Scientific and Innovative Research (AcSIR), CSIR-NBRI Campus, Lucknow, India

## Abstract

The CAMTA gene family is crucial in managing both biotic and abiotic stresses in plants. Our comprehensive analysis of this gene family in cotton resulted in the identification of 6, 7 and 9 CAMTAs in three sequenced cotton species, i.e., *Gossypium arboreum*, *Gossypium raimondii*, and *Gossypium hirsutum*, respectively. All cotton CAMTAs were localized in the nucleus and possessed calmodulin-binding domain (CaMBD) as identified computationally. Phylogenetically four significant groups of cotton CAMTAs were identified out of which, Group II CAMTAs experienced higher evolutionary pressure, leading to a faster evolution in diploid cotton. The expansion of cotton CAMTAs in the genome was mainly due to segmental duplication. Purifying selection played a significant role in the evolution of cotton CAMTAs. Expression profiles of GhCAMTAs revealed that GhCAMTA2A.2 and GhCAMTA7A express profoundly in different stages of cotton fiber development. Positive correlation between expression of these two CAMTAs and fiber strength confirmed their functional relevance in fiber development. The promoter region of co-expressing genes network of GhCAMTA2A.2 and GhCAMTA7A showed a higher frequency of occurrence of CAMTA binding motifs. Our present study thus contributes to broad probing into the structure and probable function of CAMTA genes in *Gossypium* species.

## Introduction

The CAMTAs are a family of well-characterized Calmodulin (CaM) binding transcription factors that are reported to be evolutionarily conserved from plants to animals^1^. The CAMTA family was first reported in tobacco (*NtER1*) during the screening for the CaM-binding proteins^2–4^. CAMTA proteins possess multiple predicted functional domains. These domains include (i) CG-1 DNA-binding domain containing a predicted bipartite nuclear localization signal (NLS) at the N-terminus. (ii) TIG domain, implicated in nonspecific DNA contacts. (iii) Ankyrin repeats, responsible for mediating protein-protein interactions. (iv) CaMBD, and a varying number of IQ motifs (IQXXXRGXXXR), which bind with CaM in a Ca^2+^- independent manner^1–3,5,6^. In Arabidopsis, CAMTAs contain only one NLS localized in the CG-1 domain^3^. However, rice OsCBT has two NLSs each localized in the N- and C-terminal of CG-1 domain^7^. Acquiring evidence shows that these domains perform diverse functions in the regulation of gene expression^8^. CAMTA is engaged in transcriptional regulation by recognizing and binding to specific cis-elements (A/C)CGCG(C/G/T) and (A/C)CGTGT in the promoter regions of the target genes and thereby regulate the expression of the target genes^3,5,9–11^.

Plants are sessile organisms and thus vulnerable to various environmental stresses^12^. CAMTA proteins act as a pivotal component of the rapid response to an array of abiotic and biotic stresses by their efficiency to transduce calcium signals^13^. The expression of CAMTA genes in plants responds to both environmental stresses and hormonal stimuli^3,14–17^. Loss-of-function mutants of the AtCAMTA3 establishes that it is a negative regulator of plant immunity in Arabidopsis^5,11,18^. AtCAMTA1 mediates auxin response and plays a vital role in the regulation of response to drought stress^14,19^. AtCAMTA1 and AtCAMTA3 are also involved in the cold tolerance by induction of CBF genes^20,21^. Recent reports illustrate that Methylerythritol cyclo diphosphate (MEcPP) is a well established intermediary molecule of the plastidial pathway for isoprenoid production and is actively involved in inducing general stress response (GSR) by transducing AtCAMTA3^22^.

CAMTAs are well studied in some monocot and dicot plants such as Arabidopsis (6)^2^, rice (7)^7^, grape (10)^23^, soybean (15)^24^, *M. truncatula* (7)^25^, and maize (7)^26^. Cotton (*Gossypium* spp.) is the world’s most valuable fiber producing crop^27,28^, yet no substantial research reported on cotton CAMTAs. In the recent past, the genome sequences and annotation of *G. arboreum*, *G. raimondii*, and *G. hirsutum* had been completed^29–31^. This immense progress on cotton genome research provides us a broader horizon to explore CAMTA family members in allotetraploid cotton and its diploid progenitors.

Cotton is an excellent model system for plant polyploid research^32^. The genus *Gossypium* comprises 45 diploid and 5 tetraploid species^29^. About 1 to 2 million years ago (MYA) interspecific hybridization events amongst *G. arboreum* (AA genome, 2n = 2x = 26, diploid species) and *G. raimondii* (DD genome, 2n = 2 × = 26, diploid species) resulted in allotetraploid *G. hirsutum* (AADD, 2n = 4x = 52)^27,28,33^. It is one of the most widely cultivated and fiber-producing crops. Upland cotton (*G. hirsutum*) has much longer fibers than its progenitor diploid cotton^31^. To obtain an integrated image of the evolutionary characteristics and probable role of CAMTA family in cotton, we characterized this family in *G. arboreum*, *G. raimondii*, and *G. hirsutum*. We further carried out detailed genomic exploration of CAMTA proteins in *G. arboreum*, *G. raimondii*, and *G. hirsutum*. The expression profiles and co-expression network of CAMTA genes in various fiber developmental stages in allotetraploid cotton were also analyzed. This work will lead to significant refinements in understanding the functional roles and evolutionary history of CAMTA family in cotton and their potential role in cotton fiber development.

## Results

### Genome-wide identification of CAMTA genes in cotton

The HMMER search against *G. arboreum, G. raimondii*, and *G. hirsutum* genomes was performed to identify the CAMTA orthologs in the *Gossypium* species. Subsequently, all the putative CAMTA genes were confirmed through similarity and conserved domain searches against Pfam and InterproScan databases. After removal of partial sequences, a total of 22 CAMTAs, i.e., 6 GaCAMTAs (*G. arboreum*), 7 GrCAMTAs (*G. raimondii*) and 9 GhCAMTAs (*G. hirsutum*) were eventually identified (Table 1 and Supplementary Dataset S1). The length of deduced cotton CAMTA proteins varied from 907 to 1,073, 963 to 1087, and 963 to 1088 amino acids in *G. arboreum, G. raimondii*, and *G. hirsutum*, respectively. The theoretical pI ranged from 5.56 to 8.07, 5.67 to 7.9 and 5.56 to 8.72; the molecular weight varied from 102.22 kDa to 120.63 kDa, 102.6 kDa to 123.05 kDa and 106.75 kDa to 123.13 kDa and the number of introns varied from 11 to 15, 10 to 12 and 10 to 15 in *G. arboreum*, *G. ramondii*, and *G. hirsutum*, respectively. All the cotton CAMTAs identified were nuclear localized (Table 1).

**Table 1:** The CAMTA genes in *G. arboreum*, *G. raimondii*, and *G. hirsutum* and properties of the deduced proteins.

Gene Name	Gene ID	Chromosome Location^a^	Length (aa)	MW (Da)	pI	No. Of Introns	Subcellular localization
GaCAMTA2.1	Cotton_A_00802	CA_chr13(−):71972149–71984252	1073	120631.7	5.84	12	Nucleus
GaCAMTA2.2	Cotton_A_24164	CA_chr5(−):48849059–48856677	1057	119070.7	5.56	12	Nucleus
GaCAMTA4	Cotton_A_30458	CA_chr10(−):111920640–111926272	986	109945	5.68	11	Nucleus
GaCAMTA5.1	Cotton_A_34645	CA_chr9(−):96490041–96496016	907	102259.5	6.68	12	Nucleus
GaCAMTA5.2	Cotton_A_21790	CA_chr5(+):41655137–41661738	1038	116596.2	7.84	15	Nucleus
GaCAMTA7	Cotton_A_21232	CA_chr9(−):2798738–2804286	968	107062.9	8.07	11	Nucleus
GrCAMTA2.1	Gorai.013G061100.1	Chr13(−):6625467–6634301	1067	119814.7	5.74	11	Nucleus
GrCAMTA2.2	Gorai.005G220600.1	Chr05(+):60341564–60349789	1052	118318.9	5.67	12	Nucleus
GrCAMTA3.1	Gorai.008G089900.1	Chr08(−):21118330–21127475	1087	123051	6.13	11	Nucleus
GrCAMTA5.1	Gorai.006G079000.1	Chr06(+):30416003–30422953	907	102683.8	7.9	12	Nucleus
GrCAMTA5.2	Gorai.011G198600.1	Chr11(+):47994290–48001178	914	102996.6	7.58	12	Nucleus
GrCAMTA5.3	Gorai.005G065700.1	Chr05(+):7009778–7014890	910	102757.4	7.61	12	Nucleus
GrCAMTA7	Gorai.011G204700.1	Chr11(−):49581143–49586771	980	109118.1	7.35	10	Nucleus
GhCAMTA2A.1	CotAD_33349	At_chr13(−):76266490–76278551	1073	120643.6	5.8	12	Nucleus
GhCAMTA2A.2	CotAD_55078	At_chr5(−):51890245–51897861	1057	119099.8	5.56	12	Nucleus
GhCAMTA2D.1	CotAD_37712	Dt_chr5(−):10886018–10893621	1057	118913.7	5.61	12	Nucleus
GhCAMTA3A.1	CotAD_51602	At_chr12(+):5508767–5516875	1088	123132.9	6.04	12	Nucleus
GhCAMTA3D.1	CotAD_58822	Dt_chr9(+):6259590–6267679	1070	121661.3	6.17	12	Nucleus
GhCAMTA4D	CotAD_41958	Dt_chr9(+):26547082–26552392	968	108026.8	5.81	9	Nucleus
GhCAMTA5D.1	CotAD_36894	Dt_chr5(−):5701131–5710161	1016	115094.2	8.72	15	Nucleus
GhCAMTA7A	CotAD_61122	At_chr11(−):9038073–9043485	963	106755.6	6.99	10	Nucleus
GhCAMTA7D	CotAD_20100	Dt_chr11(−):56557476–56562833	976	108662.4	7.01	11	Nucleus

a Chromosomal location: ‘+’ and ‘−’ indicated the forward and reverse strand, respectively.

For the standard annotation of 22 predicted cotton CAMTAs, we followed the nomenclature system applied to Arabidopsis on the basis of highest sequence similarity with 6 AtCAMTAs (Table 1). According to the phylogenetic relationships with their orthologs in Arabidopsis, 6 GaCAMTAs were named as GaCAMTA2-GaCAMTA7 (GaCAMTA2.1, 2.2, 4, 5.1, 5.2, 7). Similarly, GrCAMTAs were classified as GrCAMTA2-GrCAMTA7 (GrCAMTA2.1, 2.2, 3.1, 5.1, 5.2, 5.3, and 7). Taking into account the genome specific location, we designated 9 GhCAMTAs as GhCAMTA2-GhCAMTA7A/D (A:A_T_ subgenome and D:D_T_ subgenome). The reciprocal blast revealed that cotton CAMTAs (GaCAMTA2.1, 2.2, GrCAMTA2.1, 2.2, GhCAMTA2A.1, 2 A.2, 2D.1 and GaCAMTA5.1, 5.2, GrCAMTA5.1, 5.2, 5.3, GhCAMTA5D.1) showed higher homology with AtCAMTA2 and AtCAMTA5 as compared to AtCAMTA1 and AtCAMTA6, respectively (Supplementary Dataset S2).

### Domain structure analysis of cotton CAMTAs

**Figure 1:**
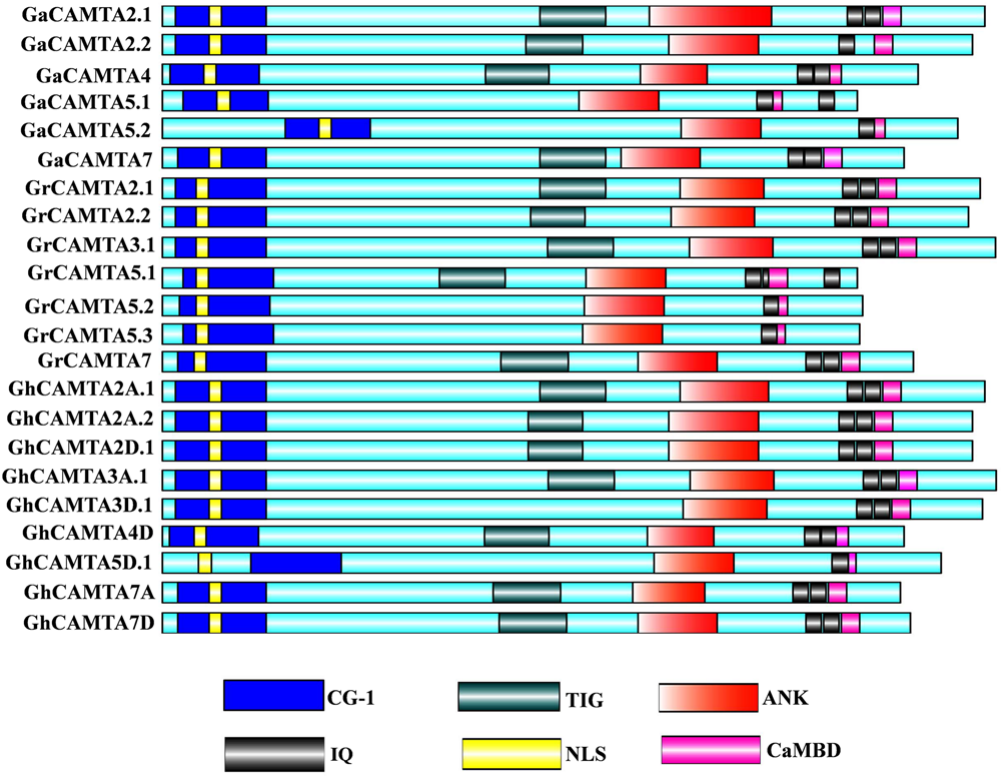
Schematic representation of functional domains of GaCAMTAs, GrCAMTAs and GhCAMTAs. Bioinformatics analysis of functional conserved domains were performed by Pfam database (http://pfam.janelia.org/). NLS and CaMBD were specifically searched in Motif scan (http://myhits.isb-sib.ch/cgi-bin/motif_scan) and Calmodulin Target Database (http://calcium.uhnres.utoronto.ca/ctdb/ctdb/) respectively. The domain structure of GaCAMTAs, GrCAMTAs and GhCAMTAs were drawn using Illustrator for Biological sequences software (http://ibs.biocuckoo.org/).

Multiple sequence alignment (MSA) of cotton CAMTAs using the ClustalX and comparison with Pfam and InterPro databases revealed that cotton CAMTAs contained typical CAMTA domains (Supplementary Fig. S1). GaCAMTA2.1, 2.2, 4 and 7 in *G. arboreum*, were predicted to contain CG-1 DNA binding domain, TIG domain, ankyrin repeats, IQ motifs, and CaMBD from the N-terminus to the C-terminus. Meanwhile, GaCAMTA5.1 and 5.2 contained all of the conserved domains excluding the TIG domain. Likewise, GrCAMTA2.1, 2.2, 3.1, 5.1, 7 and GhCAMTA2A.1, 2A.2, 2D.1, 3 A.1, 4D, 7 A, 7D contained all the conserved domain, while GrCAMTA5.2, 5.3 and GhCAMTA3D.1, 5D.1 were non-TIG CAMTAs in *G. raimondii* and *G. hirsutum*, respectively. Some plant species like *Arabidopsis thaliana, Arabidopsis lyrata, Capsella rubella* etc. also possess non-TIG CAMTAs^34^. Non-TIG CAMTAs might contribute extensively to the expansion of cotton CAMTAs. The number of IQ motifs in cotton CAMTAs varies from one to three. All cotton CAMTAs contains two IQ motifs in C-terminal. Out of 22 cotton CAMTAs, GaCAMTA2.2, GaCAMTA5.2, GrCAMTA5.2, GrCAMTA5.3, and GhCAMTA5D.1 carried one IQ motif, whereas GrCAMTA5.1 contained three (Fig. 1). This study revealed that cotton CAMTAs share the same domain organization as reported previously^1^. All the identified cotton CAMTAs contain bipartite NLS (composed of basic amino acids arginine and lysine) (Fig. 1) in the N-terminus, suggesting that this region might constitute a signal that directs cotton CAMTAs to the nucleus.

### Conservation of CaMBD in cotton CAMTAs

**Figure 2:**
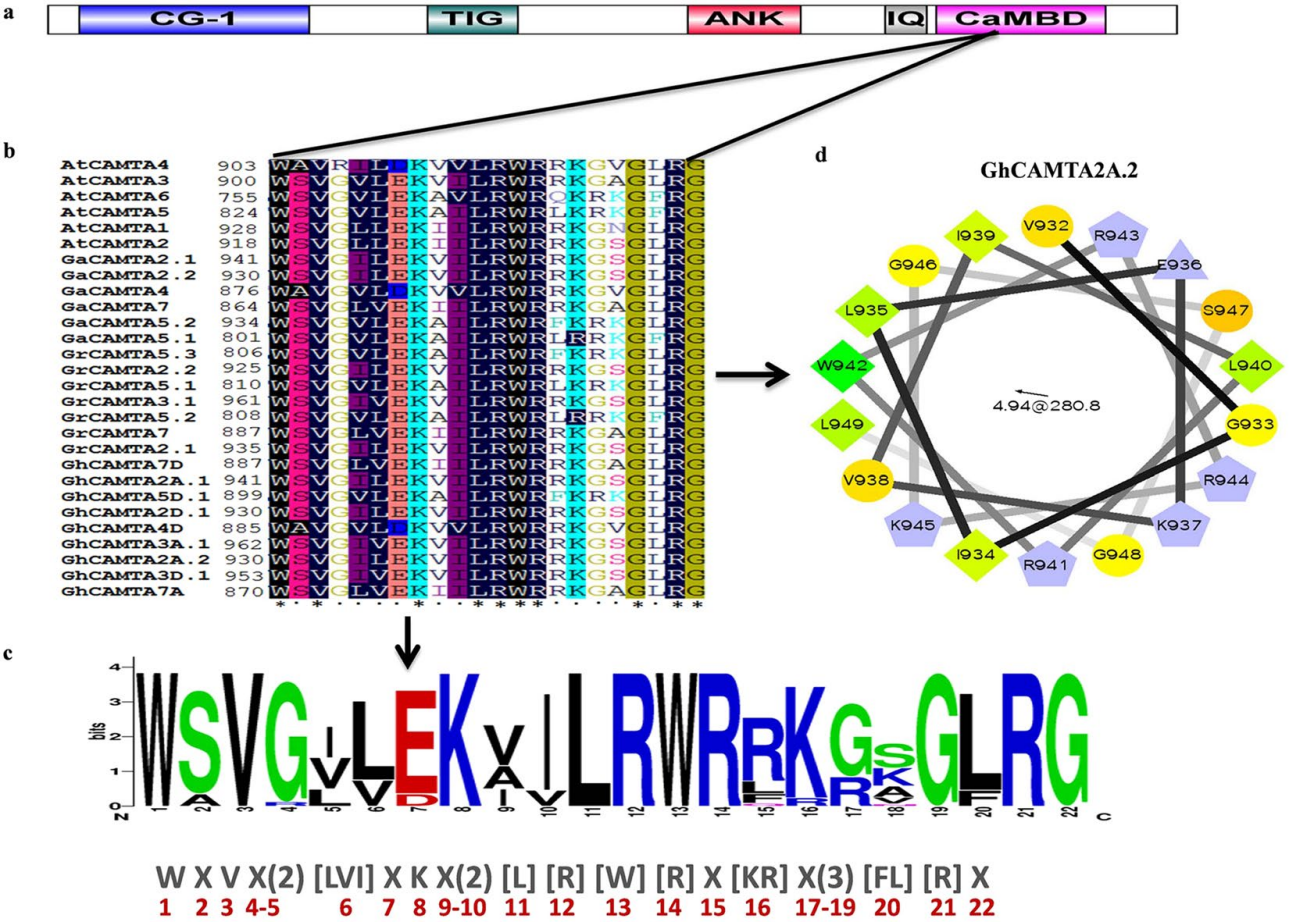
Conservation of CaMBD in all cotton CAMTA proteins. (**a**) Functionally proved motif in *Arabidopsis* CAMTAs. (**b**) Alignment of conserved CaMBD of cotton CAMTAs with 6 AtCAMTAs. (**c**) Sequence logo of the CaMBD of 22 putative cotton CAMTAs and 6 AtCAMTAs. In the square brackets “[]” are the amino acids allowed in this position of the motif; “X” represents any amino acid and the round brackets “()” indicate the number of amino acids. (**d**) Amphipathic α-helix structure in the predicted CaMBD of GhCAMTA2A.2 amino acid residues (Val^932^-Leu^949^). Circles, diamonds, triangles and pentagons represent hydrophilic residues, hydrophobic residues, potentially negatively charged and potentially positively charged residues respectively.

The CaMBD was identified in CAMTAs from all organisms except *Caenorhabditis elegans*^2^. Previous studies in Arabidopsis and tomato^2,35^ showed that CaMBD contains a functional motif (WXVX(2)LXKX(2)[LF]RWRX[KR]X(3)[FL]RX), required for CaM binding and forms amphipathic α-helix structure (Fig. 2a). The putative CAM-binding regions of cotton CAMTAs were aligned with the corresponding domains in AtCAMTAs to determine the conservation of CaMBD. We found a conserved motif sequence as (WXVX(2)[LVI]XKX(2)[L][R][W][R]X[KR]X(3)[FL][R]X). Thus, the amino acids at CaM-binding region of cotton CAMTAs have very high homology with their counterparts in AtCAMTAs. For example, GaCAMTA4 and GhCAMTA4D have almost the same amino acid sequence as AtCAMTA4 (Fig. 2b,c). We examined amphipathic α-helical properties of cotton CAMTAs; all cotton CAMTAs can form amphipathic α-helix structure. These results showed that an 18-amino acid region i.e., valine^932^ to leucine^949^ have a binding site for CaM (Fig. 2d).

### Phylogenetic relationship of cotton CAMTAs with other plant species

**Figure 3:**
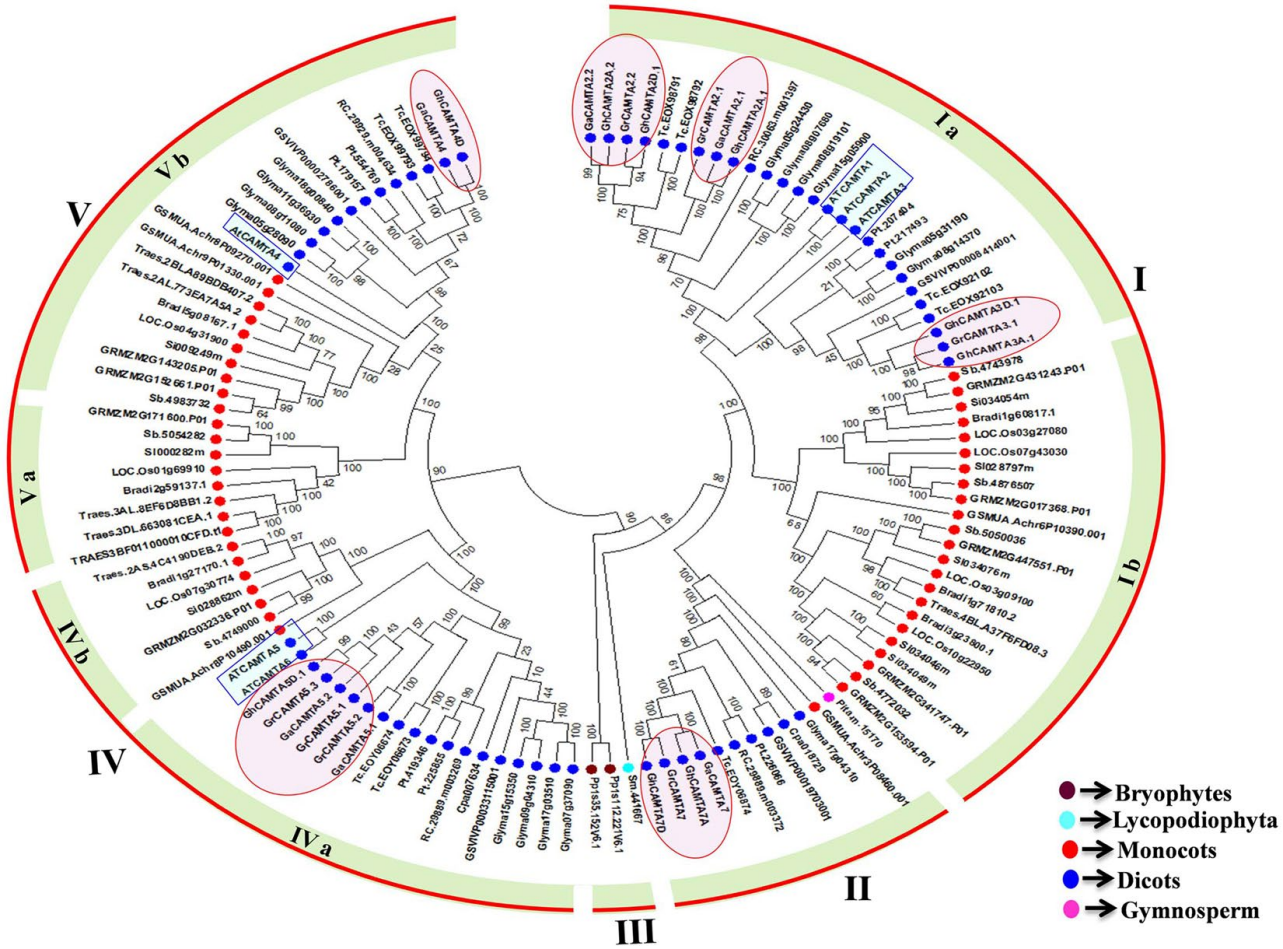
Phylogenetic relationships of cotton CAMTAs from Arabidopsis and other plant species. The unrooted phylogenetic ML tree was constructed using MEGA 5.2 software with 1000 bootstrap value. The numbers beside the branches indicate the bootstrap values that support the adjacent nodes. Different colors of dots represented the different species (Brown, Bryophytes; Sky blue, Lycopodiophytes; Red, Monocots; Blue, dicots; Pink, Gymnosperms). Cotton CAMTAs and AtCAMTAs represented by pink and sky blue color, respectively.

The evolutionary relationships among cotton CAMTAs and 17 different plant species (Supplementary Table S1) determined by an unrooted maximum likelihood tree (ML). We performed MSA of 22 identified cotton CAMTA proteins sequence along with 100 CAMTA protein sequences from different plants (bryophytes, lycopodiophytes, monocots, eudicots, and gymnosperms). Subsequently, plant CAMTAs were clustered distinctly into five major groups (I to V) while cotton CAMTAs clustered into four groups as none of its members belonged to group III (bryophytes and lycophytes). Group I, IV and V further divided into two subgroups (a and b) with robust bootstraps. Group I composed the largest clade among all groups with ten cotton CAMTA genes (Fig. 3). Majority of the CAMTA proteins from the diploid species had orthologs in the allotetraploid cotton, derived from hybridization and subsequent polyploidization of maternal A-genome and parental D-genome species^31^.

Most of the cotton CAMTAs clustered with Arabidopsis CAMTAs excluding group II CAMTAs (GaCAMTA7, GrCAMTA7, GhCAMTA7A, and GhCAMTA7D). Further, to gain a deeper understanding about group II CAMTAs, a phylogenetic tree of these CAMTAs with other eudicots (Brassicaceae, Ranunculaceae, Scrophulariaceae, Solanaceae, Vitaceae, Myrtaceae, Rutaceae, Malvaceae, Caricaceae, Rosaceae, Cucurbitaceae, Fabaceae, Salicaceae and Euphorbiaceae families) was constructed. Additionally, we also performed synteny analysis of cotton CAMTA genes with the genomes of *Theobroma cacao* (Malvaceae), *Citrus sinensis* (Rutaceae) and *Arabidopsis thaliana* (Brassicaceae). Our study revealed that the Group II CAMTAs were highly conserved between the *G. hirsutum* (At and Dt sub-genomes), *G. arboreum* (A genome), *G. raimondii* (D genome), *Theobroma cacao* and *Citrus sinensis* genomes. Thus Group II CAMTAs showed conservation with closely related species with *Gossypium*^36^ but no conserved homologs were found in Brassicaceae (Supplementary Fig. S2, Supplementary Fig. S3, and Supplementary Dataset S3). This study indicated that after diverging from a common ancestor, group II CAMTAs homologs from Brassicaceae family might have evicted.

Interestingly, CAMTAs from the bryophytes and lycophytes clustered into group III, and no member of cotton CAMTA gene family belonged to group III. Moreover, group II and six subgroups (Ia, Ib, IVa, IVb, Va, and Vb) of the groups I, IV, V, respectively were only represented by higher land plants whereas CAMTAs present in the subgroups Ib, and IVb were exclusively monocot-specific. CAMTA genes from monocotyledonous and dicotyledonous plants were present in all groups excluding group III, suggesting their evolutionary divergence before the common ancestor of monocots and dicots. The cotton CAMTA genes might share a common ancestor before the divergence of lower non-flowering (bryophytes and lycophytes) and higher flowering plants (gymnosperms and angiosperms); consequently, lineage-specific divergence and expansion events occurred in higher plants, after split from lower plants. Non-TIG CAMTAs evolved recently in flowering plant species^34^. Intriguingly, group IVa CAMTAs were mostly non-TIG types except GrCAMTA5.1 which contained all domains indicating that group IVa CAMTAs are non-TIG CAMTAs and might have specialized function in cotton. The cotton and *T. cacao* CAMTAs (both from Malvaceae) were clustered together with a high degree of reliability (>90% bootstraps) consistent with the fact that cotton and *T. cacao* CAMTAs originated from a last common ancestor 33 MYA^29^.

### Evolutionary relationship between *Gossypium* and *Theobroma cacao* CAMTAs

To explore the evolutionary and orthologous relationship between *Gossypium* and *T. cacao* CAMTAs, we investigated exon/intron pattern and surveyed *T. cacao* genome. Nine orthologous *T. cacao* CAMTA genes showed homology with their counterpart in *Gossypium*. ML tree was constructed with 1000 bootstraps. Phylogenetic analysis revealed that 31 ortholog CAMTA genes were clustered into five subfamilies (I to V). Exon/intron structure of 31 CAMTAs was comparatively analyzed showing that the CAMTA orthologs which belong to the same subfamily had similar gene structure in terms of intron number and exon length. For example, *Gossypium* and *T. cacao* CAMTAs belonging to subfamily I, contain 11 and 12 number of introns with similar exon length (Supplementary Fig. S4). This result demonstrated that both species probably diverged from a common ancestor during evolution which is consistent with the previous report^29^.

### Chromosomal distribution and duplication events of cotton CAMTA genes

**Figure 4:**
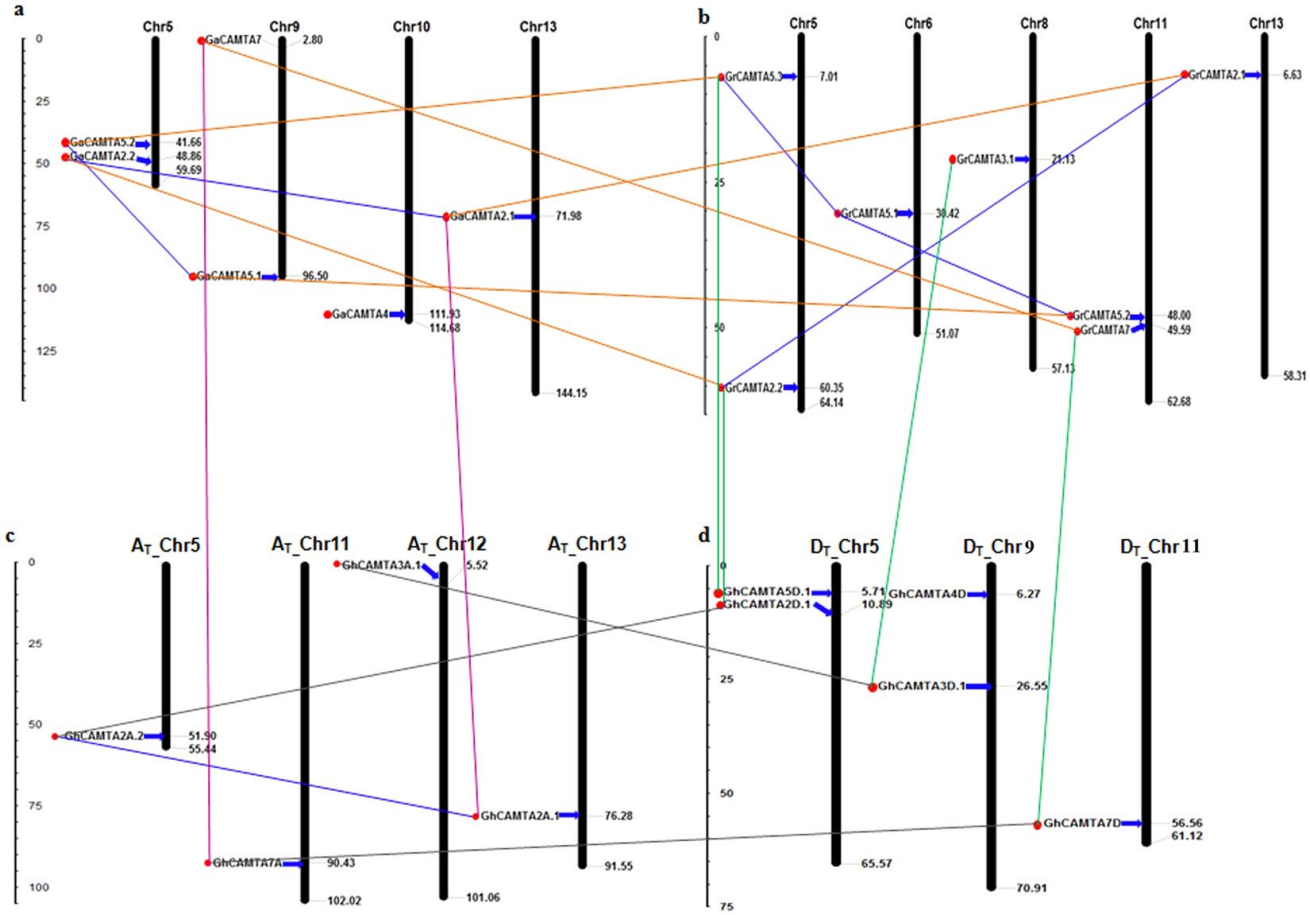
Chromosomal location and gene duplication status of CAMTA genes on *Gossypium* genomes. Physical maps show the position of CAMTA genes on A, D and AD genome separately. The paralogous CAMTA genes (segmental duplicated genes) connected with blue line. The orthologous relationship of CAMTA genes represented by various colored lines (Orange, A vs. D; Grey, A_T_ vs. D_T;_ Green, D_T_ vs. D; Pink, A_T_ vs. A). Horizontal gray line represents the location of each CAMTA genes. The chromosome number is indicated at the top of each chromosome. Upper and lower panels show the distribution of CAMTA genes in A, D and AD (A_T_ and D_T_ subgenomes) genomes, respectively. The scale is in mega bases (Mb).

BLASTN search was performed to identify the chromosomal locations of all GaCAMTA, GrCAMTA, and GhCAMTA in cotton genomes. GaCAMTA genes localized on chromosomes 5, 9, 10 and 13 (Fig. 4a), GrCAMTA genes were distributed across chromosomes 5, 6, 8, 11 and 13 (Fig. 4b). In *G. hirsutum*, a lesser number of CAMTA genes were located on A_T_ (5, 11, 12, and 13) chromosomes than D_T_ (5, 9, and 11) with 4 and 5 genes, respectively (Fig. 4c,d).

We next assessed gene duplications for the expansion of cotton CAMTAs. By high protein sequence identity and similarity, two and three pairs of putative paralogous CAMTA genes were identified in *G. arboreum* and *G. raimondii*, respectively (Fig. 4a,b), while only one pair of paralogous CAMTA gene was found in A_T_ but not in D_T_ subgenome (Fig. 4c,d). These paralogous CAMTA gene pairs were present in the same clade of the phylogenetic tree with a high degree of protein sequence identities (>75%). All of the paralogous gene pairs were positioned on different chromosomes, providing substantial evidence that expansion of cotton CAMTAs was mainly attributed to segmental duplication and not to tandem duplication. For instance, in *G. arboreum*, two segmental duplications (GaCAMTA2.1/2.2 and GaCAMTA5.1/5.2) occurred from 13.02 to 15.03 MYA, and three segmental duplications (GrCAMTA2.2/2.1, GrCAMTA5.1/5.2, and GrCAMTA5.1/5.3) were found in *G. raimondii* from 12.08 to 13.68 MYA (Table 2). Moreover, in case of *G. hirsutum*, only one segmental duplication (GhCAMTA2A.1/2A.2) in A_T_ subgenome took place 13.04 MYA. This study revealed that recent duplication (13–20 MYA) occurred in those paralogous gene pairs^29^.

**Table 2:** The Ka/Ks ratios and date of duplication for duplicate CAMTA genes in *G. arboreum, G. raimondii* and *G. hirsutum*.

Duplicated CAMTA gene 1	Duplicated CAMTA gene 2	Ka	Ks	Ka/Ks	Date(mya) T = Ks/2λ	Selective pressure	Duplicate Type
GaCAMTA2.1	GaCAMTA2.2	0.1019	0.3907	0.2607	13.02	Purifying selection	Segmental
GaCAMTA5.1	GaCAMTA5.2	0.1456	0.4511	0.3228	15.03	Purifying selection	Segmental
GrCAMTA2.2	GrCAMTA2.1	0.1018	0.4104	0.248	13.68	Purifying selection	Segmental
GrCAMTA5.1	GrCAMTA5.2	0.1233	0.3625	0.3401	12.08	Purifying selection	Segmental
GrCAMTA5.1	GrCAMTA5.3	0.1472	0.403	0.3653	13.43	Purifying selection	Segmental
GhCAMTA2A.1	GhCAMTA2A.2	0.0996	0.3914	0.2545	13.04	Purifying selection	Segmental

The Ka/Ks ratios (nonsynonymous and synonymous substitution ratios) for the duplicated cotton CAMTA gene pairs were invariably <1 (Table 2). Thus, duplicated cotton CAMTAs had undergone strong purifying selection pressure contributing to the maintenance of their function and reflecting that they had not diverged much during evolution. Since orthologs often retain equivalent functions in different species during evolution^37^, orthologous relationship among the members of CAMTA gene family was established (Fig 4a–d). Orthologs with sequence identity over 90% in both cDNA and amino acid composition were selected for further evolutionary analysis (Supplementary Dataset S4). The potential functional divergence and selection pressure of cotton CAMTAs were explored by calculating the Ka, Ks, and Ka/Ks ratios between orthologs (A vs. D, A_T_ vs. A, and D_T_ vs. D) and within homoeologs (A_T_ vs. D_T_). Surprisingly, the Ka value of cotton CAMTA7 (group II CAMTAs) orthologs (GaCAMTA7/GrCAMTA7 and GhCAMTA7A/GhCAMTA7D) was higher in inter-genomes (A vs. D and A_T_ vs. D_T_), than other ortholog CAMTA pairs, suggesting that these ortholog pairs experienced faster protein evolution. The overall Ka/Ks ratios <1, in both diploid and allotetraploid species demonstrated that CAMTA ortholog genes had experienced purifying selection pressure (Supplementary Table S2). CAMTA7 had higher Ka/Ks ratio in A vs. D, and A_T_ vs. A. Hence, CAMTA7 experienced higher evolutionary pressure in diploid cotton and might have evolved faster in A as compared to D subgenome.

### Phylogenetic tree, gene structure and protein motifs analysis of cotton CAMTAs

**Figure 5:**
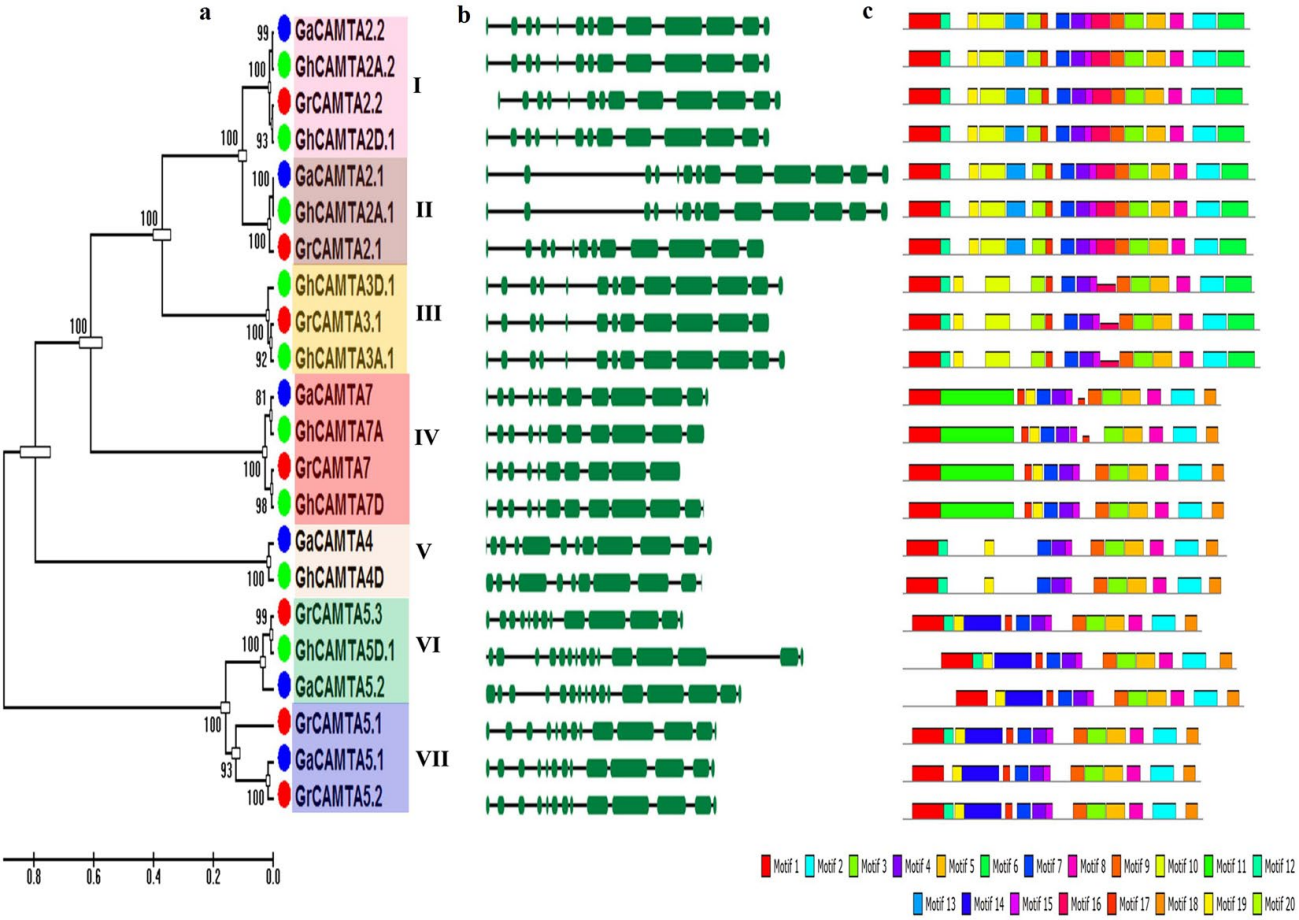
Phylogenetic tree, gene structure and conserved protein motifs analysis of CAMTAs in *G. arboreum*, *G. raimondii* and *G. hirsutum*. (**a**) Phylogenetic tree of *G. arboreum*, *G. raimondii* and *G. hirsutum* CAMTAs constructed with ML method by using 1000 bootstrap values. Different colors of dots represented the different species of *Gossypium* (Blue, *G. arboreum*; Red, *G. raimondii*; Green, *G. hirsutum*). Subfamily-I, -II, -III, -IV, -V and -VI colored in pink, brown, yellow, red, orange, green, and purple respectively. (**b**) Schematic diagram for the exon/intron organization of cotton CAMTA genes. The green boxes and black lines indicate the exons and introns, respectively. (**c**) The conserved protein motifs in the cotton CAMTAs were identified using MEME tool. Each motif is indicated with a specific color.

The evolutionary relationships among cotton CAMTAs were inferred by constructing a separate ML tree with 1000 bootstraps. The tree topology, duplication nodes of CAMTA paralogues in the ML, exon/intron organization, and conserved motifs allowed us to classify the cotton CAMTAs into seven subfamilies (I-VII) with highest bootstraps. The genes within the same subfamily had a high identity (>80%) to each other, especially for those with the orthologous relationship, indicating their divergent evolution from a common ancestor or probable origin from gene duplication events (Fig. 5a).

To comprehend the structural diversity of cotton CAMTAs, we investigated their exon/intron patterns by comparing coding sequences and the corresponding genomic DNA sequences. The gene structure of cotton CAMTA genes showed group-specific exon/intron patterns. The number of introns varied from 9 to 15 in most of the CAMTA genes of *Gossypium* species. Intron number ranged from 11 to 15, 10 to 12 and 9 to 15 in GaCAMTAs, GrCAMTAs, and GhCAMTAs, respectively (Fig. 5b, Table 1). To investigate, whether the exon/intron structure is consistent with phylogenetic subfamilies, the gene structure of cotton CAMTAs was compared. Most of the cotton CAMTAs within the same subfamily shared similar exon/intron distribution patterns in terms of intron number and exon length. For instance, CAMTA gene in subfamily I and VI contained 12 introns with 13 exons of similar length, whereas members within subfamily VII contained 15 introns, except for GrCAMTA5.3, which possesses 12 introns. MEME (Multiple Em for Motif Elicitation) was exploited to analyze the conserved motifs in CAMTA protein. Twenty putative conserved motifs were identified in the cotton CAMTAs. The InterProScan was employed to annotate these motifs. Motifs 1, 11 and 12 that hit the database were the conserved CG-1 domain. Motifs 2, 3 and 4 were the IQ-motif, Ankyrin repeat-containing domain, and immunoglobulin-like (Ig) fold, respectively (Supplementary Dataset S5). Motif 1 (the red motif; CG-1 domain) was present in all cotton CAMTAs and represents the conserved CAMTA domain. Most of the CAMTA family members with close evolutionary relationships and similar gene structure in the phylogenetic tree had identical motif compositions and hypothesized similar function (Fig. 5c).

### Expression profiles of GhCAMTAs

**Figure 6:**
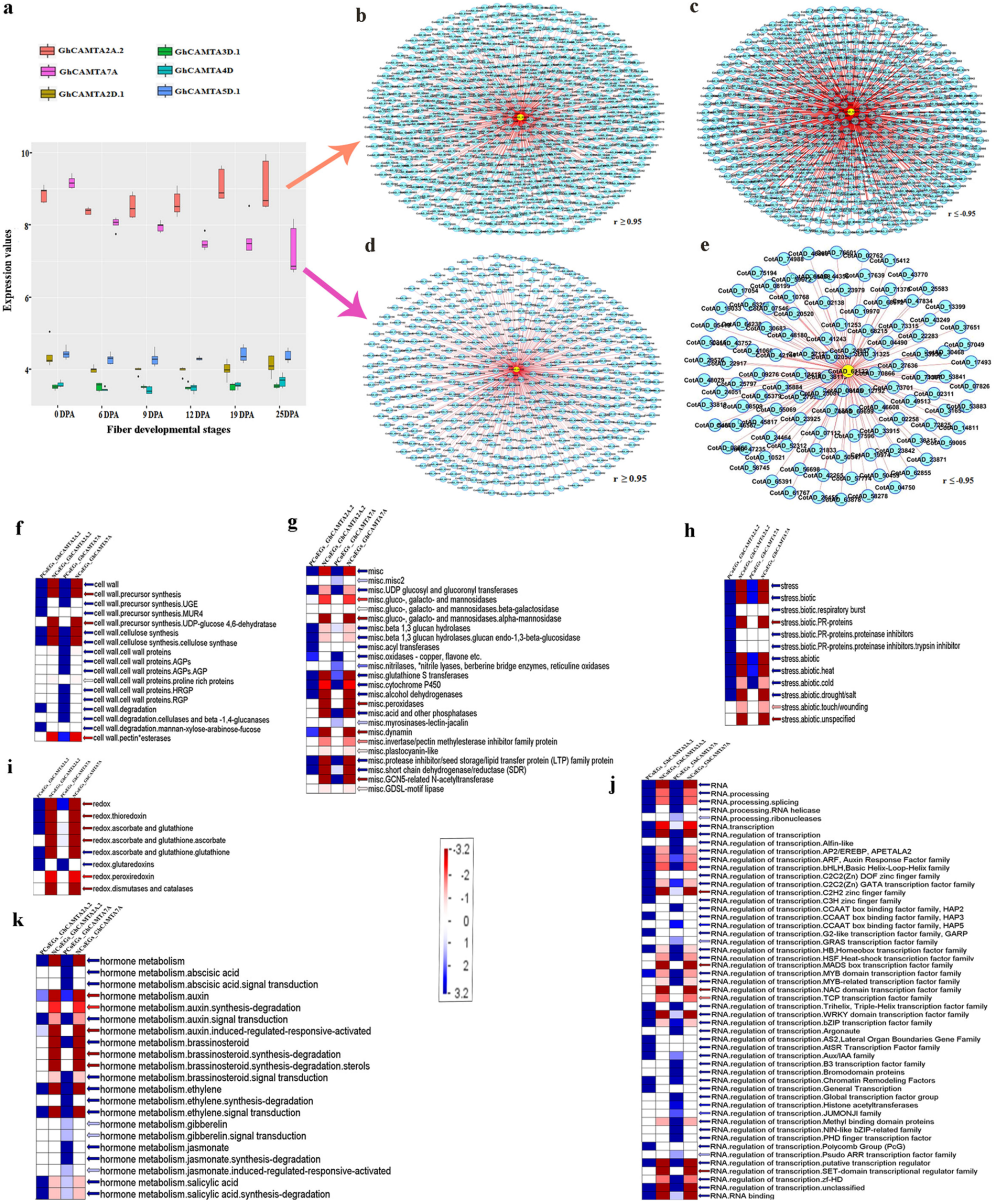
Expression profiles of six GhCAMTAs present in Affymetrix cotton chip at different fiber developmental stages and Co-expression network analysis of GhCAMTA2A.2 and GhCAMTA7A. (**a**) Variation in expression of 6 GhCAMTAs in different fiber developmental stages were visualized by box plot. Each GhCAMTAs in box plot were represented with different colors. The vertical axis represents expression values, while the horizontal axis corresponds to the different cotton fiber developmental stages. The central line for each box plot indicated median. The top and bottom edges of the box indicated the 25th and 75th percentiles. Gene co-expression network was constructed using cytoscape software. (**b**) PCoEGs and (**c**) NCoEGs with GhCAMTA2A.2. (**d**) PCoEGs and (**e**) NCoEGs with GhCAMTA7A at different fiber developmental stages. Circles (nodes) represent transcripts and lines (edges) represent significant transcriptional interaction between GhCAMTAs and transcripts. MapMan based functional classification of PCoEGs and NCoEGs (**f**) Cell wall (**g**) ROS (**h**) Stress (**i**) Redox (**j**) Transcriptional regulation (**k**) Hormone metabolism. BINs coloured in green and red are significantly positively and negatively regulated groups respectively. Expression values in log_2_ scale indicated by the scale bar in the middle.

To investigate the potential functions of GhCAMTAs, we performed the reciprocal BLAST of nine GhCAMTAs with Affymetrix cotton chip. Out of nine GhCAMTAs, six CAMTAs were mapped with the cotton chip with high identity (>80%) and sequence similarity. The expression profiles of these six GhCAMTAs were investigated from previously reported microarray data of five genotypes and six cotton fiber developmental stages (0, 6, 9, 12, 19 and 25 DPA; day post anthesis)^38^. CAMTAs such as GhCAMTA2D.1 (Ghi.3380.2.S1_s_at), GhCAMTA3D.1 (Gra.1342.2.A1_s_at), GhCAMTA4D (GraAffx.30859.1.S1_at), and GhCAMTA5D.1 (GraAffx.11842.1.A1_at) showed very low expression in all the cotton fiber developmental stages in all the genotypes. Among all the GhCAMTAs specifically, GhCAMTA2A.2 (GhiAffx.40335.1.S1_at) and GhCAMTA7A (GhiAffx.26204.1.A1_at) had high expression in initiation and secondary cell wall synthesis (SCW) stages (0 to 25 DPA) as compared to others (Fig. 6a). The expression of GhCAMATA2A.2 was significantly higher in elongation (9 and 12 DPA) and SCW (19 and 25 DPA) stages. Conversely, GhCAMTA7A was highly expressed in initiation stage (0 DPA) (Fig. 6a). These results implied that GhCAMTA2A.2 and GhCAMTA7A might play an important role in fiber development i.e., initiation, elongation, and SCW stages.

The quantitative real-time PCR (qRT-PCR) at the representative stages of fiber development (0 to 25 DPA) validated the expression profiles of two highly expressed cotton CAMTAs (GhCAMTA2A.2, GhCAMTA7A) and least expressing CAMTA (GhCAMTA3D.1). The GhCAMTA7A expression found decreased from 0 to 12 DPA and slightly increased at 19 and further decreased at 25 DPA. However, GhCAMTA2A.2 showed a subsidiary increase in the expression pattern during SCW stage. Conversely, GhCAMTA3D.1 showed lower expression at all representative stages of fiber development, by the in silico expression analysis (Supplementary Fig. S5).

### Co-expression network analysis of GhCAMTA2A.2 and GhCAMTA7A at different fiber developmental stages

The significant amount of RNA-sequencing (RNA-seq) data for several fiber developmental stages of *G. hirsutum* is available publically. To identify the co-expressing genes with GhCAMTA2A.2 and GhCAMTA7A, we analyzed a publicly available RNA-seq dataset of *G. hirsutum* that encompassed results from five different fiber developmental stages (0, 5, 10, 20 and 25 DPA). The Log_2_ transformed FPKM (Fragments Per Kilobase of transcript per Million mapped reads) values were used in Cytoscape version 2.8.1 to identify the co-expressing genes. A total of 651 genes positively co-expressed (PCoEGs) (r ≥ 0.95) (Fig. 6b and Supplementary Dataset S6) and 575 genes negatively co-expressed genes (NCoEGs) (r ≤ −0.95) with GhCAMTA2A.2 (Fig. 6c and Supplementary Dataset S6). Similarly, 504 genes were PCoEGs (r ≥ 0.95) and 114 genes were NCoEGs (r ≤ −0.95) (Fig. 6d and Supplementary Dataset S7) with GhCAMTA7A (Fig. 6e and Supplementary Dataset S7).

PCoEGs and NCoEGs were subjected to MapMan (http://gabi.rzpd.de/projects/MapMan/) analysis to identify enrichment of functional and molecular categories. The MapMan analysis revealed that several GhCAMTA2A.2 and GhCAMTA7A positively co-expressed genes belong to the cell wall and its precursor synthesis (Fig. 6f). Further, positively co-expressed genes also belong to oxidative stress including oxidases, cytochrome P450, alcohol dehydrogenase, short-chain dehydrogenase/reductase (SDR) (Fig. 6g), respiratory burst (Fig. 6h) and redox pathways including ascorbate, glutathione, and glutaredoxin (Fig. 6i). The transcription factors belonging to class AP2/EREBP, ARF, bHLH, MYB, WRKY, bZIP, Aux/IAA and phytohormones including abscisic acid, auxin, brassinosteroid, ethylene, gibberellins, jasmonate and salicylic acid also belonged to positively co-expressed genes with these two CAMTAs (Fig. 6j,k). In contrast, the NCoEGs of GhCAMTA2A.2 and GhCAMTA7A mainly belong to peroxidases, dynamin (Fig. 6g), thioredoxin, peroxiredoxin, dismutases and catalases (Fig. 6i). The transcription factors in NCoEGs belongs to C2H2 zinc finger family, MADS, E2F/DP, NAC and TCP (Fig. 6j). There were other functional categories which were found enriched with these positively and negatively co-expressed genes which belong to amino acid metabolism, co-factor and vitamin metabolism, development, nucleotide metabolism, secondary metabolism, carbohydrate metabolism, photosynthesis, lipid metabolism, glycolysis, protein metabolism, signaling, and transport (Supplementary Fig. S6).

**Figure 7:**
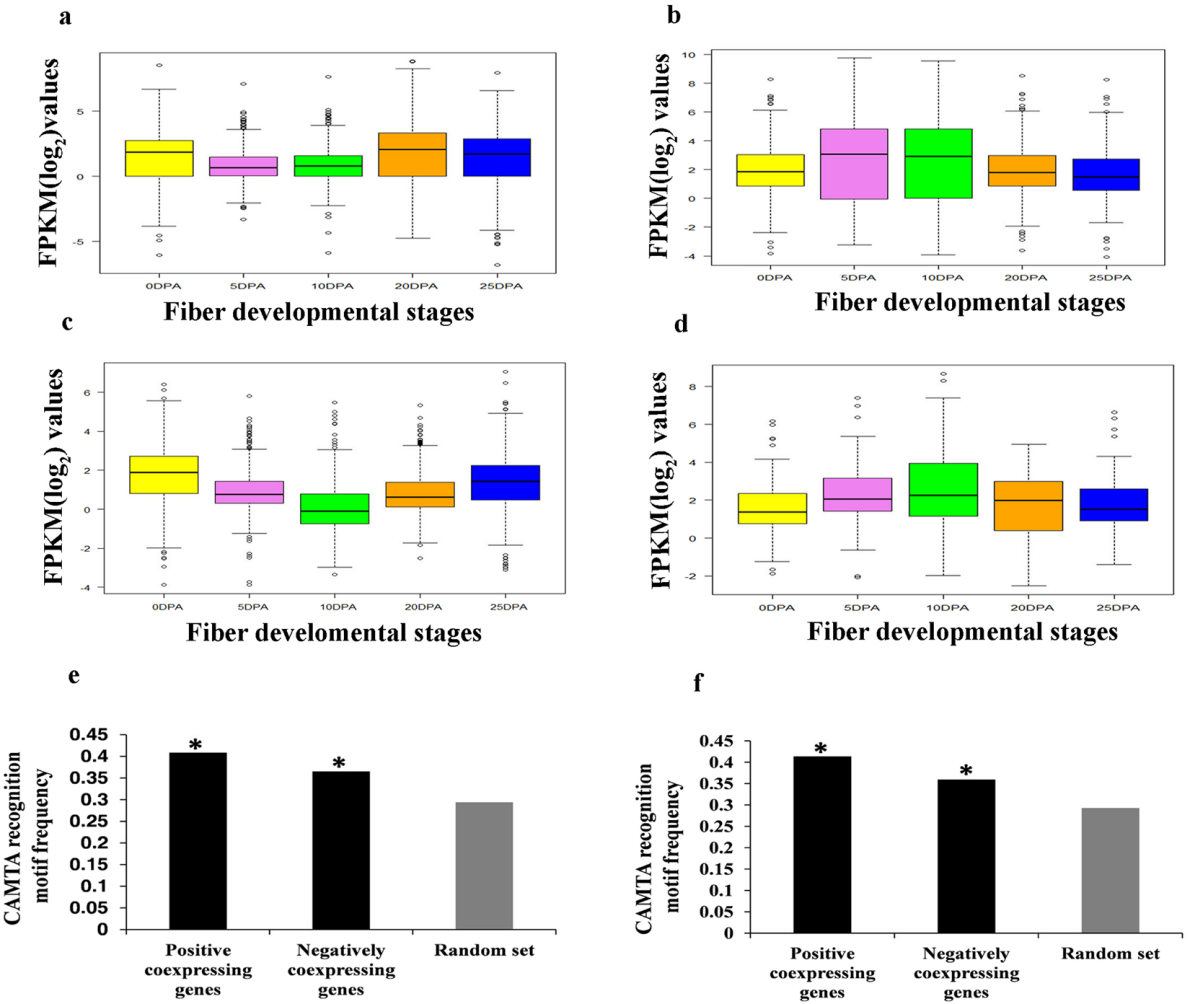
Expression and promoter region analysis of PCoEGs and NCoEGs with GhCAMTA2A.2 and GhCAMTA7A. Box plot showing variation in expression level of (**a**) PCoEGs and (**b**) NCoEGs with GhCAMTA2A.2 (**c**) PCoEGs and (**d**) NCoEGs with GhCAMTA7A at different fiber developmental stages. Graph (**e**) and (**f**) showing the frequency of CAMTA recognition motif (MCGCGB/MCGTGT) in randomly selected transcripts, PCoEGs and NCoEGs with GhCAMTA2A.2 and GhCAMTA7A, respectively. The asterisks represent significant differences (Fisher’s exact test, P < 0.05).

We next analyzed the cumulative expression of PCoEGs and NCoEGs of GhCAMTA2A.2 and GhCAMTA7A. The PCoEGs with GhCAMTA2A.2 show significantly higher cumulative expression during SCW stage precisely at 20 and 25 DPA (Fig. 7a), while the cumulative expression of NCoEGs with GhCAMTA2A.2 was lower at SCW (Fig. 7b). Coherently, for PCoEGs with GhCAMTA7A showed higher cumulative expression at 0 DPA which subsequently declined till 10 DPA and further increased at 20 and 25 DPA (Fig. 7c). In complete contrast, the cumulative expression was highest at 10 DPA in NCoEGs with GhCAMTA7A (Fig. 7d).

Next, promoter regions (1000 bp upstream) of PCoEGs (651) and NCoEGs (575) with GhCAMTA2A.2 as well as PCoEGs (504) and NCoEGs (114) with GhCAMTA7A were analyzed to identify the conservation of CAMTA binding motifs (MCGCGB/MCGTGT). A random promoter set (700) was selected as control of those genes which do not co-expressed either with GhCAMTA2A.2 or GhCAMTA7A. The frequency of occurrence of CAMTA binding motifs in PCoEGs and NCoEGs with GhCAMTA2A.2 was 0.40 and 0.36 which was significantly higher as compared to the control (0.29) (Fig. 7e and Supplementary Dataset S6). Similarly, PCoEGs and NCoEGs with GhCAMTA7A showed significantly higher frequency of occurrence of these motifs (0.41 and 0.35, respectively) than control (0.29) (Fig. 7f and Supplementary Dataset S7). Hence, this study affirms indicates that presence of CAMTA binding motifs in the upstream elements of PCoEGs results in their regulation by GhCAMTA2A.2 and GhCAMTA7A. As evident GhCAMTA2A.2 and GhCAMTA7A also regulates NCoEGs, however, the underpinning mechanisms of fiber development by GhCAMTA2A.2 and GhCAMTA7A remain elusive and demands further experimental exploration.

### Correlation analysis of highly and least expressing CAMTAs with different fiber quality traits

**Figure 8:**
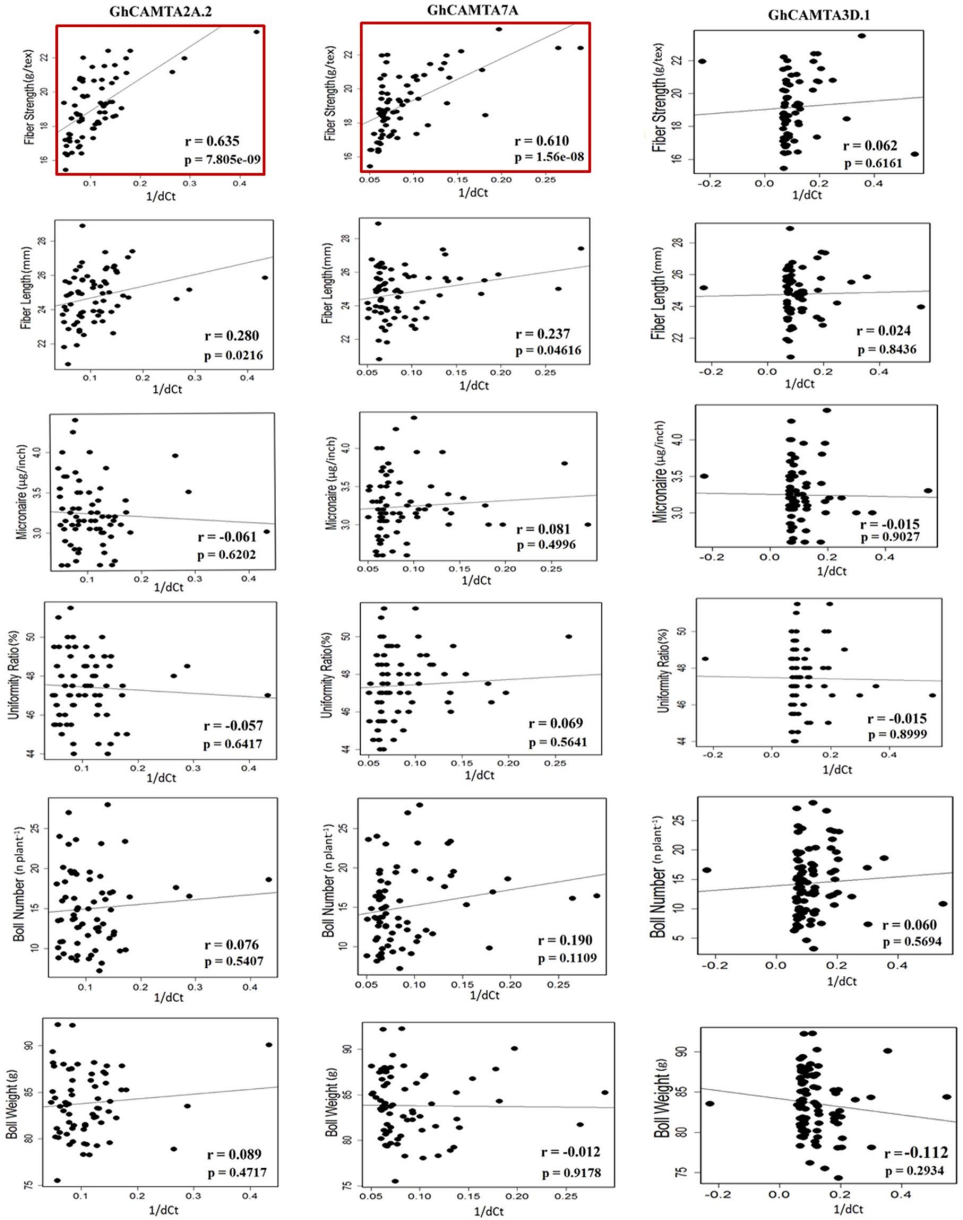
Correlation analysis in 100 genotypes of *G. hirsutum* at 0 DPA. Different fiber quality traits values plotted against the expression values (1/dCt) of highly (GhCAMTA2A.2 and GhCAMTA7A) and least expressing CAMTA (GhCAMTA3D.1). The r and p represent the Pearson correlation coefficient and p-value, respectively. Red box represents significant positive correlation of GhCAMTA2A.2 and GhCAMTA7A with fiber strength.

The correlation was established between the expression of CAMTAs and fiber quality traits to understand the functional relevance of CAMTAs during cotton fiber development. The quantitative real-time PCR (qRT-PCR) of highly expressing CAMTAs (GhCAMTA2A.2 and GhCAMTA7A) and least expressing CAMTAs (GhCAMTA3D.1) was carried out in 67 genotypes of *G. hirsutum* at 0 DPA. We also estimated fiber quality traits such as fiber length (FL), fiber strength (FS), uniformity ratio (UR), micronaire (MIC), boll number (BN) and boll weight (BW) in the same 67 genotypes. Pearson correlation coefficient (r) was estimated to check the correlations between highly expressing CAMTAs and fiber quality traits of genotypes. The results indicated that GhCAMTA2A.2 and GhCAMTA7A displayed significant positive correlation ~0.63 (p = 7.805e-09) and ~0.61 (p = 1.56e-08), respectively with fiber strength but not with other traits. However, no significant correlation was found in the least expressing GhCAMTA3D.1 with any of the fiber quality determining trait (Fig. 8). The results thus provide important evidence on the role of GhCAMTA2A.2 and GhCAMTA7A in the cotton fiber development.

## Discussion

The recent availability of *G. hirsutum*^31^ and its diploid progenitor genomes^29,30^ allowed us to perform a comprehensive analysis of CAMTAs. *G. hirsutum* has longer fibers than *G. arboreum* and *G. raimondii* possibly due to the genome and associative doubling of fiber-related genes^39^. To explore the probable roles of cotton CAMTAs in fiber development, we undertook a comprehensive genome-wide characterization, expression, and co-expression network analysis. Our study identified a total of 6, 7 and 9 CAMTA genes in *G. arboreum*, *G. raimondii*, and *G. hirsutum*, respectively (Table 1). The *G. arboretum* has a similar number of CAMTA genes as in Arabidopsis (6), whereas *G. raimondii* contains the 7 CAMTAs in consistence with the proportion of the predicted genes in their genome. For example, *G. raimondii* genome (40,976 genes)^30^ is about 1.6 times that in Arabidopsis (25,498 genes)^40^. Although the genome size of *G. arboreum* (1,746 Mb)^29^ is approximately two fold of *G. raimondii* genome (885 Mb)^29^, *G. raimondii* contains a higher number of CAMTA genes as compared to *G. arboreum*. This higher number might be due to more retrotransposons insertion, gene loss, disrupted genes, structural rearrangements, sequence divergence in A-genome resulting in a fewer number of CAMTA gene^31^. It is noteworthy that A- genome evolution is more ancient than that of D-genome. Therefore, the difference in some CAMTA genes may be due to the extent of evolutionary divergence that is more in A-genome than D- genome^29^. The *G. hirsutum* contains 9 CAMTA genes, due to its allotetraploid nature. This expansion appeared because of whole-genome duplication events in cotton lineage and may be due to the transposable elements which represented a significant component of *Gossypium* genome^41^.

Sequence analysis revealed that apart from some non-TIG cotton CAMTAs, all of the cotton CAMTAs contained multifunctional conserved domains of CAMTAs and were localized in the nucleus (Fig. 1). The CaMBD interacts with CaM in a Ca^2+^-dependent manner, while the IQ domain binds with CaM in a Ca^2+^- independent way^1–3,5,34,35^. It is interesting to note that all the cotton CAMTAs contain CaMB domain, which was located at conserved positions adjacent to the IQ motifs, indicating that cotton CAMTAs may interact with CaM in a Ca^2+^-dependent and Ca^2+^- independent manner, respectively.

The phylogenetic tree of cotton CAMTAs with different plant species indicated that the major groups or subgroups contain orthologs from Arabidopsis, *T. cacao*, and other plant species, proposing a similar function of cotton CAMTAs with CAMTAs of different plant species which emerged from monocot-eudicot split (Fig. 3). Conversely, group II CAMTAs that were the result of recent species-specific duplication events lead to independent functional diversification (Supplementary Figs S2 and S3). Ortholog pairs of group II CAMTAs experienced faster evolution as compared to other CAMTAs, signifying their functional divergence in cotton, suggesting that group II CAMTAs may have a precise role in *Gossypium* species (Supplementary Table S2).

Mounting evidence suggests that expansion of gene families caused by the gene duplication event is one of the major evolutionary mechanisms directing to functional diversification and speciation^42^. Chromosomal distribution studies speculated that the expansion of the cotton CAMTAs arose from a segmental duplication (Fig. 4a–c) and purifying selection predominated across the duplicated CAMTA genes. Purifying selection possibly eliminates deleterious loss-of-function mutations, fixing a new duplicate gene and improving functional alleles at both duplicate loci^43^. Recent duplication events in cotton CAMTAs implied their morphological, ecological and physiological diversification^44^. *G. arboreum* and *G. raimondii* diverged 2–13 MYA and recombined to form *G. hirsutum* 1–2 MYA^29,31^. The duplication time of GaCAMTAs (13.02–15.03 MYA), GrCAMTAs (12.08–13.68 MYA) and GhCAMTAs (13.04 MYA), suggests that duplication events in cotton CAMTA families were more ancient than that of both diploid species divergence and polyploid formation. This duplication may assist to the unique functions of CAMTA in cotton i.e., cotton fiber development. The duplication time of GaCAMTAs and GrCAMTAs was around 13.4 MYA, which possibly occurred after their divergence from *T. cacao* (33MYA)^29^ and Arabidopsis (93 MYA)^45^ but before the reunion of A and D genome diploids that resulted in allotetraploid cotton^31^ (Table 2).

Cotton CAMTAs were classified into seven subfamilies (I-VII) based on their phylogenetic relationship, gene structure and motif distribution pattern (Fig. 5a–c). Subfamily I and IV indicate that counterpart of CAMTA protein in A_T_ (GhCAMTA2A.2/GhCAMT7A) and D_T_ (GhCAMTA2D.1/GhCAMTA7D) subgenome of AD genome, comes from both the progenitor genome, i.e., A genome (GaCAMTA2.2 /GaCAMTA7) and D genome (GrCAMTA2.2/GaCAMTA7), respectively. Cotton CAMTAs present in subfamily II shows that counterpart of CAMTA protein in A_T_ subgenome (GhCAMTA2A.1) comes from A genome (GaCAMTA2.1) while ortholog of GrCAMTA2.1 gene had lost in AD genome during evolution. Subfamily III demonstrated that counterpart of CAMTA protein in AD genome (GhCAMTA3D.1) either duplicated or diverged from D genome (GrCAMTA3.1) and lost in *G*. *arboreum*. Similarly, GhCAMTA4D of D_T_ subgenome diverged from GaCAMTA4 in subfamily V. In subfamily VI, GhCAMTA5D.1 diverged from GrCAMTA5.3 and counterpart of GaCAMTA5.2 protein was lost in D genome. Subfamily VII suggests that counterpart of CAMTA protein of A (GaCAMTA5.1) and D (GrCAMTA5.1) genome were absent in AD genome (Fig. 5a). The CAMTA genes in the same subfamily had the similar gene and motif structure representing their similar subfamily-specific function (Fig. 5b,c). The highly conserved sequences of CAMTAs within the same group demonstrated that they were subject to duplication during evolution.

Fiber-specific expression analysis of GhCAMTAs showed that they expressed in fiber development stages but exhibited differential expression profiles (Fig. 6a). Expression profile of GhCAMTA2A.2 and GhCAMTA7A suggest that they express at initiation and SCW stages, respectively. They might be involved in regulating complex gene networks of fiber development and could be suitable targets for genetic engineering approaches aimed to improve cotton fiber. PCoEGs and NCoEGs with GhCAMTA2A.2 and GhCAMTA7A (Supplementary Dataset S6 and Supplementary Dataset S7), belong to Fatty acid desaturase^46^, FAD linked oxidase^46^, Kinesin^47^, Protein kinase^46^, AUX/IAA protein^46,48^, ABC transporter-like^49^, DNA-binding WRKY^49^, MYB^47^, Homeodomain^47^, Zinc finger^46^, Leucine-rich repeat^46^ and Cellulose synthase^49^ protein families which have been previously reported to have imperative roles in the cotton fiber development^46,47,50^. It is important to note that CAMTAs regulate various stress and ROS response in plants^18^. ROS response, as well as redox state, is significant for the fiber development in cotton^51,52^. Thus, identifications of various genes belonging to ROS in positively and negatively co-expressed genes of GhCAMTA2A.2 and GhCAMTA7A further support the importance of these transcription factors in fiber development (Fig. 6g). Additionally, CAMTAs are known to interact with various phytohormones including ethylene^15^, ABA^19^, salicylic acid^21^, auxin^13^, and jasmonate^21^. These phytohormones also play critical roles in cotton fiber development^53,54^. Thus identification of genes belonging to these phytohormone categories in PCoEGs and NCoEGs (Fig. 6k) also support the role of GhCAMTA2A.2 and GhCAMTA7A in the fiber development. Finally, identification of transcription factors previously implicated in the fiber development such as MYB^55^, TCP^56^, NAC^57^ and WRKY^58^ in the PCoEGs and NCoEGs (Fig. 6j) substantiate the role of CAMTAs in cotton fiber development. We identified a significant enrichment of CAMTA-motifs in the promoters of PCoEGs and NCoEGs of GhCAMTA2A.2 and GhCAMTA7A (Fig. 7e,f). Our results suggest that CAMTA can act as both positive and negative regulator of gene expression. AtCAMTA3 is a positive regulator of CBF2 expression, and negative regulator of SA mediated immune response^5,20^. The higher conservation of CAMTA binding motif even in the NCoEGs is thus not surprising.

Since fiber strength is the key trait of fiber quality, the significant positive correlation between the expression of the two discussed CAMTAs and fiber strength suggest that these CAMTAs might be responsible for elite fiber qualities (Fig. 8). Previous studies demonstrated that the fiber strength commonly related to the strengthening of cell wall^59^. Interestingly, identification of cell wall related genes such as ABC transporter-like^47,60,61^, arabinogalactan peptide (AGP)^62,63^, alpha-1,4-glucan-protein synthase^64^, calcium-binding EF-hand protein^61^, cellulose synthase^63^, glutathione peroxidase^65^, glycoside hydrolase^66^, glycosyl transferase^67–69^, extensin^62^, UDP-glucuronosyl/UDP-glucosyltransferase^70^, small GTPase superfamily^71^, kinesin^72^, leucine-rich repeat^61^, thaumatin^73^, tubulin^74^, and WD40 repeat^75^ in PCoEGs and NCoEGs, emphasizing their potential roles in regulating cell wall integrity and thus fiber quality (Supplementary Dataset S8). However, the detailed molecular investigation is needed further to establish a link between CAMTAs and fiber quality traits.

This study has provided us evidence for an involvement of CAMTAs in cotton fiber development. However, more experimental exploration is needed to understand the structural-functional relationship of CAMTA family members in cotton and their involvement in fiber development.

## Methods

### Identification of CAMTA gene family in *Gossypium* species

The whole-genome peptide sequence dataset of *G. arboreum* and *G. hirsutum* was downloaded from cotton genome project (http://cgp.genomics.org.cn/page/species/index.jsp) and of *G. raimondii* from Phytozome (http://www.phytozome.net/)^76^. A total of 465 CAMTA domain sequences from 75 plant species were obtained from Plant Transcription Factor Database (http://planttfdb.cbi.pku.edu.cn/)^77^ and utilized to construct Hidden Markov Model (HMM) profile. Further, HMM profile of the CAMTA domains (CG-1, TIG domain, Ankyrin repeats, IQ) was employed as a query to identify CAMTA gene family members using HMMER (V3.0)^78^ software. All hits were subjected to the Pfam (http://pfam.xfam.org/) and InterProScan (http://www.ebi.ac.uk/interpro/search/sequence-search) database to verify the presence of conserved domains. Finally, ProtParam (http://web.expasy.org/protparam/) tool was used to compute the physicochemical parameters of cotton CAMTA proteins.

### Subcellular localization, CAMTA protein domain structure and NLS prediction

Online available server CELLO v.2.5 (http://cello.life.nctu.edu.tw/) was used to predict the possible subcellular locations of all the cotton CAMTA proteins. Protein domain structures were analyzed in Pfam database, and a schematic diagram of protein functional domain was constructed using Illustrator for Biological sequences software (http://ibs.biocuckoo.org/)^79^. NLS was searched by Motif scan (http://myhits.isb-sib.ch/cgi-bin/motif_scan). The CaMBD was analyzed using Calmodulin Target Database (http://calcium.uhnres.utoronto.ca/ctdb/ctdb/home.html).

### Multiple sequence alignment, classification and phylogenetic tree construction of CAMTA protein sequence

CAMTA protein sequences; from the 17 plant species known to have publicly available complete genome sequences; were extracted. The multiple sequence alignment of these protein sequences with identified cotton CAMTAs were carried out by cluster X program (http://www.clustal.org/)^80^ with default parameters. These aligned sequences were used for the construction of the phylogenetic tree. MEGA 5.2 software (http://www.megasoftware.net/)^81^ was employed to construct an unrooted phylogenetic tree using ML method with the following parameters: JTT model, pairwise gap deletion, and 1,000 iterations were used to calculate bootstrap values. CAMTA gene family members were classified based on Arabidopsis nomenclature by using phylogenetic approach. Additionally, a separate phylogenetic tree was constructed with all the CAMTA protein sequences of *G. arboreum, G. raimondii*, and *G. hirsutum* for further analysis.

### Chromosomal location and gene duplication analysis

The precise physical positions of all *Gossypium* CAMTA genes on chromosomes were obtained through BLASTN search against the Cotton Genome project and Phytozome databases. All *Gossypium* CAMTA genes were mapped on the chromosome using Mapinspect software.

The paralogous CAMTA genes were identified in *G. arboreum*, *G. raimondii*, and *G. hirsutum* by using reciprocal blast with e-value < 10^−5^ to understand the evolutionary mechanism of CAMTA gene family in *Gossypium* species. Paralogs were defined by shared aligned region covering > 70% of the longer sequence and the similarity of the aligned regions >70%^82^. Also, Ka/Ks analysis of orthologs and paralogs sequences was performed by using PAL2NAL and Codeml program^83^, which was further used to calculate the approximate date of duplication and divergence events with the formula T = Ks/2λ, assuming clock-like rate (λ) of 1.5 synonymous substitutions per 10^−8^ years for cotton^84,85^. Additionally, the Ka/Ks ratio was used to show the selection pressure for the duplicated CAMTA genes. A Ka/Ks ratio <1, >1 and =1 indicates negative (purifying selection), positive, and neutral evolution, respectively^86^.

### Synteny analysis of cotton CAMTA genes with *T. cacao*, *C. sinensis* and *A. thaliana*

We identified the orthologs of cotton CAMTA genes between* G. hirsutum* vs. *T. cacao*, *G. hirsutum* vs. *C. sinensis*, *G. hirsutum* vs. *A. thaliana*, *G. raimondii* vs. *T. cacao*, *G. raimondii* vs. *C. sinensis*, *G. raimondii* vs. *A. thaliana*, *G. arboreum* vs. *T. cacao*, *G. arboreum* vs. *C. sinensis* as well as *G. arboreum* and *A. thaliana* using reciprocal blast with e-value 10^−5^. According to the reciprocal blast output, duplication events were identified using the McScanX software^87^.

### Gene structure and conserved motif analysis of cotton CAMTAs

The gene structures of each identified CAMTA genes were obtained by comparing predicted CAMTA coding sequences with their corresponding genomic sequences using GSDS online tool (Gene Structure Display Server; http://gsds.cbi.pku.edu.cn/)^88^.

Online MEME tool (http://meme.nbcr.net/meme/)^89^ was used for identification of conserved protein motifs in the CAMTA protein sequences. The following parameters were used: zero or one per sequence; the optimum width from 6 to 300; maximum number of motifs to find 20. Further, these motifs were annotated by using an Interproscan program.

### Expression profile of cotton CAMTA genes during different fiber developmental stages

The stage-specific expression pattern of cotton CAMTA genes was analyzed by using our previously reported microarray profiling data of *G. hirsutum* at various fiber developmental stages such as initiation (0DPA), elongation (6, 9, 12DPA) and SCW (19 & 25 DPA)^38^. Coding sequences of 9 GhCAMTA genes were subjected to reciprocal blast with Affymetrix cotton chip. The average normalized intensity values of these 6 GhCAMTA genes from microarray data were utilized for generating the box plot. ggplot2 (https://cran.r-project.org/web/packages/ggplot2/) package in R version 3.1.3 was used to construct the box plot.

### Co-expression network analysis of GhCAMTA2A.2 and GhCAMTA7A

The high-throughput RNA-sequencing (RNA-seq) data of *G. hirsutum* in different fiber developmental stages at 0, 5, 10, 20, and 25 DPA were downloaded from the National Center for Biotechnology Information Short Read Archive (http://www.ncbi.nlm.nih.gov/sra/) with the accession numbers SRX797909, SRX797917, SRX797918, SRX797919, and SRX797920, respectively^31^. Reads from transcriptome dataset (0, 5, 10, 20, and 25 DPA) were mapped on *G. hirsutum* genome using the STAR aligner^90^ (version 2.5.3a) with default parameters separately. Assembly of data and transcript abundance of each gene was calculated by the fragments per kilobase of exon model per million mapped reads (FPKM) with Cufflinks software (http://cufflnks.cbcb.umd.edu/). The Log_2_ FPKM values were used for generating gene coexpression network using the “Expression Correlation Networks” (http://apps.cytoscape.org/apps/expressioncorrelation) plugins in Cytoscape version 2.8.1. This plugin calculates positive Pearson correlation (default r ≥ 0.95) as well as “anti-correlation” or negative Pearson correlation (default r ≤ −0.95) among the interacting members of a network. Furthermore, network visualization was carried out in Cytoscape by applying the force-directed layout, where nodes (circles) in a network represent genes and edges (links) represent a significant interaction between the expression levels of genes across all fiber developmental stages (gene correlation network).

### Pathway analysis of positively and negatively co-expressed genes with GhCAMTA2A.2 and GhCAMTA7A

MapMan software version 3.5.1 (http://gabi.rzpd.de/projects/MapMan/) was used for identification of significant functional categories or metabolic pathways of positively and negatively co-expressed genes with GhCAMTA2A.2 and GhCAMTA7A. To identify functional categories (BINSs, subBINs) enriched in these genes; average statistical test followed by the Benjamini Hochberg (multiple correction test) was used.

### RNA isolation and real-time quantitative RT-PCR

The primers used in the real-time analysis were designed from CDS sequences of respective genes. Reference gene, *Ubiquitin* and respective genes primers were designed using Primer Express^®^ Software v2.0 (Applied Biosystems, USA). The total RNA was isolated from different stages of cotton fiber development (0 DPA, 6 DPA, 9 DPA, 12 DPA, 19 DPA and 25 DPA) using SIGMA Spectrum plant total RNA kit following the manufacturer’s protocol. After DNase (Ambion) treatment, the integrity of RNA was checked by electrophoresis and the RNA was quantified for cDNA synthesis on NanoDrop ND-1000 Spectrophotometer. One μ g of total RNA was used for first-strand cDNA synthesis using the Superscript II RT kit (Invitrogen) following the manufacturer’s instructions. The real-time PCR was performed employing 7500 Real-Time PCR System (Applied Biosystems, USA). PCR cycles 95 °C for 10 sec followed by 35 cycles of 95 °C for 10 sec and 60 °C for 20 sec were performed in 96-well optical reaction plates (Applied Biosystems). The specificity of the amplicon was assesses by its melting curve after 35 cycles at 60–90 °C. The relative gene expression levels were calculated in terms of comparative fold change following 2^−ΔΔct^ method. Statistical analysis was carried on two biological replicates (three technical replicates per biological sample) for each fiber development stage mentioned earlier. The list of primers used in qRT-PCR is given in Supplementary Table S3.

### Genetic material and fiber quality measurement

In the present study, 67 upland cotton (*G. hirsutum*) genotypes were utilized which were made available from Tierra Seed Science Pvt. Ltd, Hyderabad, India. The experiment was laid out in Random block design with three replications, each with 20 plants. All traditional agronomic practices were applied during the plant growing seasons. Five plants were randomly selected from each plot and data on following 6 traits were recorded on 67 genotypes- (i) fiber strength (FS) (ii) fiber length (FL), (iii) micronair (MIC), (iv) uniformity ratio (UR) (v) boll number (BN), and (vi) boll weight (BW). Fiber quality in terms of FL, UR, MIC and FS were estimated at Central Institute for Research on Cotton Technology (CIRCOT), Mumbai, India. The quantitative real-time PCR (qRT-PCR) with GhCAMTA2A.2, GhCAMTA7A and GhCAMTA3D.1was performed in same 67 genotypes of *G. hirsutum* at 0 DPA. Pearson’s correlation analysis was performed to calculate the correlation coefficient (r) between expression of these CAMTAs and above mentioned traits using R software.

## Electronic supplementary material

Supplementary Information

Dataset S1

Dataset S2

Dataset S3

Dataset S4

Dataset S5

Dataset S6

Dataset S7

Dataset S8
