# Supplementary material for: Genome-wide comparative and evolutionary analysis of Calmodulin-binding Transcription Activator (CAMTA) family in Gossypium species
Source: Sci Rep. 2018 Apr 3;8:5573. doi: 10.1038/s41598-018-23846-w (PMC5882909; doi:10.1038/s41598-018-23846-w)
Supplement: Supplementary file 1 — Supplementary Information [file 41598_2018_23846_MOESM1_ESM.pdf]

**Supplementary information**

# Genome-wide comparative and evolutionary analysis of Calmodulin-binding Transcription Activator (CAMTA) family in *Gossypium* species

Poonam Pant[1, 2], Zahra Iqbal[1], Bhupendra Pandey[1, 2] and Samir V. Sawant[1,2*]

Council of Scientific and Industrial Research - National Botanical Research Institute (CSIR-NBRI), Rana Pratap Marg, Lucknow-226001, INDIA

[2]Academy of Scientific and Innovative Research (AcSIR), CSIR-NBRI Campus, Lucknow, India

**The email addresses for all the authors:**

Poonam Pant - ppant5700@gmail.com

Zahra Iqbal - zahra1608@gmail.com

Bhupendra Pandey - bhupendrakbhu@gmail.com

Samir V Sawant - samirsawant@nbri.res.in

***Corresponding author:**

Dr. Samir V. Sawant,
Senior Principal Scientist,
Plant Molecular Biology and Genetic Engineering Lab
CSIR-National Botanical Research Institute
Rana Pratap Marg, Lucknow-226001 (U.P.) INDIA
Ph: +91-522-2297947 (O), Fax: +91-522-2205839
Email-samirsawant@nbri.res.in

**Running Title -** CAMTA gene family in *Gossypium* species

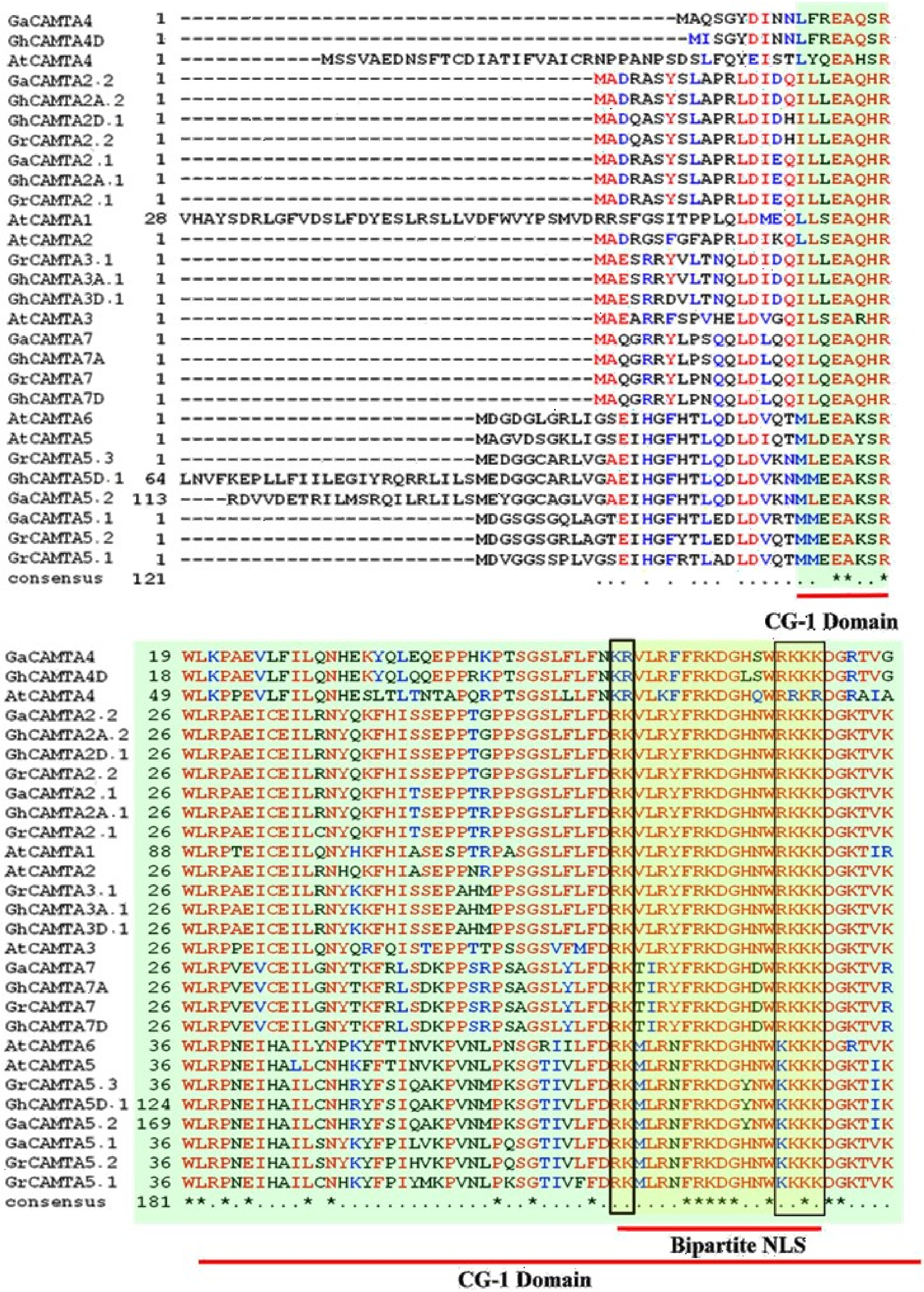
CG-1 Domain
Bipartite NLS
CG-1 Domain

```
GaCAMTA4      79 EAHERLKVGNVETLNCYYAHGEQNPSFQRRSYWMLDPA----------------------
GhCAMTA4D     78 EAHERLKVGNVETLNCYYAHGEQNPSFQRRSYWMLDPA----------------------
AtCAMTA4     109 EAHERLKVGNAEALNCYYAHGEQDPTFRRRIYWMLDPE----------------------
GaCAMTA2.2    86 EAHEKLKVGSIDVLHCYYAHGEENENFQRRSYWMLEQD----------------------
GhCAMTA2A.2   86 EAHEKLKVGSIDVLHCYYAHGEENENFQRRSYWMLEQD----------------------
GhCAMTA2D.1   86 EAHEKLKVGSIDVLHCYYAHGEENENFQRRSYWMLEQD----------------------
GrCAMTA2.2    86 EAHEKLKVGSIDVLHCYYAHGEENENFQRRSYWMLEQD----------------------
GaCAMTA2.1    86 EAHEKLKVGSVDMLHCYYAHGEENENFQRRSYWMLEPD----------------------
GhCAMTA2A.1   86 EAHEKLKVGSVDMLHCYYAHGEENENFQRRSYWMLEPD----------------------
GrCAMTA2.1    86 EAHEKLKVGSVDKLHCYYAHGEENENFQRRSYWMLEPD----------------------
AtCAMTA1     148 EAHEKLKVGSIDVLHCYYAHGEANENFQRRCYWMLEQYYYRKASSHWVLVATLSLFSFGY
AtCAMTA2      86 EAHEKLKVGSIDVLHCYYAHGEDNENFQRRCYWMLEQD----------------------
GrCAMTA3.1    86 EAHERLKAGSIDVLHCYYAHGEENENFQRRSYWMLEED----------------------
GhCAMTA3A.1   86 EAHERLKAGSIDVLHCYYAHGEQNENFQRRSYWMLEED----------------------
GhCAMTA3D.1   86 EAHERLKAGSIDVLHCYYAHGEENENFQRRSYWMLEED----------------------
AtCAMTA3      86 EAHERLKAGSVDVLHCYYAHGQDNENFQRRSYWLLQEE----------------------
GaCAMTA7      86 EAHEKLKIGSVDVLHCYYAHGQFNENFQRRCYWMLDGQ----------------------
GhCAMTA7A     86 EAHEKLKIGSVDVLHCYYAHGQFNENFQRRCYWMLDGQ----------------------
GrCAMTA7      86 EAHEKLKIGSVDVLHCYYAHGQFNENFQRRCYWMLDGQ----------------------
GhCAMTA7D     86 EAHEKLKIGSVDVLHCYYAHGQFNENFQRRCYWMLDGQ----------------------
AtCAMTA6      96 EAHEHLKVGNEERIHVYYAHGEDNTTFVRRCYWLLDKAR---------------------
AtCAMTA5      96 EAHEHLKVGNEERIHVYYAHGEDTPTFVRRCYWLLDKSQ---------------------
GrCAMTA5.3    96 EAHEHLKVGDKERIHVYYAHGEDNSTFVRRCYWLLDKSL---------------------
GhCAMTA5D.1  184 EAHEHLKVGDKERIHVYYAHGEDNSTFVRRCYWLLDKSL---------------------
GaCAMTA5.2   229 EAHEHLKVGDKERIHVYYAHGEDNSTFVR-------RSL---------------------
GaCAMTA5.1    96 EAHEHLKVGNEERIHVYYAHGLDNPTFVR-------RTL---------------------
GrCAMTA5.2    96 EAHEHLKVGNEERIHVYYAHGLDNPTFVRRCYWLLDKTL---------------------
GrCAMTA5.1    96 EAHEHLKVGNEERIHVYYAHGQDNPTFVRRCYWLLDKSL---------------------
consensus    241 ****.**.*.......*****.....*.*....... .
```

**CG-1 Domain**

```
GaCAMTA4     117 --------YEHIVLVHYREINEAKPCS--------ASTVHSPLSNSASTPSPISYTSQNP
GhCAMTA4D    116 --------YEHIVLVHYREINEAKPSS--------ASTVHSSLSNSARTPSPISYTSQNP
AtCAMTA4     147 --------YEHIVLVHYRDVSEREEGQ--------QTGGQVYQFAPILSTQNVSYNQYIG
GaCAMTA2.2   124 --------LTHIVFVHYLEVKGRTIGG----ISHVSNSQTSSPSTSSYPDSHTKAPSGNA
GhCAMTA2A.2  124 --------LTHIVFVHYLEVKGRTIGG----ISHVSNSQTSSPSTSSYPDSHTKAPSGNA
GhCAMTA2D.1  124 --------LTHIVFVHYLEVKGRTIGG----ISHVSNSQTSSPSTSSYPDSHTKAPSGNA
GrCAMTA2.2   124 --------LTHIVFVHYLEVKGRTIGG----ISHVSNSQTSSPSTSSYPDSHTKAPSGNA
GaCAMTA2.1   124 --------LMHIVFVHYLEVKGSRTIGGIRENSDLSNSQTSSLLTSSNSVTHTKEPSAHA
GhCAMTA2A.1  124 --------LMHIVFVHYLEVKGSRTIGGIRENSDLSNSQTSSLLTSSNSVTHTKEPSAHA
GrCAMTA2.1   124 --------LMHIVFVHYLEVKGSRTIGGIRENSDLSNSQTSSLLTSSNSVTHTKEPSAHA
AtCAMTA1     208 LRPSWVRHLMHIVFVHYLEVKG-------------NRTSIG-MKENNSNSVNGTASVNID
AtCAMTA2     124 --------LMHIVFVHYLEVKG-------------NRMSTSGTKENHSNSLSGTGSVNVD
GrCAMTA3.1   124 --------LSHIVLVHYRDVKGN--RTNFN-----RLKETEGGIPYSQEAVGIVPNSEVE
GhCAMTA3A.1  124 --------LSHIVLVHYRDVKGN--RTNFN-----RLKETEGAIPYSQEAVGIVPNSEVE
GhCAMTA3D.1  124 --------LSHIVLVHYRDVKGN--RTNFN-----RLKETEGTIPYYQEAVGIVPNSEVE
AtCAMTA3     124 --------LSHIVFVHYLEVKGSRVSTSFN-----RMQRTEDAARSPQETG-DALTSEHD
GaCAMTA7     124 --------FEHIVFVHYREVKEGYRSG-------ISCLLVDPGSQSESLQTGLAPSLTHE
GhCAMTA7A    124 --------FEHIVFVHYREVKEGYRSG-------ISCLLVDPGSQSESLQTGLAPSLTHE
GrCAMTA7     124 --------FEHIVFVHYREVKEGYRSG-------ISCLLADPGSLSESLQTGSTPSPAHE
GhCAMTA7D    124 --------FEHIVFVHYREVKEGYRSG-------ISCLLADPGSLSESLQTGSTPSPAHE
AtCAMTA6     135 ---------ENIVLVHYRDTQEAATTSGDS-------ISSPISVSEQTFPNRVAAEDIDT
AtCAMTA5     135 ---------EHIVLVHYRETHEVHAAPATP-------GNSYSSSITDHLSPKIVAEDTSS
GrCAMTA5.3   135 ---------EQIVLVHYRETKEVSLATHS-----------NSSSLTDQSTPLLVTEEFDS
GhCAMTA5D.1  223 ---------EQIVLVHYRETKEVSLATHS-----------NSSSLTDQSTPLLVTEEFDS
GaCAMTA5.2   261 ---------EQIVLVHYRETKEVSLATHS-----------NSSSLTDQSTPLLVTEEFDS
GaCAMTA5.1   128 ---------ENIVLVHYRETKE--GSPATP-------VNSNSCLTTDQSTPLLVTEDFDS
GrCAMTA5.2   135 ---------ENIVLVHYRETKE--GSPATP-------VNSNSCLTTDQSTPLLVMEDFDS
GrCAMTA5.1   135 ---------EHIVLVHYRETQESQGSPATP-------GNSNSSSITDQSTPLNVMEEFDS
consensus
             301         ...** ***.....      .          ..  .. .  . .......  .    .
```

**CG-1 Domain**

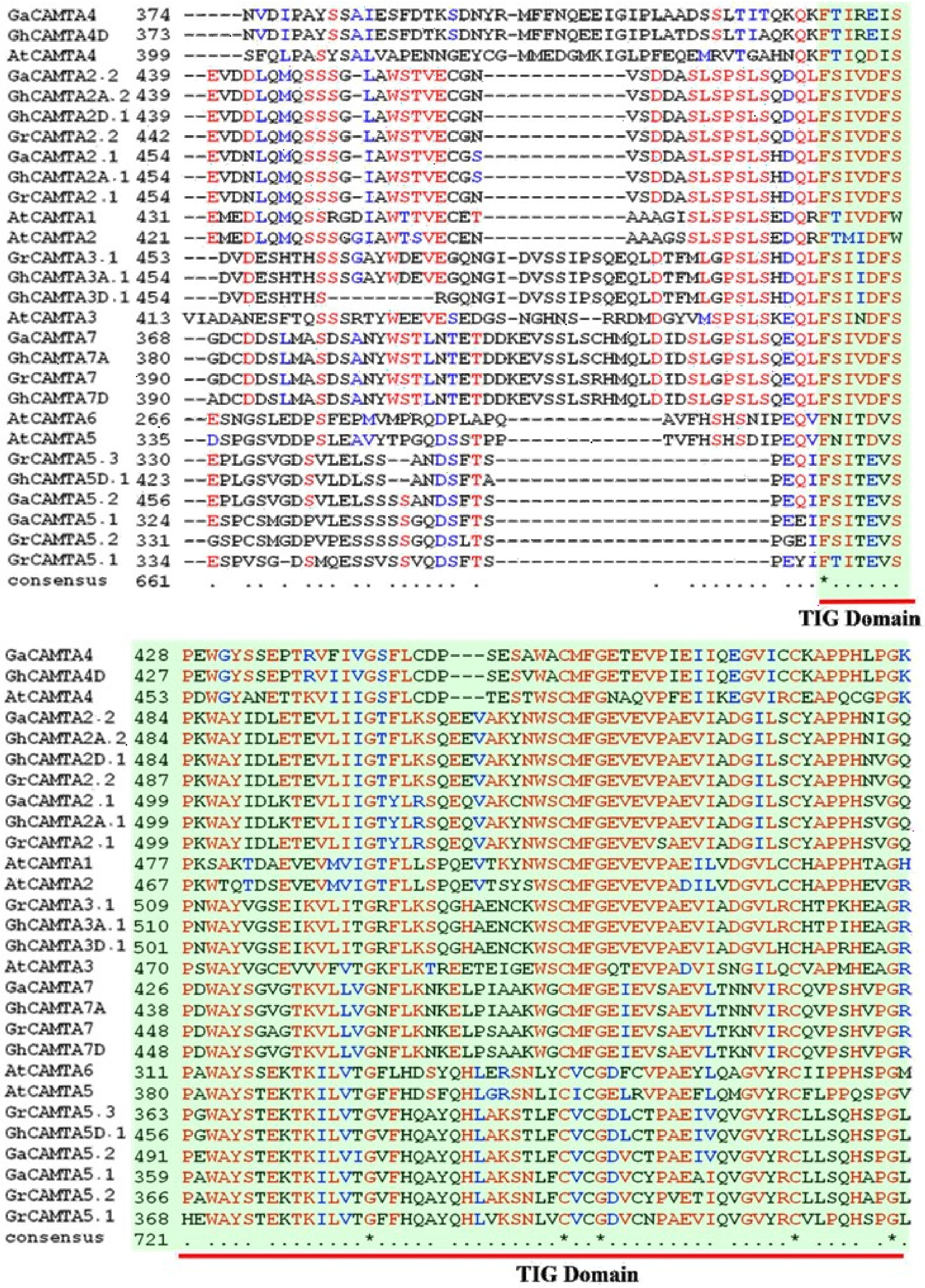
TIG Domain
TIG Domain

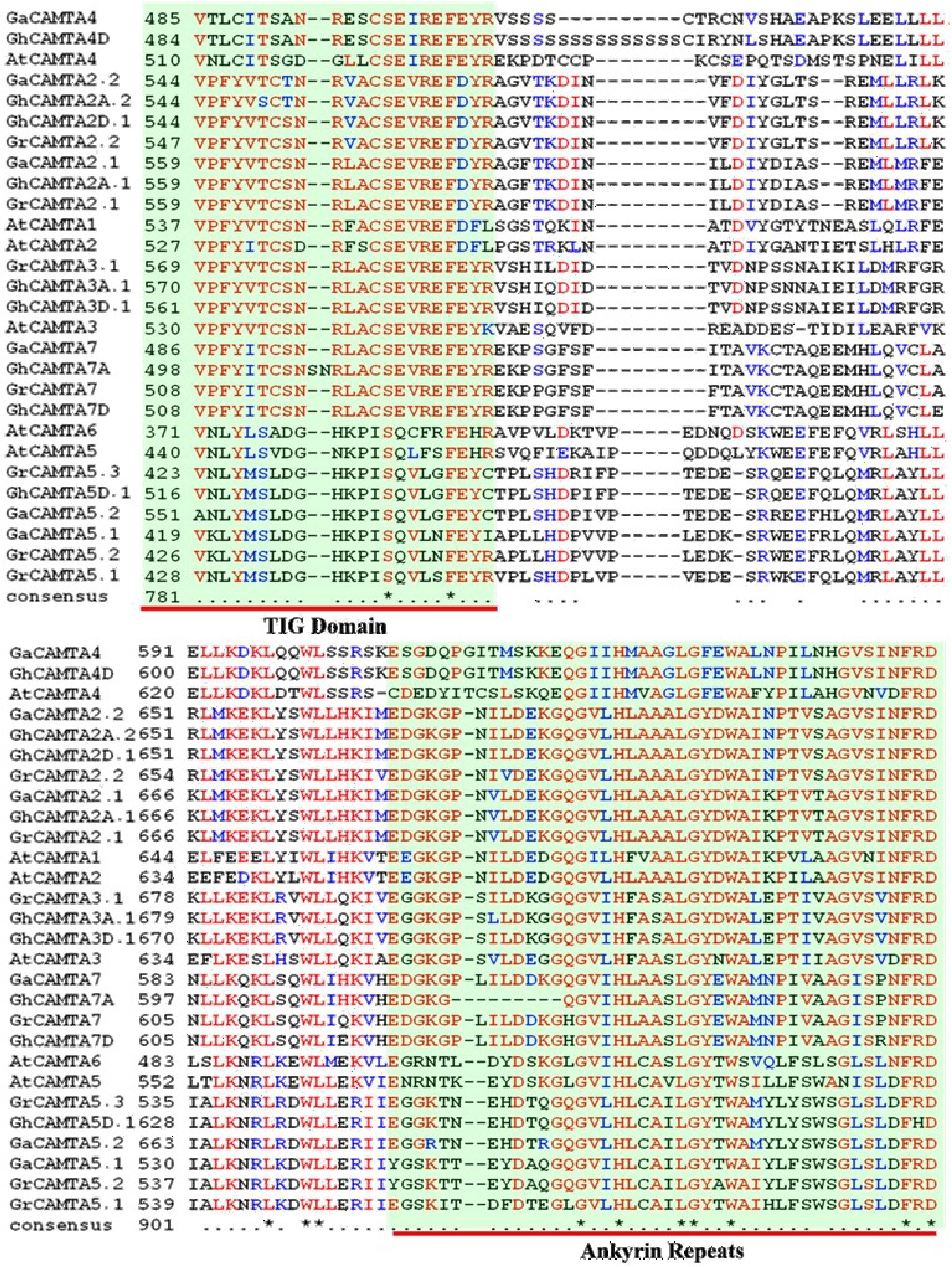
GaCAMTA4 485 VTLCITSAN--RESCSEIREFEYRVSSSS----------CTRCNVSHAEAPKSLEELLLL
GhCAMTA4D 484 VTLCITSAN--RESCSEIREFEYRVSSSSSSSSSSSSSCIRYNLSHAEAPKSLEELLLL
AtCAMTA4 510 VNLCITSGD--GLLCSEIREFEYREKPDTCCP--------KCSEPQTSDMSTSPNELILL
GaCAMTA2.2 544 VPFYVTCTN--RVACSEVREFDYRAGVTKDIN---------VFDIYGLTS--REMLLRLK
GhCAMTA2A.2 544 VPFYVSCTN--RVACSEVREFDYRAGVTKDIN---------VFDIYGLTS--REMLLRLK
GhCAMTA2D.1 544 VPFYVTCSN--RVACSEVREFDYRAGVTKDIN---------VFDIYGLTS--REMLLRLK
GrCAMTA2.2 547 VPFYVTCSN--RVACSEVREFDYRAGVTKDIN---------VFDIYGLTS--REMLLRLK
GaCAMTA2.1 559 VPFYVTCSN--RLACSEVREFDYRAGFTKDIN---------ILDIYDIAS--REMLMRFE
GhCAMTA2A.1 559 VPFYVTCSN--RLACSEVREFDYRAGFTKDIN---------ILDIYDIAS--REMLMRFE
GrCAMTA2.1 559 VPFYVTCSN--RLACSEVREFDYRAGFTKDIN---------ILDIYDIAS--REMLMRFE
AtCAMTA1 537 VPFYVTCSN--RFACSEVREFDFLSGSTQKIN---------ATDVYGTYTNEASLQLRFE
AtCAMTA2 527 VPFYITCSD--RFSCSEVREFDFLPGSTRKLN---------ATDIYGANTIETSLHLRFE
GrCAMTA3.1 569 VPFYVTCSN--RLACSEVREFEYRVSHILDID---------TVDNPSSNAIKILDMRFGR
GhCAMTA3A.1 570 VPFYVTCSN--RLACSEVREFEYRVSHIQDID---------TVDNPSNNAIEILDMRFGR
GhCAMTA3D.1 561 VPFYVTCSN--RLACSEVREFEYRVSHIQDID---------TVDNPSSNAIEILDMRFGR
AtCAMTA3 530 VPFYVTCSN--RLACSEVREFEYKVAESQVFD---------READDES-TIDILEARFVK
GaCAMTA7 486 VPFYITCSN--RLACSEVREFEYREKPSGFSF---------ITAVKCTAQEEMHLQVCLA
GhCAMTA7A 498 VPFYITCSNSNRLACSEVREFEYREKPSGFSF---------ITAVKCTAQEEMHLQVCLA
GrCAMTA7 508 VPFYITCSN--RLACSEVREFEYREKPPGFSF---------FTAVKCTAQEEMHLQVCLA
GhCAMTA7D 508 VPFYITCSN--RLACSEVREFEYREKPPGFSF---------FTAVKCTAQEEMHLQVCLE
AtCAMTA6 371 VNLYLSADG--HKPISQCFRFEHRAVPVLDKTVP-----EDNQDSKWEEFEFQVRLSHLL
AtCAMTA5 440 VNLYLSVDG--NKPISQLFSFEHRSVQFIEKAIP-----QDDQLYKWEEFEFQVRLAHLL
GrCAMTA5.3 423 VNLYMSLDG--HKPISQVLGFEYCTPLSHDRIFP-----TEDE-SRQEEFQLQMRLAYLL
GhCAMTA5D.1 516 VNLYMSLDG--HKPISQVLGFEYCTPLSHDPIFP-----TEDE-SRQEEFQLQMRLAYLL
GaCAMTA5.2 551 ANLYMSLDG--HKPISQVLGFEYCTPLSHDPIVP-----TEDE-SRREEFHLQMRLAYLL
GaCAMTA5.1 419 VKLYMSLDG--HKPISQVLNFEYIAPLLHDPVVP-----LEDK-SRWEEFRLQMRLAYLL
GrCAMTA5.2 426 VKLYMSLDG--HKPISQVLNFEYRAPLLHDPVVP-----LEDK-SRWEEFRLQMRLAYLL
GrCAMTA5.1 428 VNLYMSLDG--HKPISQVLSFEYRVPLSHDPLVP-----VEDE-SRWKEFQLQMRLAYLL
consensus 781
TIG Domain
GaCAMTA4 591 ELLKDKLQQWLSSRSKESGDQPGITMSKKEQGIIHMAAGLGFEWALNPILNHGVSINFRD
GhCAMTA4D 600 ELLKDKLQQWLSSRSKESGDQPGITMSKKEQGIIHMAAGLGFEWALNPILNHGVSINFRD
AtCAMTA4 620 ELLKDKLDTWLSSRS-CDEDYITCSLSKQEQGIIHMVAGLGFEWAFYPILAHGVNVDFRD
GaCAMTA2.2 651 RLMKEKLYSWLLHKIMEDGKGP-NILDEKGQGVLHLAAALGYDWAINPTVSAGVSINFRD
GhCAMTA2A.2 651 RLMKEKLYSWLLHKIMEDGKGP-NILDEKGQGVLHLAAALGYDWAINPTVSAGVSINFRD
GhCAMTA2D.1 651 RLMKEKLYSWLLHKIMEDGKGP-NILDEKGQGVLHLAAALGYDWAINPTVSAGVSINFRD
GrCAMTA2.2 654 RLMKEKLYSWLLHKIVEDGKGP-NIVDEKGQGVLHLAAALGYDWAINPTVSAGVSINFRD
GaCAMTA2.1 666 KLMKEKLYSWLLHKIMEDGKGP-NVLDEKGQGVLHLAAALGYDWAIKPTVTAGVSINFRD
GhCAMTA2A.1 666 KLMKEKLYSWLLHKIMEDGKGP-NVLDEKGQGVLHLAAALGYDWAIKPTVTAGVSINFRD
GrCAMTA2.1 666 KLMKEKLYSWLLHKIMEDGKGP-NVLDEKGQGVLHLAAALGYDWAIKPTVTAGVSINFRD
AtCAMTA1 644 ELFEEELYIWLIHKVTEEGKGP-NILDEDGQGILHFVAALGYDWAIKPVLAAGVNINFRD
AtCAMTA2 634 EEFEDKLYLWLIHKVTEEGKGP-NILDEDGQGVLHLAAALGYDWAIKPILAAGVSINFRD
GrCAMTA3.1 678 KLLKEKLRVWLLQKIVEGGKGP-SILDKGGQGVIHFASALGYDWALEPTIVAGVSVNFRD
GhCAMTA3A.1 679 KLLKEKLRVWLLQKIVEGGKGP-SLLDKGGQGVIHFASALGYDWALEPTIVAGVSVNFRD
GhCAMTA3D.1 670 KLLKEKLRVWLLQKIVEGGKGP-SILDKGGQGVIHFASALGYDWALEPTIVAGVSVNFRD
AtCAMTA3 634 EFLKESLHSWLLQKIAEGGKGP-SVLDEGGQGVLHFAASLGYNWALEPTIIAGVSVDFRD
GaCAMTA7 583 NLLKQKLSQWLIHKVHEDGKGP-LILDDKGQGVIHLAASLGYEWAMNPIVAAGISPNFRD
GhCAMTA7A 597 NLLKQKLSQWLIHKVHEDGKG---------QGVIHLAASLGYEWAMNPIVAAGISPNFRD
GrCAMTA7 605 NLLKQKLSQWLIQKVHEDGKGP-LILDDKGHGVIHLAASLGYEWAMNPIVAAGISPNFRD
GhCAMTA7D 605 NLLKQKLSQWLIEKVHEDGKGP-LILDDKGHGVIHLAASLGYEWAMNPIVAAGISRNFRD
AtCAMTA6 483 LSLKNRLKEWLMEKVLEGRNTL--DYDSKGLGVIHLCASLGYTWSVQLFSLSGLSLNFRD
AtCAMTA5 552 LTLKNRLKEWLLEKVIENRNTK--EYDSKGLGVIHLCAVLGYTWSILLFSWANISLDFRD
GrCAMTA5.3 535 IALKNRLRDWLLERIIEGGKTN--EHDTQGQGVLHLCAILGYTWAMYLYSWSGLSLDFRD
GhCAMTA5D.1 628 IALKNRLRDWLLERIIEGGKTN--EHDTQGQGVLHLCAILGYTWAMYLYSWSGLSLDFHD
GaCAMTA5.2 663 IALKNRLRDWLLERIIEGGRTN--EHDTRGQGVLHLCAILGYTWAMYLYSWSGLSLDFRD
GaCAMTA5.1 530 IALKNRLKDWLLERIIYGSKTT--EYDAQGQGVIHLCAILGYTWAIYLFSWSGLSLDFRD
GrCAMTA5.2 537 IALKNRLKDWLLERIIYGSKTT--EYDAQGQGVIHLCAILGYAWAIYLFSWSGLSLDFRD
GrCAMTA5.1 539 IALKNRLKDWLLERIIEGSKIT--DFDTEGLGVLHLCAILGYTWAIHLFSWSGLSLDFRD
consensus 901
Ankyrin Repeats

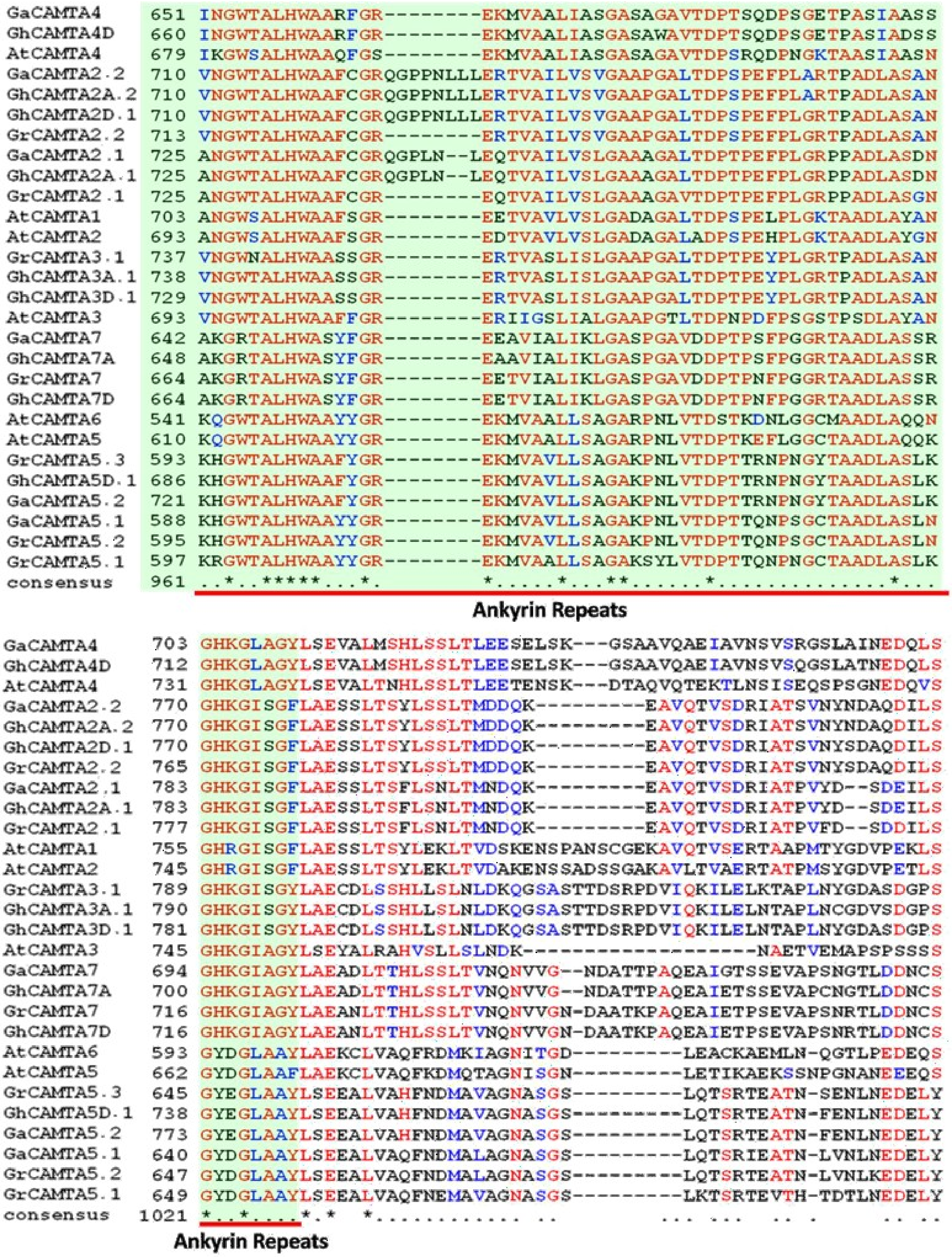

GaCAMTA4 651 INGWTALHWAARFGR--------EKMVAALIASGASAGAVTDPTSQDPSGETPASIAASS
GhCAMTA4D 660 INGWTALHWAARFGR--------EKMVAALIASGASAWAVTDPTSQDPSGETPASIADSS
AtCAMTA4 679 IKGWSALHWAAQFGS--------EKMVAALIASGASAGAVTDPSRQDPNGKTAASIAASN
GaCAMTA2.2 710 VNGWTALHWAAFCGRQGPPNLLLERTVAILVSVGAAPGALTDPSPEFPLARTPADLASAN
GhCAMTA2A.2 710 VNGWTALHWAAFCGRQGPPNLLLERTVAILVSVGAAPGALTDPSPEFPLARTPADLASAN
GhCAMTA2D.1 710 VNGWTALHWAAFCGRQGPPNLLLERTVAILVSVGAAPGALTDPSPEFPLGRTPADLASAN
GrCAMTA2.2 713 VNGWTALHWAAFCGR--------ERTVAILVSVGAAPGALTDPSPEFPLGRTPADLASAN
GaCAMTA2.1 725 ANGWTALHWAAFCGRQGPLN--LEQTVAILVSLGAAAGALTDPTPEFPLGRPPADLASDN
GhCAMTA2A.1 725 ANGWTALHWAAFCGRQGPLN--LEQTVAILVSLGAAAGALTDPTPEFPLGRPPADLASDN
GrCAMTA2.1 725 ANGWTALHWAAFCGR--------EQTVAILVSLGAAAGAVTDPTPEFPLGRPPADLASGN
AtCAMTA1 703 ANGWSALHWAAFSGR--------EETVAVLVSLGADAGALTDPSPELPLGKTAADLAYAN
AtCAMTA2 693 ANGWSALHWAAFSGR--------EDTVAVLVSLGADAGALADPSPEHPLGKTAADLAYGN
GrCAMTA3.1 737 VNGWNALHWAASSGR--------ERTVASLISLGAAPGALTDPTPEYPLGRTPADLASAN
GhCAMTA3A.1 738 VNGWTALHWAASSGR--------ERTVASLISLGAAPGALTDPTPEYPLGRTPADLASAN
GhCAMTA3D.1 729 VNGWTALHWAASSGR--------ERTVASLISLGAAPGALTDPTPEYPLGRTPADLASAN
AtCAMTA3 693 VNGWTALHWAAFFGR--------ERIIGSLIALGAAPGTLTDPNPDFPSGSTPSDLAYAN
GaCAMTA7 642 AKGRTALHWASYFGR--------EEAVIALIKLGASPGAVDDPTPSFPGGRTAADLASSR
GhCAMTA7A 648 AKGRTALHWASYFGR--------EAAVIALIKLGASPGAVDDPTPSFPGGRTAADLASSR
GrCAMTA7 664 AKGRTALHWASYFGR--------EETVIALIKLGASPGAVDDPTPNFPGGRTAADLASSR
GhCAMTA7D 664 AKGRTALHWASYFGR--------EETVIALIKLGASPGAVDDPTPNFPGGRTAADLASSR
AtCAMTA6 541 KQGWTALHWAAYYGR--------EKMVAALLSAGARPNLVTDSTKDNLGGCMAADLAQQN
AtCAMTA5 610 KQGWTALHWAAYYGR--------EKMVAALLSAGARPNLVTDPTKEFLGGCTAADLAQQK
GrCAMTA5.3 593 KHGWTALHWAAFYGR--------EKMVAVLLSAGAKPNLVTDPTTRNPNGYTAADLASLK
GhCAMTA5D.1 686 KHGWTALHWAAFYGR--------EKMVAVLLSAGAKPNLVTDPTTRNPNGYTAADLASLK
GaCAMTA5.2 721 KHGWTALHWAAFYGR--------EKMVAVLLSAGAKPNLVTDPTTRNPNGYTAADLASLK
GaCAMTA5.1 588 KHGWTALHWAAYYGR--------EKMVAVLLSAGAKPNLVTDPTTQNPSGCTAADLASLN
GrCAMTA5.2 595 KHGWTALHWAAYYGR--------EKMVAVLLSAGAKPNLVTDPTTQNPSGCTAADLASLN
GrCAMTA5.1 597 KRGWTALHWAAYYGR--------EKMVAALLSAGAKSYLVTDPTTQNPNGCTAADLASLK
consensus 961 ..*..*****...*. *.....*...**......*..............*...
Ankyrin Repeats
GaCAMTA4 703 GHKGLAGYLSEVALMSHLSSLTLEESELSK---GSAAVQAEIAVNSVSRGSLAINEDQLS
GhCAMTA4D 712 GHKGLAGYLSEVALMSHLSSLTLEESELSK---GSAAVQAEIAVNSVSQGSLATNEDQLS
AtCAMTA4 731 GHKGLAGYLSEVALTNHLSSLTLEETENSK---DTAQVQTEKTLNSISEQSPSGNEDQVS
GaCAMTA2.2 770 GHKGISGFLAESSLTSYLSSLTMDDQK---------EAVQTVSDRIATSVNYNDAQDILS
GhCAMTA2A.2 770 GHKGISGFLAESSLTSYLSSLTMDDQK---------EAVQTVSDRIATSVNYNDAQDILS
GhCAMTA2D.1 770 GHKGISGFLAESSLTSYLSSLTMDDQK---------EAVQTVSDRIATSVNYSDAQDILS
GrCAMTA2.2 765 GHKGISGFLAESSLTSYLSSLTMDDQK---------EAVQTVSDRIATSVNYSDAQDILS
GaCAMTA2.1 783 GHKGISGFLAESSLTSFLSNLTMNDQK---------EAVQTVSDRIATPVYD--SDEILS
GhCAMTA2A.1 783 GHKGISGFLAESSLTSFLSNLTMNDQK---------EAVQTVSDRIATPVYD--SDEILS
GrCAMTA2.1 777 GHKGISGFLAESSLTSFLSNLTMNDQK---------EAVQTVSDRIATPVFD--SDDILS
AtCAMTA1 755 GHRGISGFLAESSLTSYLEKLTVDSKENSPANSCGEKAVQTVSERTAAPMTYGDVPEKLS
AtCAMTA2 745 GHRGISGFLAESSLTSYLEKLTVDAKENSSADSSGAKAVLTVAERTATPMSYGDVPETLS
GrCAMTA3.1 789 GHKGISGYLAECDLSSHLLSLNLDKQGSASTTDSRPDVIQKILELKTAPLNYGDASDGPS
GhCAMTA3A.1 790 GHKGISGYLAECDLSSHLLSLNLDKQGSASTTDSRPDVIQKILELNTAPLNCGDVSDGPS
GhCAMTA3D.1 781 GHKGISGYLAECDLSSHLLSLNLDKQGSASTTDSRPDVIQKILELNTAPLNYGDASDGPS
AtCAMTA3 745 GHKGIAGYLSEYALRAHVSLLSLNDK------------------NAETVEMAPSPSSS
GaCAMTA7 694 GHKGIAGYLAEADLTTHLSSLTVNQNVVG--NDATTPAQEAIGTSSEVAPSNGTLDDNCS
GhCAMTA7A 700 GHKGIAGYLAEADLTTHLSSLTVNQNVVG--NDATTPAQEAIETSSEVAPCNGTLDDNCS
GrCAMTA7 716 GHKGIAGYLAEANLTTHLSSLTVNQNVVGN-DAATKPAQEAIETPSEVAPSNRTLDDNCS
GhCAMTA7D 716 GHKGIAGYLAEANLTTHLSSLTVNQNVVGN-DAATKPAQEAIETPSEVAPSNRTLDDNCS
AtCAMTA6 593 GYDGLAAYLAEKCLVAQFRDMKIAGNITGD---------LEACKAEMLN-QGTLPEDEQS
AtCAMTA5 662 GYDGLAAFLAEKCLVAQFKDMQTAGNISGN---------LETIKAEKSSNPGNANEEEQS
GrCAMTA5.3 645 GYEGLAAYLSEEALVAHFNDMAVAGNASGS---------LQTSRTEATN-SENLNEDELY
GhCAMTA5D.1 738 GYEGLAAYLSEEALVAHFNDMAVAGNASGS---------LQTSRTEATN-FENLNEDELY
GaCAMTA5.2 773 GYEGLAAYLSEEALVAHFNDMAVAGNASGS---------LQTSRTEATN-FENLNEDELY
GaCAMTA5.1 640 GYDGLAAYLSEEALVAQFNDMALAGNASGS---------LQTSRIEATN-LVNLNEDELY
GrCAMTA5.2 647 GYDGLAAYLSEEALVAQFNDMALAGNASGS---------LQTSRTEATN-LVNLKEDELY
GrCAMTA5.1 649 GYDGLAAYLSEEALVAQFNEMAVAGNASGS---------LKTSRTEVTH-TDTLNEDELY
consensus 1021 *..*.....*.* *............ .. ... ... .. . .. ..
Ankyrin Repeats

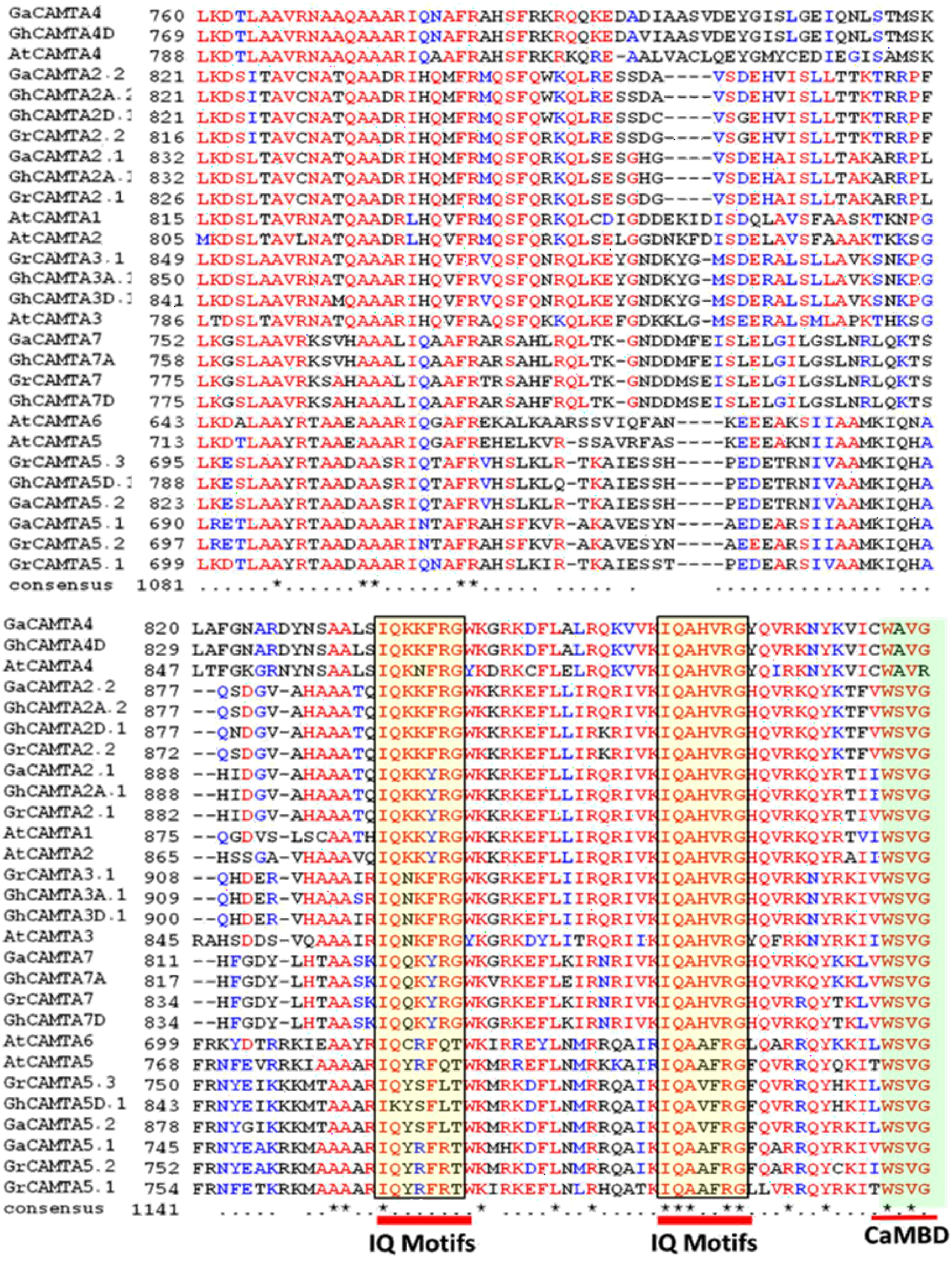
IQ Motifs
IQ Motifs
CaMBD

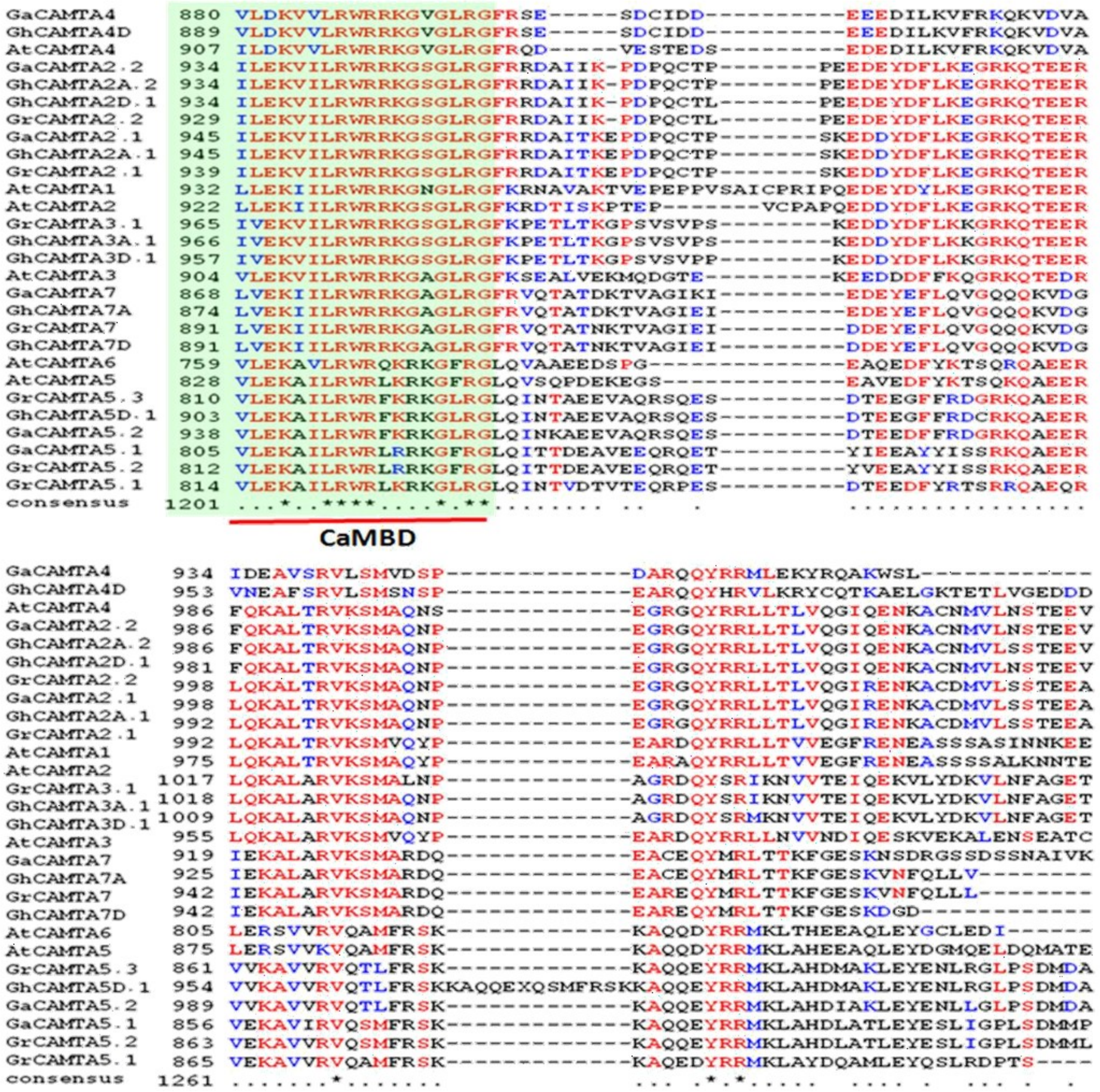


**Supplementary Fig. S1 Alignment of cotton CAMTA proteins with Arabidopsis CAMTAs.** Multiple sequence alignment of 22 *Gossypium* putative CAMTA proteins and six *Arabidopsis thaliana* CAMTA proteins obtained by Clustal X. Green colored shading indicates conserved domains (CG-1, a TIG domain, ankyrin repeats, IQ motifs, and a CaMBD) of a typical CAMTA protein. Red letters with asterisk represent the amino acid conservation in all domains.

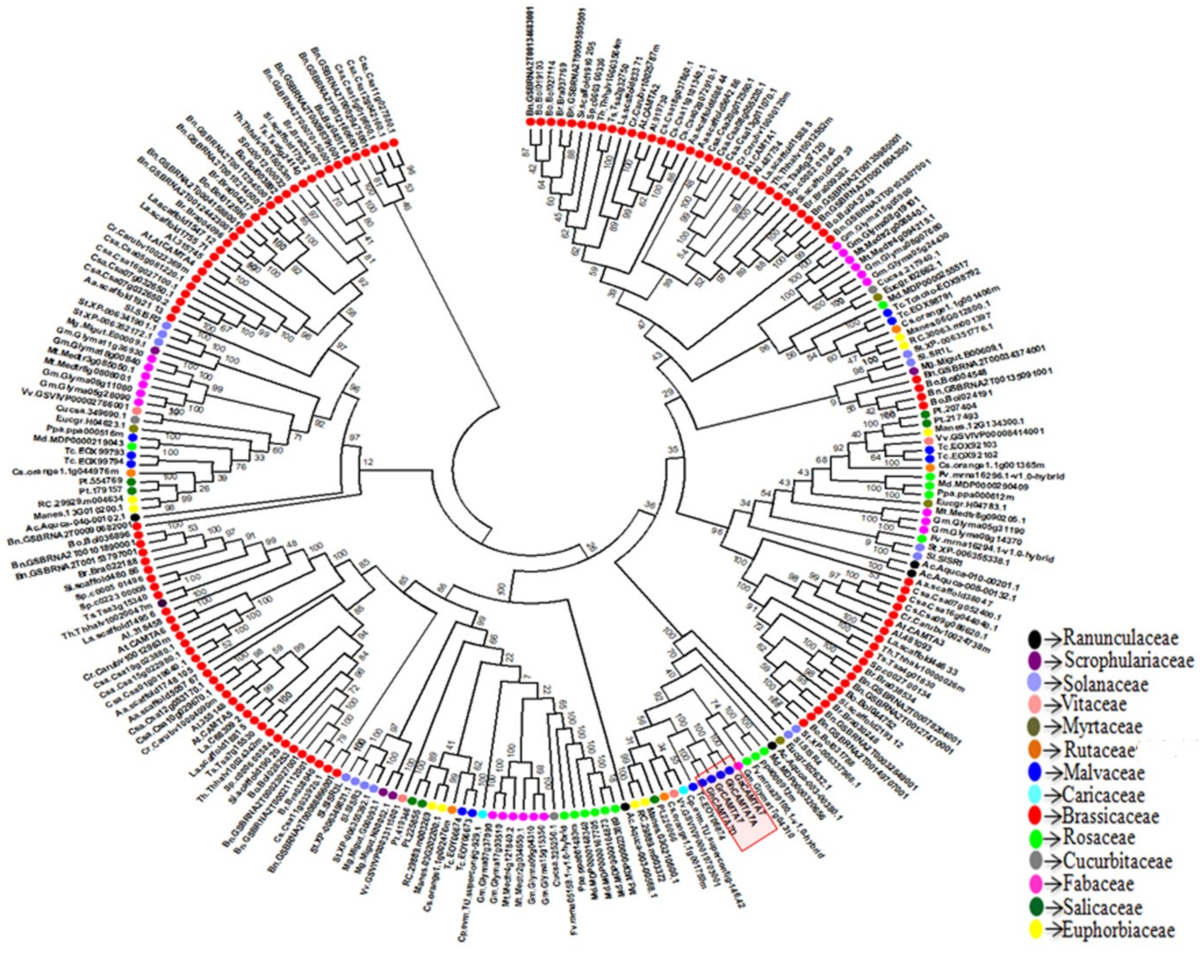


**Supplementary Fig. S2 Phylogenetic tree of Group II cotton CAMTAs with different eudicots.** CAMTA proteins of group II (GaCAMTA7, GrCAMTA7, GhCAMTA7A, and GhCAMTA7D) and different eudicots were align with each other. The unrooted phylogenetic tree was constructed using 500 bootstrap values. Different colors of dots represented the different plant families.

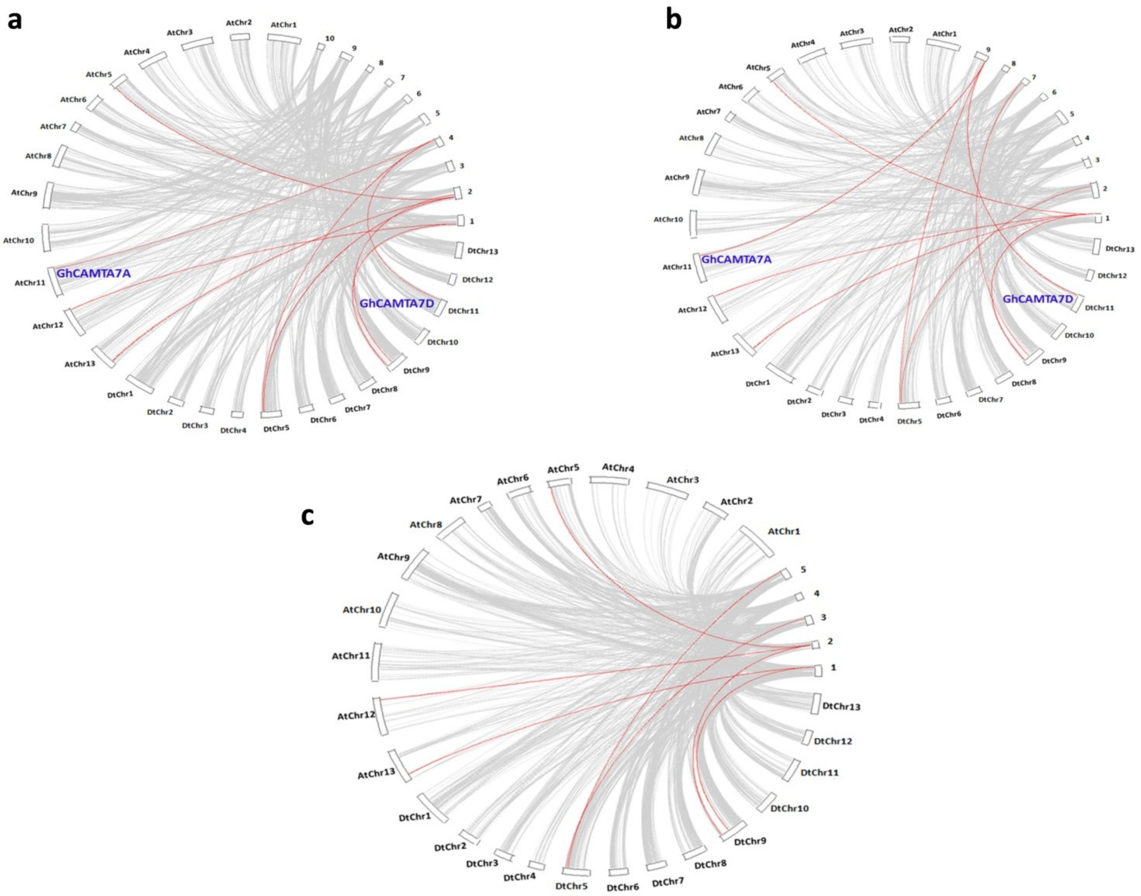

a
AtChr1
AtChr2
AtChr3
AtChr4
AtChr5
AtChr6
AtChr7
AtChr8
AtChr9
AtChr10
AtChr11
AtChr12
AtChr13
10
9
8
7
6
5
4
3
2
1
DtChr1
DtChr2
DtChr3
DtChr4
DtChr5
DtChr6
DtChr7
DtChr8
DtChr9
DtChr10
DtChr11
DtChr12
DtChr13
GhCAMTA7A
GhCAMTA7D
b
AtChr1
AtChr2
AtChr3
AtChr4
AtChr5
AtChr6
AtChr7
AtChr8
AtChr9
AtChr10
AtChr11
AtChr12
AtChr13
9
8
7
6
5
4
3
2
1
DtChr1
DtChr2
DtChr3
DtChr4
DtChr5
DtChr6
DtChr7
DtChr8
DtChr9
DtChr10
DtChr11
DtChr12
DtChr13
GhCAMTA7A
GhCAMTA7D
c
AtChr1
AtChr2
AtChr3
AtChr4
AtChr5
AtChr6
AtChr7
AtChr8
AtChr9
AtChr10
AtChr11
AtChr12
AtChr13
5
4
3
2
1
DtChr1
DtChr2
DtChr3
DtChr4
DtChr5
DtChr6
DtChr7
DtChr8
DtChr9
DtChr10
DtChr11
DtChr12
DtChr13

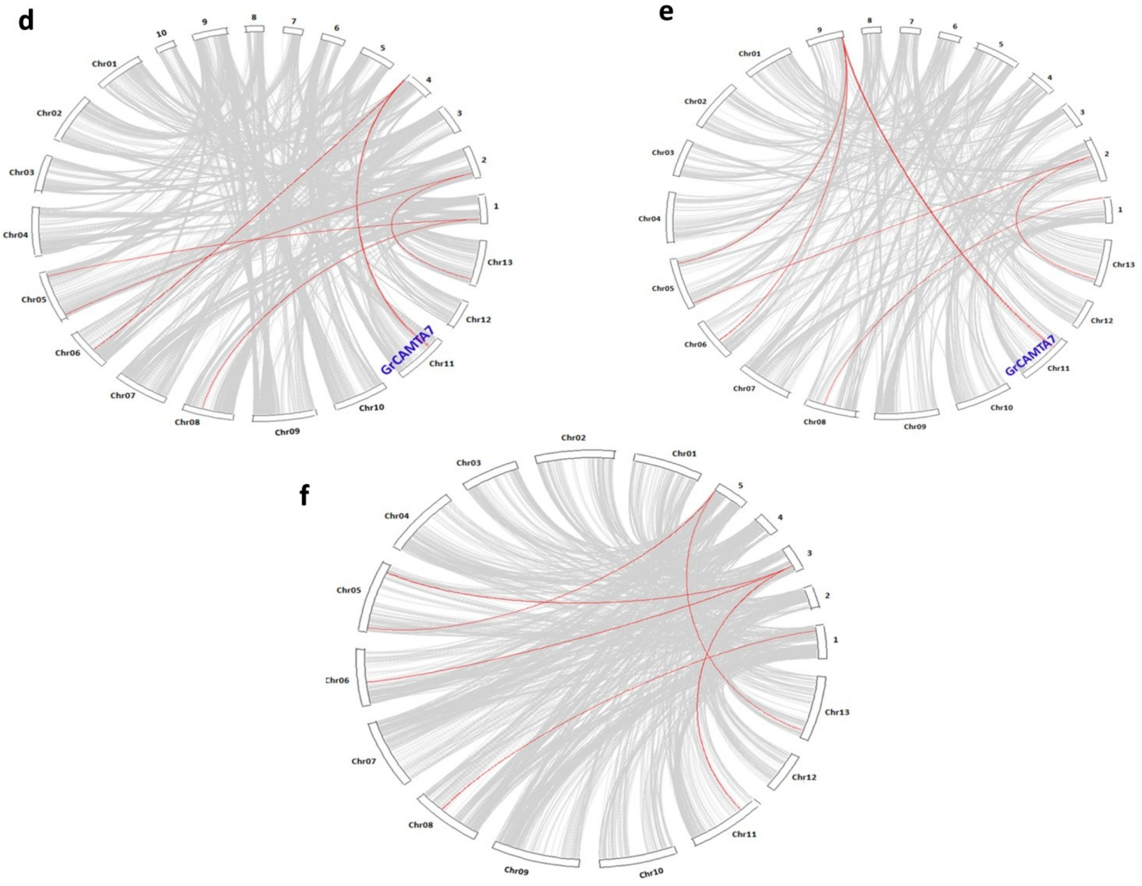
d
e
f
GrCAMTA7
GrCAMTA7
Chr01
Chr02
Chr03
Chr04
Chr05
Chr06
Chr07
Chr08
Chr09
Chr10
Chr11
Chr12
Chr13

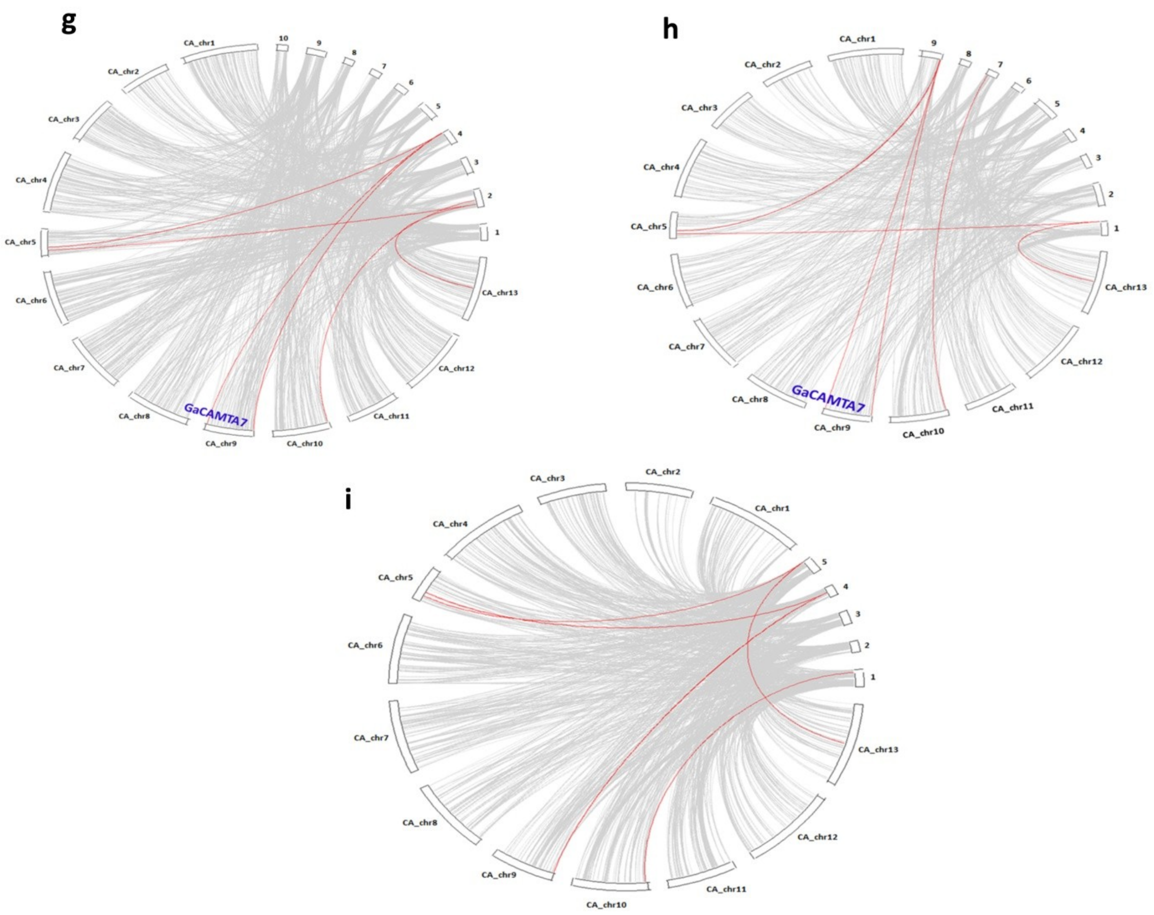


**Supplementary Fig. S3 Synteny analysis of cotton CAMTA genes with *Theobroma cacao* (Malvaceae), *Citrus sinensis* (Rutaceae), and *Arabidopsis thaliana* (Brassicaceae) genomes.** A schematic representation of the orthologs identified between (a) *G. hirsutum* (At1 to At13 and Dt1 to Dt13) vs. *T. cacao* (1 to 10), (b) *G. hirsutum* vs. *C. sinensis* (1 to 9) and (c) *G. hirsutum* vs. *A. thaliana* (1 to 5) chromosomes (d) *G. raimondii* (Chr01 to Chr13) vs. *T. cacao,* (e) *G. raimondii* vs. *C. sinensis* and (f) *G. raimondii* vs. *A. thaliana* (g) *G. arboreum* (CA_chr01 to CA_chr13) vs. *T. cacao*, (h) *G. arboreum* vs. *C. sinensis* and (i) *G. arboreum* vs. *A. thaliana* chromosomes. Dark red line lines indicate duplicate CAMTA genes and grey lines indicate collinear blocks within the genome of *G. hirsutum, G. raimondii, G. arboreum, Theobroma cacao*, *Citrus sinensis*, and *Arabidopsis thaliana.*

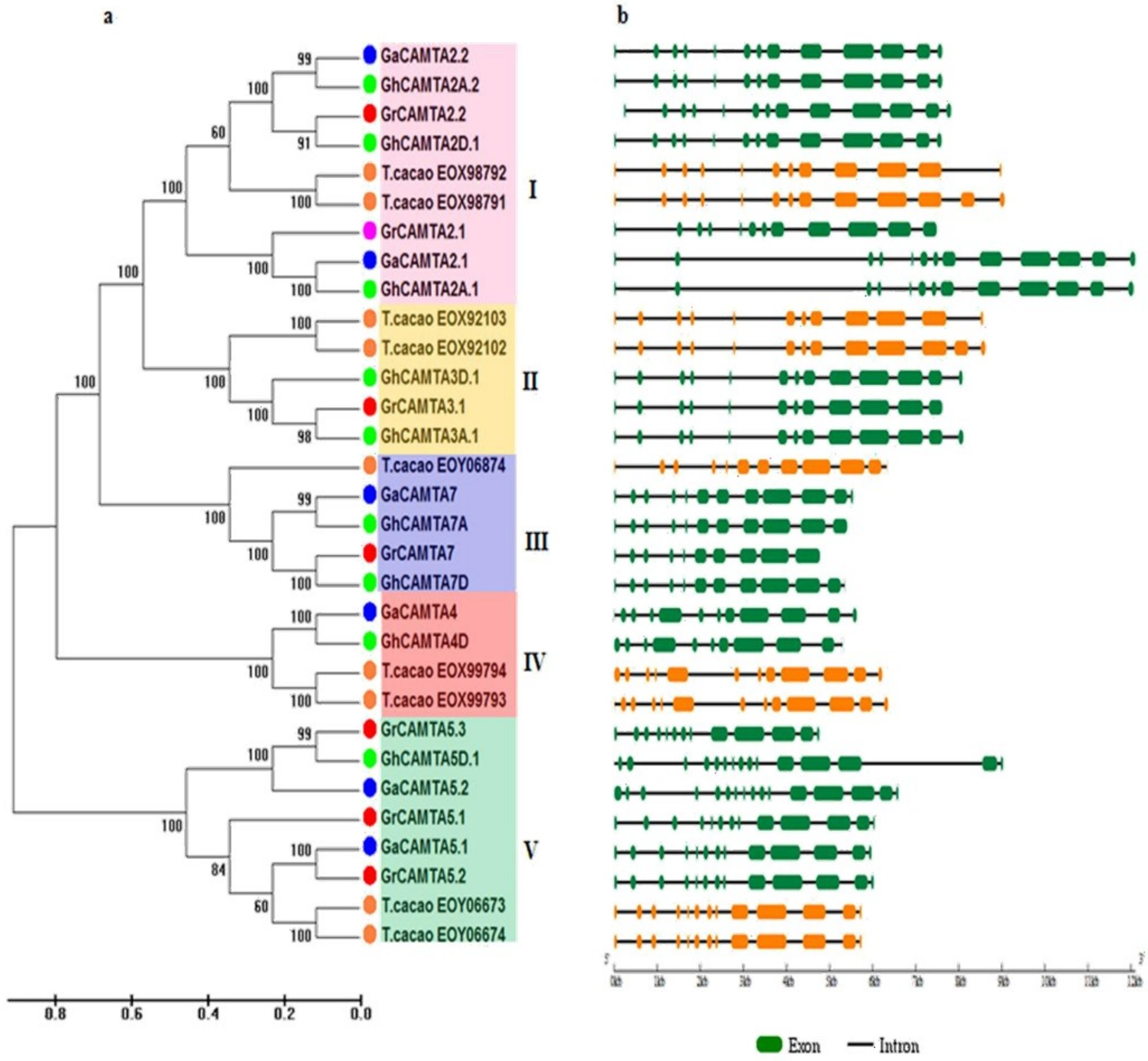


**Supplementary Fig. S4 Phylogenetic relationship between *Theobroma cacao* and *Gossypium* species.** (a) Phylogenetic tree of *G. arboreum*, *G. raimondii*, *G. hirsutum* and *T. cacao* CAMTAs constructed with ML method and using 1000 bootstrap values. Different colors of dots represented the different species of *Gossypium* (Blue, *G. arboreum*; Red, *G. raimondii*; Green, *G. hirsutum* and Orange, *T. cacao)*. Subgroup -I, -II, -III, -IV and -V colored in pink, yellow, purple, red, and green respectively. (b) Schematic diagram for the exon - intron organization of cotton and cacao CAMTA genes. The green boxes and black lines indicate the exons and introns, respectively.

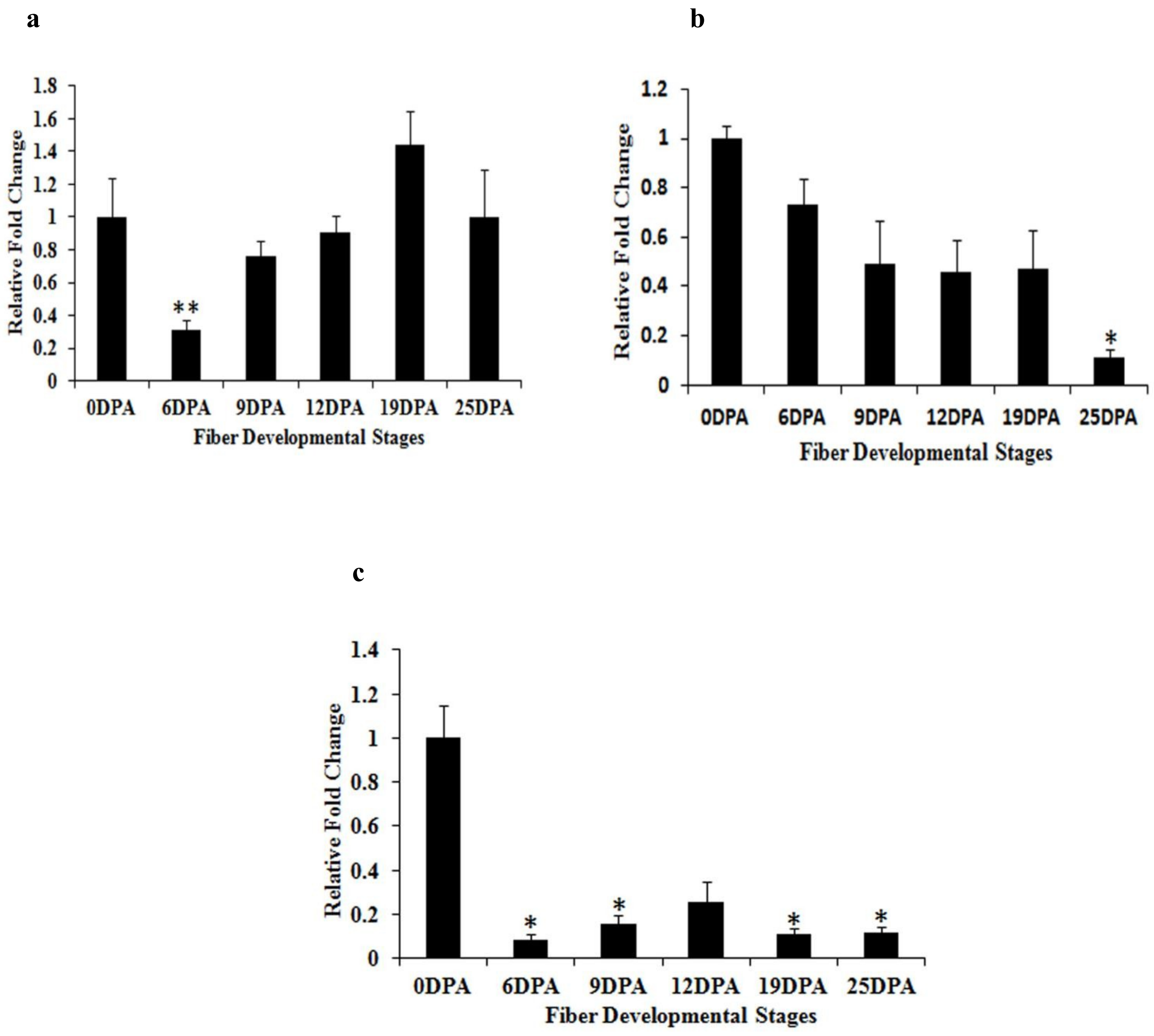


**Supplementary Fig. S5 Expression profiles of highly expressing CAMTAs (GhCAMTA2A.2 and GhCAMTA7A) and the least expressing CAMTA (GhCAMTA3D.1) at different fiber developmental stages.** (a) Expression profile of GhCAMTA2A.2 (b) GhCAMTA7A and (c) GhCAMTA3D.1. The bars are representative of two independent experiments each with three replicates. Error bars correspond to the standard error for three individual replicates.

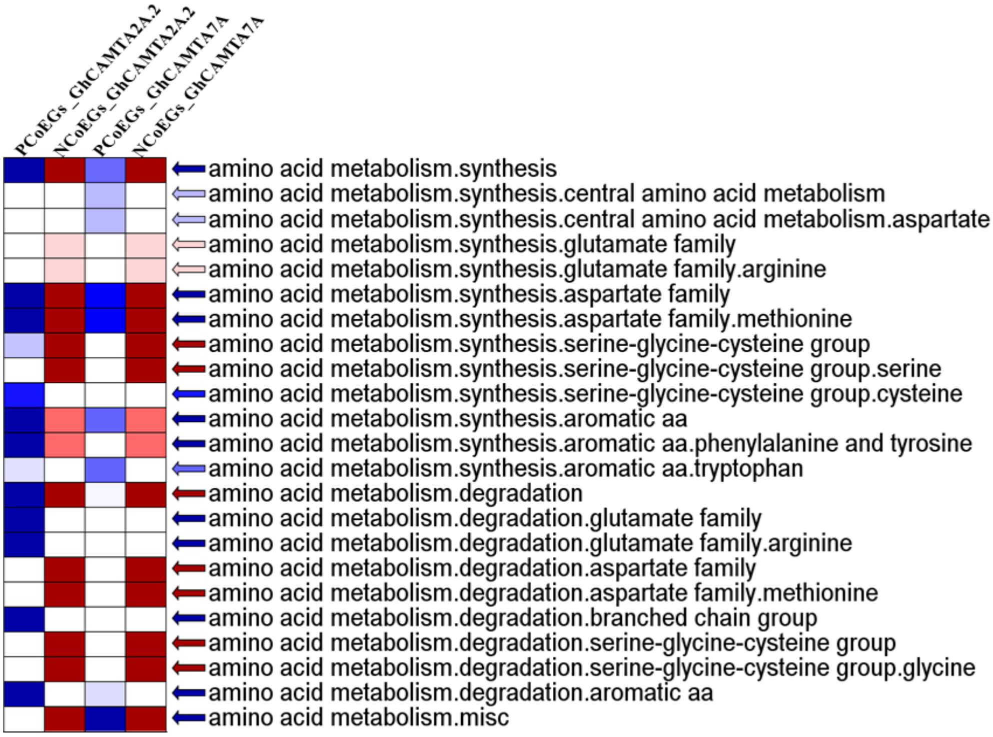
PCoEGs_GhCAMTA2A.2
NCoEGs_GhCAMTA2A.2
PCoEGs_GhCAMTA7A
NCoEGs_GhCAMTA7A
amino acid metabolism.synthesis
amino acid metabolism.synthesis.central amino acid metabolism
amino acid metabolism.synthesis.central amino acid metabolism.aspartate
amino acid metabolism.synthesis.glutamate family
amino acid metabolism.synthesis.glutamate family.arginine
amino acid metabolism.synthesis.aspartate family
amino acid metabolism.synthesis.aspartate family.methionine
amino acid metabolism.synthesis.serine-glycine-cysteine group
amino acid metabolism.synthesis.serine-glycine-cysteine group.serine
amino acid metabolism.synthesis.serine-glycine-cysteine group.cysteine
amino acid metabolism.synthesis.aromatic aa
amino acid metabolism.synthesis.aromatic aa.phenylalanine and tyrosine
amino acid metabolism.synthesis.aromatic aa.tryptophan
amino acid metabolism.degradation
amino acid metabolism.degradation.glutamate family
amino acid metabolism.degradation.glutamate family.arginine
amino acid metabolism.degradation.aspartate family
amino acid metabolism.degradation.aspartate family.methionine
amino acid metabolism.degradation.branched chain group
amino acid metabolism.degradation.serine-glycine-cysteine group
amino acid metabolism.degradation.serine-glycine-cysteine group.glycine
amino acid metabolism.degradation.aromatic aa
amino acid metabolism.misc

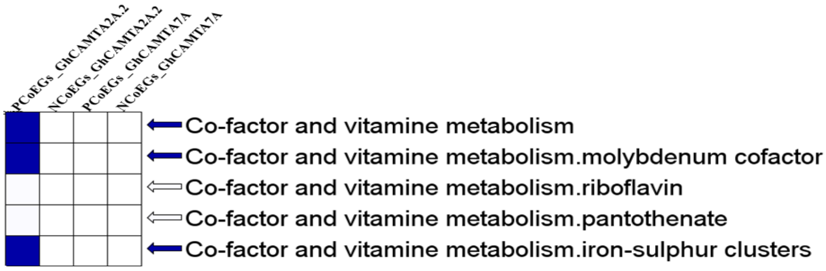
PCoEGs_GhCAMTA2A.2
NCoEGs_GhCAMTA2A.2
PCoEGs_GhCAMTA7A
NCoEGs_GhCAMTA7A
Co-factor and vitamine metabolism
Co-factor and vitamine metabolism.molybdenum cofactor
Co-factor and vitamine metabolism.riboflavin
Co-factor and vitamine metabolism.pantothenate
Co-factor and vitamine metabolism.iron-sulphur clusters

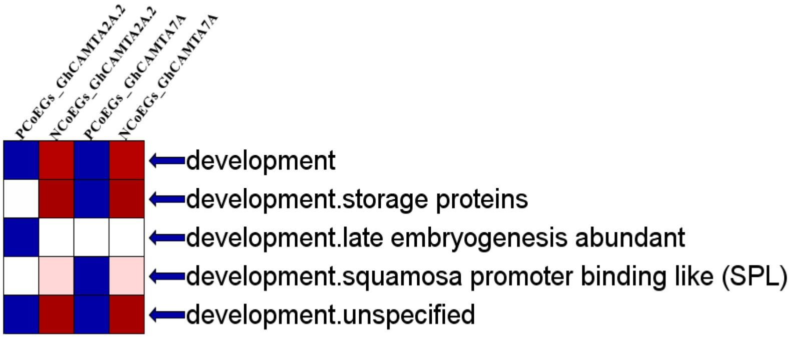
PCoEGs_GhCAMTA2A.2
NCoEGs_GhCAMTA2A.2
PCoEGs_GhCAMTA7A
NCoEGs_GhCAMTA7A
development
development.storage proteins
development.late embryogenesis abundant
development.squamosa promoter binding like (SPL)
development.unspecified

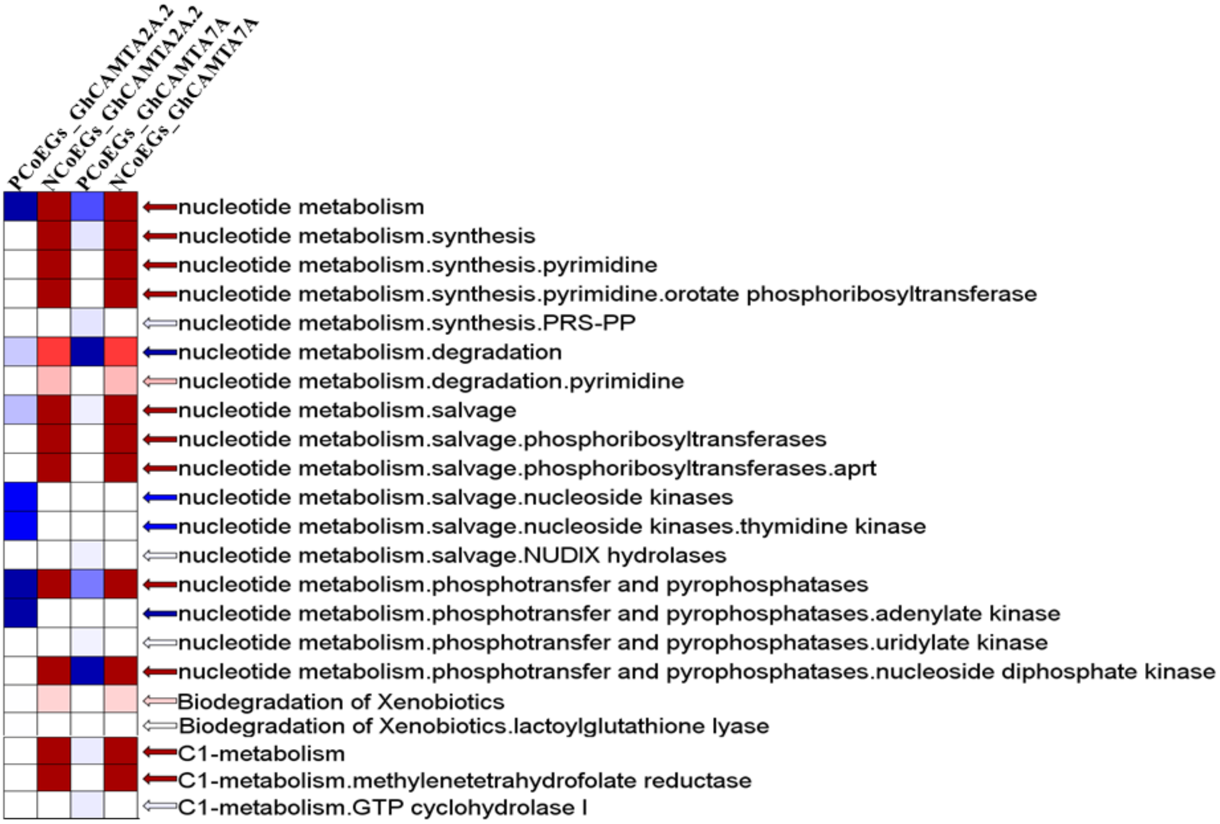
PCoEGs_GhCAMTA2A.2
NCoEGs_GhCAMTA2A.2
PCoEGs_GhCAMTA7A
NCoEGs_GhCAMTA7A
nucleotide metabolism
nucleotide metabolism.synthesis
nucleotide metabolism.synthesis.pyrimidine
nucleotide metabolism.synthesis.pyrimidine.orotate phosphoribosyltransferase
nucleotide metabolism.synthesis.PRS-PP
nucleotide metabolism.degradation
nucleotide metabolism.degradation.pyrimidine
nucleotide metabolism.salvage
nucleotide metabolism.salvage.phosphoribosyltransferases
nucleotide metabolism.salvage.phosphoribosyltransferases.aprt
nucleotide metabolism.salvage.nucleoside kinases
nucleotide metabolism.salvage.nucleoside kinases.thymidine kinase
nucleotide metabolism.salvage.NUDIX hydrolases
nucleotide metabolism.phosphotransfer and pyrophosphatases
nucleotide metabolism.phosphotransfer and pyrophosphatases.adenylate kinase
nucleotide metabolism.phosphotransfer and pyrophosphatases.uridylate kinase
nucleotide metabolism.phosphotransfer and pyrophosphatases.nucleoside diphosphate kinase
Biodegradation of Xenobiotics
Biodegradation of Xenobiotics.lactoylglutathione lyase
C1-metabolism
C1-metabolism.methylenetetrahydrofolate reductase
C1-metabolism.GTP cyclohydrolase I

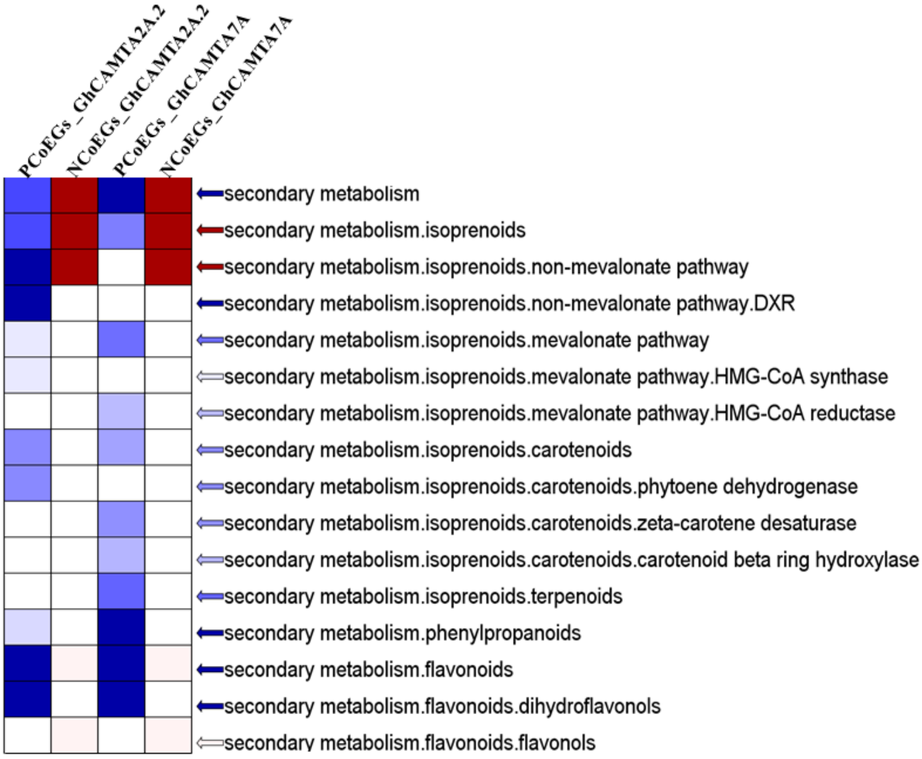
PCoEGs_GhCAMTA2A.2
NCoEGs_GhCAMTA2A.2
PCoEGs_GhCAMTA7A
NCoEGs_GhCAMTA7A
secondary metabolism
secondary metabolism.isoprenoids
secondary metabolism.isoprenoids.non-mevalonate pathway
secondary metabolism.isoprenoids.non-mevalonate pathway.DXR
secondary metabolism.isoprenoids.mevalonate pathway
secondary metabolism.isoprenoids.mevalonate pathway.HMG-CoA synthase
secondary metabolism.isoprenoids.mevalonate pathway.HMG-CoA reductase
secondary metabolism.isoprenoids.carotenoids
secondary metabolism.isoprenoids.carotenoids.phytoene dehydrogenase
secondary metabolism.isoprenoids.carotenoids.zeta-carotene desaturase
secondary metabolism.isoprenoids.carotenoids.carotenoid beta ring hydroxylase
secondary metabolism.isoprenoids.terpenoids
secondary metabolism.phenylpropanoids
secondary metabolism.flavonoids
secondary metabolism.flavonoids.dihydroflavonols
secondary metabolism.flavonoids.flavonols

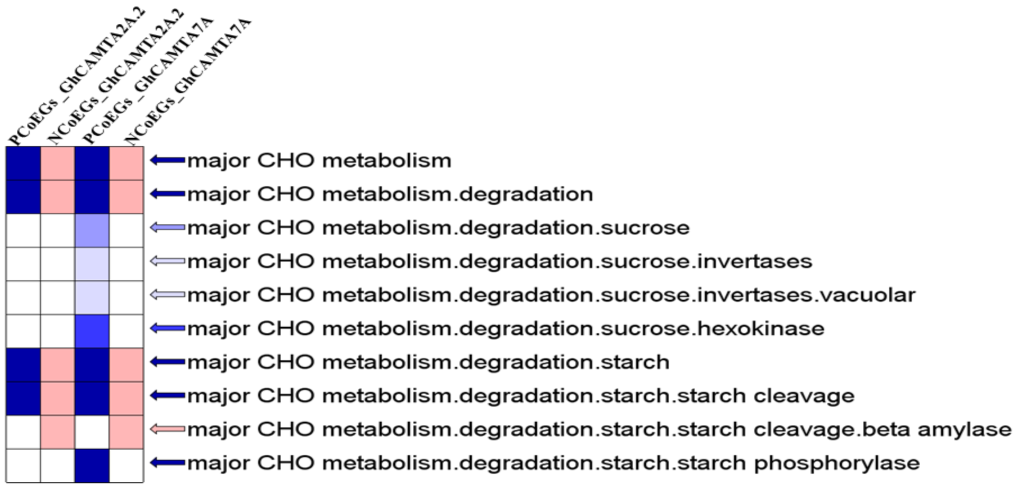
PCoEGs_GhCAMTA2A.2
NCoEGs_GhCAMTA2A.2
PCoEGs_GhCAMTA7A
NCoEGs_GhCAMTA7A
major CHO metabolism
major CHO metabolism.degradation
major CHO metabolism.degradation.sucrose
major CHO metabolism.degradation.sucrose.invertases
major CHO metabolism.degradation.sucrose.invertases.vacuolar
major CHO metabolism.degradation.sucrose.hexokinase
major CHO metabolism.degradation.starch
major CHO metabolism.degradation.starch.starch cleavage
major CHO metabolism.degradation.starch.starch cleavage.beta amylase
major CHO metabolism.degradation.starch.starch phosphorylase

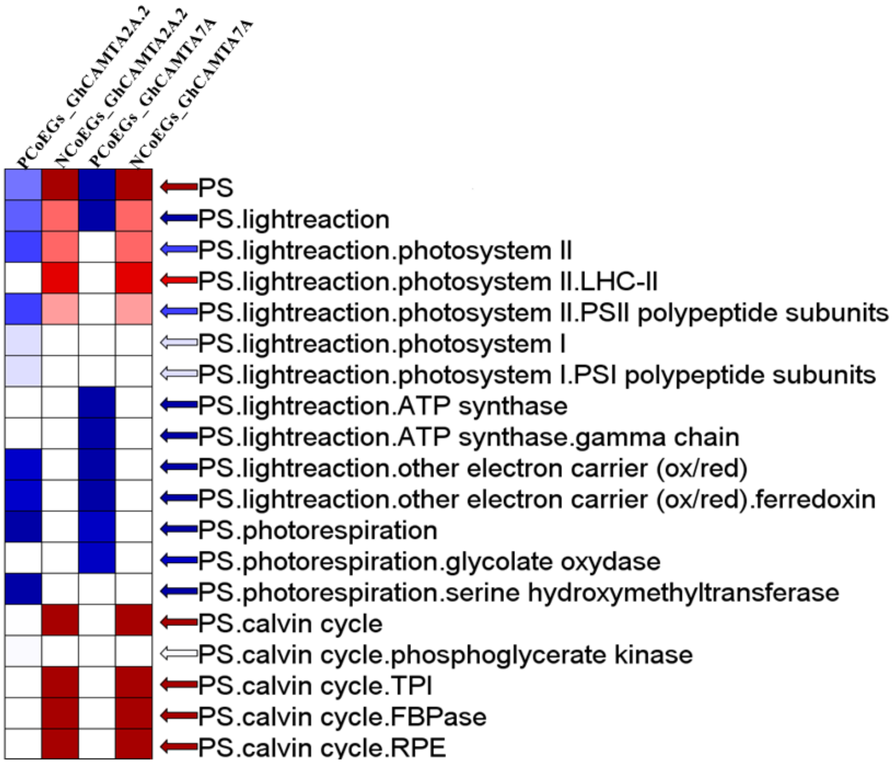
PCoEGs_GhCAMTA2A.2
NCoEGs_GhCAMTA2A.2
PCoEGs_GhCAMTA7A
NCoEGs_GhCAMTA7A
PS
PS.lightreaction
PS.lightreaction.photosystem II
PS.lightreaction.photosystem II.LHC-II
PS.lightreaction.photosystem II.PSII polypeptide subunits
PS.lightreaction.photosystem I
PS.lightreaction.photosystem I.PSI polypeptide subunits
PS.lightreaction.ATP synthase
PS.lightreaction.ATP synthase.gamma chain
PS.lightreaction.other electron carrier (ox/red)
PS.lightreaction.other electron carrier (ox/red).ferredoxin
PS.photorespiration
PS.photorespiration.glycolate oxydase
PS.photorespiration.serine hydroxymethyltransferase
PS.calvin cycle
PS.calvin cycle.phosphoglycerate kinase
PS.calvin cycle.TPI
PS.calvin cycle.FBPase
PS.calvin cycle.RPE

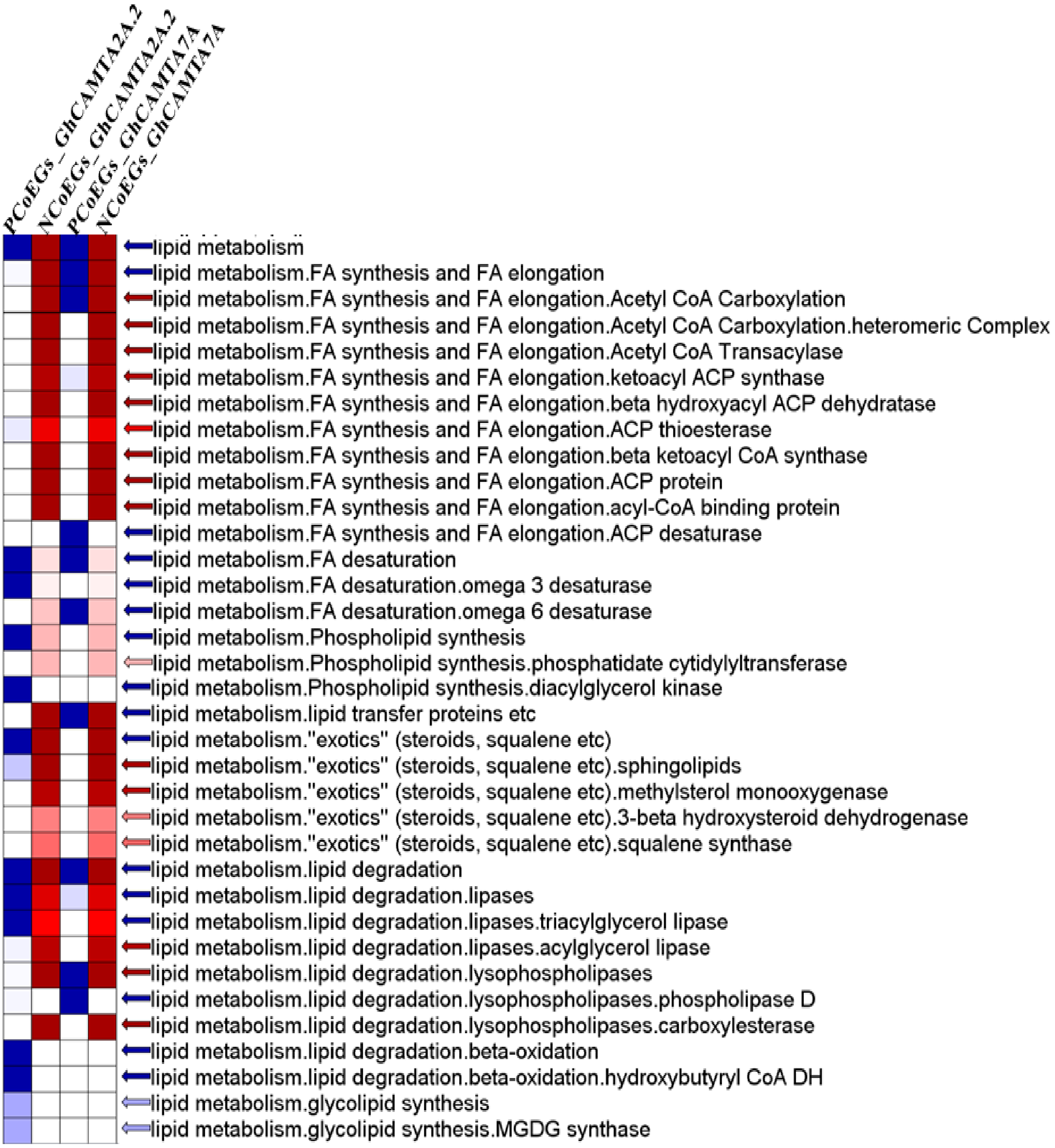

PCoEGs_ GhCAMTA2A.2
NCoEGs_ GhCAMTA2A.2
PCoEGs_ GhCAMTA7A
NCoEGs_ GhCAMTA7A
lipid metabolism
lipid metabolism.FA synthesis and FA elongation
lipid metabolism.FA synthesis and FA elongation.Acetyl CoA Carboxylation
lipid metabolism.FA synthesis and FA elongation.Acetyl CoA Carboxylation.heteromeric Complex
lipid metabolism.FA synthesis and FA elongation.Acetyl CoA Transacylase
lipid metabolism.FA synthesis and FA elongation.ketoacyl ACP synthase
lipid metabolism.FA synthesis and FA elongation.beta hydroxyacyl ACP dehydratase
lipid metabolism.FA synthesis and FA elongation.ACP thioesterase
lipid metabolism.FA synthesis and FA elongation.beta ketoacyl CoA synthase
lipid metabolism.FA synthesis and FA elongation.ACP protein
lipid metabolism.FA synthesis and FA elongation.acyl-CoA binding protein
lipid metabolism.FA synthesis and FA elongation.ACP desaturase
lipid metabolism.FA desaturation
lipid metabolism.FA desaturation.omega 3 desaturase
lipid metabolism.FA desaturation.omega 6 desaturase
lipid metabolism.Phospholipid synthesis
lipid metabolism.Phospholipid synthesis.phosphatidate cytidylyltransferase
lipid metabolism.Phospholipid synthesis.diacylglycerol kinase
lipid metabolism.lipid transfer proteins etc
lipid metabolism."exotics" (steroids, squalene etc)
lipid metabolism."exotics" (steroids, squalene etc).sphingolipids
lipid metabolism."exotics" (steroids, squalene etc).methylsterol monooxygenase
lipid metabolism."exotics" (steroids, squalene etc).3-beta hydroxysteroid dehydrogenase
lipid metabolism."exotics" (steroids, squalene etc).squalene synthase
lipid metabolism.lipid degradation
lipid metabolism.lipid degradation.lipases
lipid metabolism.lipid degradation.lipases.triacylglycerol lipase
lipid metabolism.lipid degradation.lipases.acylglycerol lipase
lipid metabolism.lipid degradation.lysophospholipases
lipid metabolism.lipid degradation.lysophospholipases.phospholipase D
lipid metabolism.lipid degradation.lysophospholipases.carboxylesterase
lipid metabolism.lipid degradation.beta-oxidation
lipid metabolism.lipid degradation.beta-oxidation.hydroxybutyryl CoA DH
lipid metabolism.glycolipid synthesis
lipid metabolism.glycolipid synthesis.MGDG synthase

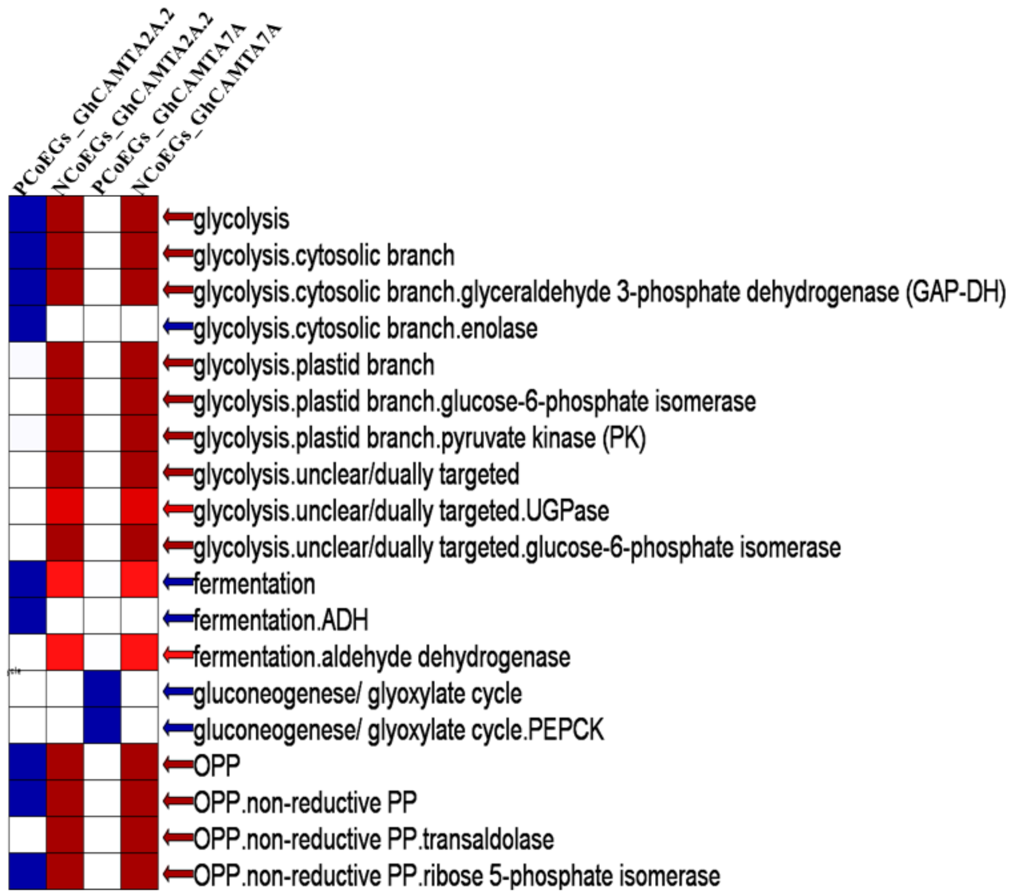
PCoEGs _GhCAMTA2A.2
NCoEGs _GhCAMTA2A.2
PCoEGs _GhCAMTA7A
NCoEGs _GhCAMTA7A
glycolysis
glycolysis.cytosolic branch
glycolysis.cytosolic branch.glyceraldehyde 3-phosphate dehydrogenase (GAP-DH)
glycolysis.cytosolic branch.enolase
glycolysis.plastid branch
glycolysis.plastid branch.glucose-6-phosphate isomerase
glycolysis.plastid branch.pyruvate kinase (PK)
glycolysis.unclear/dually targeted
glycolysis.unclear/dually targeted.UGPase
glycolysis.unclear/dually targeted.glucose-6-phosphate isomerase
fermentation
fermentation.ADH
fermentation.aldehyde dehydrogenase
gluconeogenese/ glyoxylate cycle
gluconeogenese/ glyoxylate cycle.PEPCK
OPP
OPP.non-reductive PP
OPP.non-reductive PP.transaldolase
OPP.non-reductive PP.ribose 5-phosphate isomerase

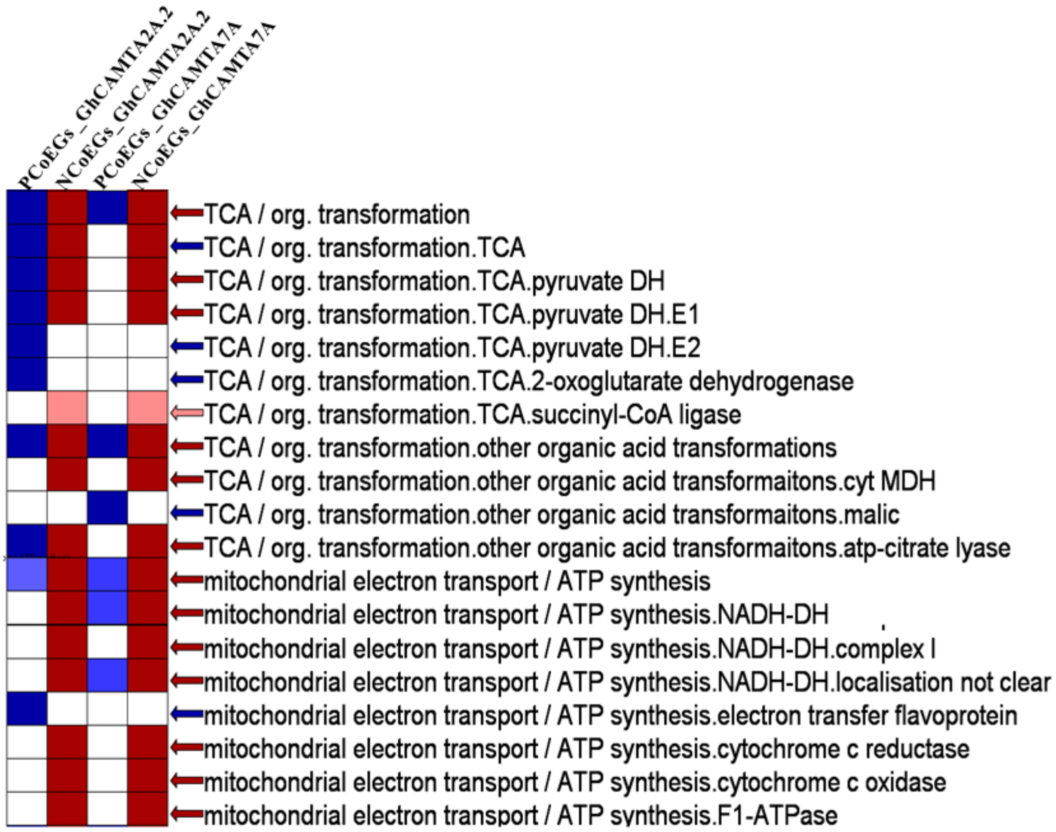
PCoEGs _GhCAMTA2A.2
NCoEGs _GhCAMTA2A.2
PCoEGs _GhCAMTA7A
NCoEGs _GhCAMTA7A
TCA / org. transformation
TCA / org. transformation.TCA
TCA / org. transformation.TCA.pyruvate DH
TCA / org. transformation.TCA.pyruvate DH.E1
TCA / org. transformation.TCA.pyruvate DH.E2
TCA / org. transformation.TCA.2-oxoglutarate dehydrogenase
TCA / org. transformation.TCA.succinyl-CoA ligase
TCA / org. transformation.other organic acid transformations
TCA / org. transformation.other organic acid transformaitons.cyt MDH
TCA / org. transformation.other organic acid transformaitons.malic
TCA / org. transformation.other organic acid transformaitons.atp-citrate lyase
mitochondrial electron transport / ATP synthesis
mitochondrial electron transport / ATP synthesis.NADH-DH
mitochondrial electron transport / ATP synthesis.NADH-DH.complex I
mitochondrial electron transport / ATP synthesis.NADH-DH.localisation not clear
mitochondrial electron transport / ATP synthesis.electron transfer flavoprotein
mitochondrial electron transport / ATP synthesis.cytochrome c reductase
mitochondrial electron transport / ATP synthesis.cytochrome c oxidase
mitochondrial electron transport / ATP synthesis.F1-ATPase

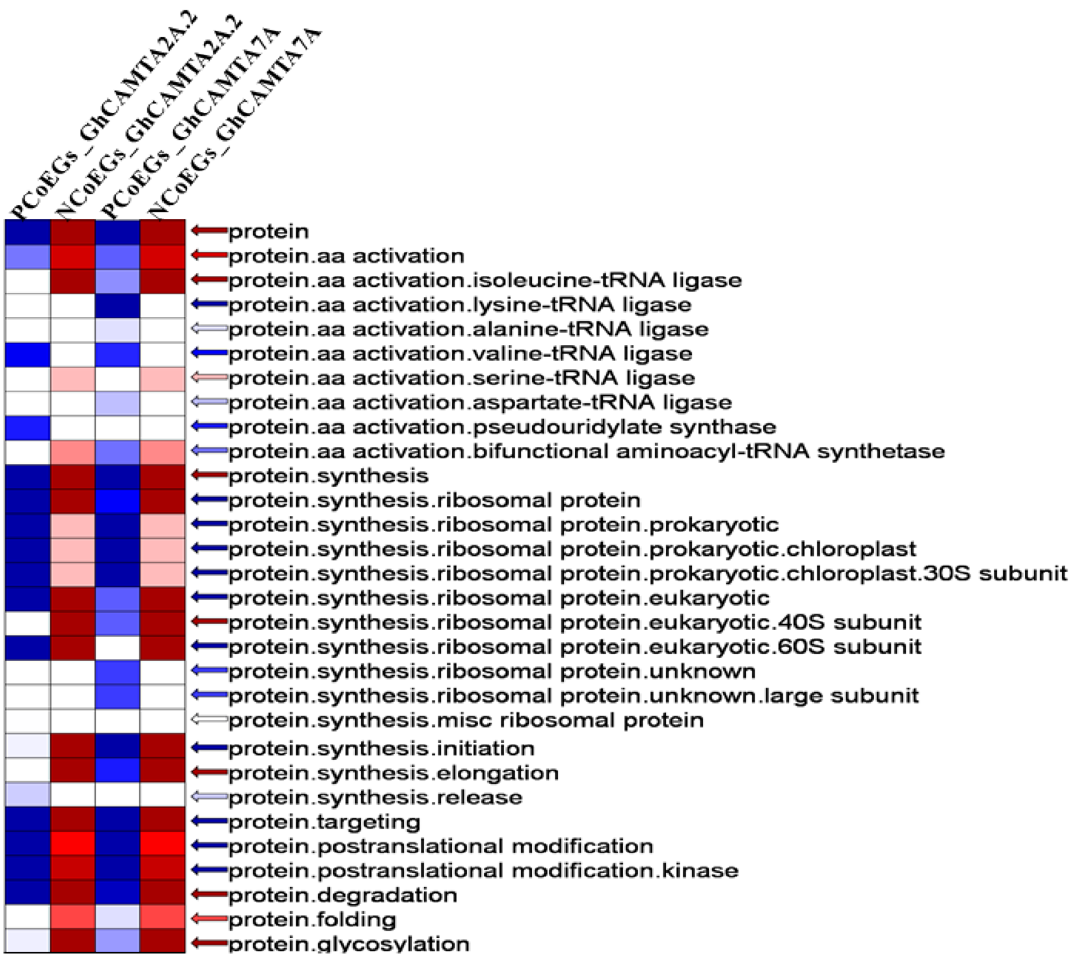

PCoEGs _GhCAMTA2A.2
NCoEGs_GhCAMTA2A.2
PCoEGs _GhCAMTA7A
NCoEGs_GhCAMTA7A
protein
protein.aa activation
protein.aa activation.isoleucine-tRNA ligase
protein.aa activation.lysine-tRNA ligase
protein.aa activation.alanine-tRNA ligase
protein.aa activation.valine-tRNA ligase
protein.aa activation.serine-tRNA ligase
protein.aa activation.aspartate-tRNA ligase
protein.aa activation.pseudouridylate synthase
protein.aa activation.bifunctional aminoacyl-tRNA synthetase
protein.synthesis
protein.synthesis.ribosomal protein
protein.synthesis.ribosomal protein.prokaryotic
protein.synthesis.ribosomal protein.prokaryotic.chloroplast
protein.synthesis.ribosomal protein.prokaryotic.chloroplast.30S subunit
protein.synthesis.ribosomal protein.eukaryotic
protein.synthesis.ribosomal protein.eukaryotic.40S subunit
protein.synthesis.ribosomal protein.eukaryotic.60S subunit
protein.synthesis.ribosomal protein.unknown
protein.synthesis.ribosomal protein.unknown.large subunit
protein.synthesis.misc ribosomal protein
protein.synthesis.initiation
protein.synthesis.elongation
protein.synthesis.release
protein.targeting
protein.postranslational modification
protein.postranslational modification.kinase
protein.degradation
protein.folding
protein.glycosylation


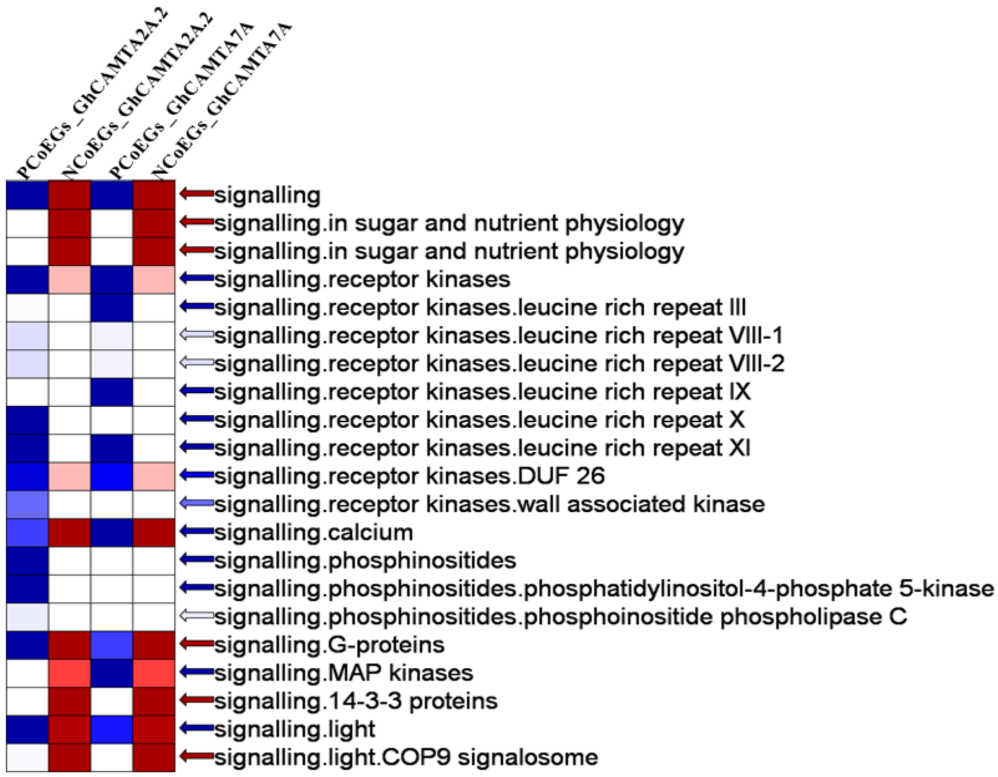

PCoEGs _GhCAMTA2A.2
NCoEGs_GhCAMTA2A.2
PCoEGs _GhCAMTA7A
NCoEGs_GhCAMTA7A
signalling
signalling.in sugar and nutrient physiology
signalling.in sugar and nutrient physiology
signalling.receptor kinases
signalling.receptor kinases.leucine rich repeat III
signalling.receptor kinases.leucine rich repeat VIII-1
signalling.receptor kinases.leucine rich repeat VIII-2
signalling.receptor kinases.leucine rich repeat IX
signalling.receptor kinases.leucine rich repeat X
signalling.receptor kinases.leucine rich repeat XI
signalling.receptor kinases.DUF 26
signalling.receptor kinases.wall associated kinase
signalling.calcium
signalling.phosphinositides
signalling.phosphinositides.phosphatidylinositol-4-phosphate 5-kinase
signalling.phosphinositides.phosphoinositide phospholipase C
signalling.G-proteins
signalling.MAP kinases
signalling.14-3-3 proteins
signalling.light
signalling.light.COP9 signalosome

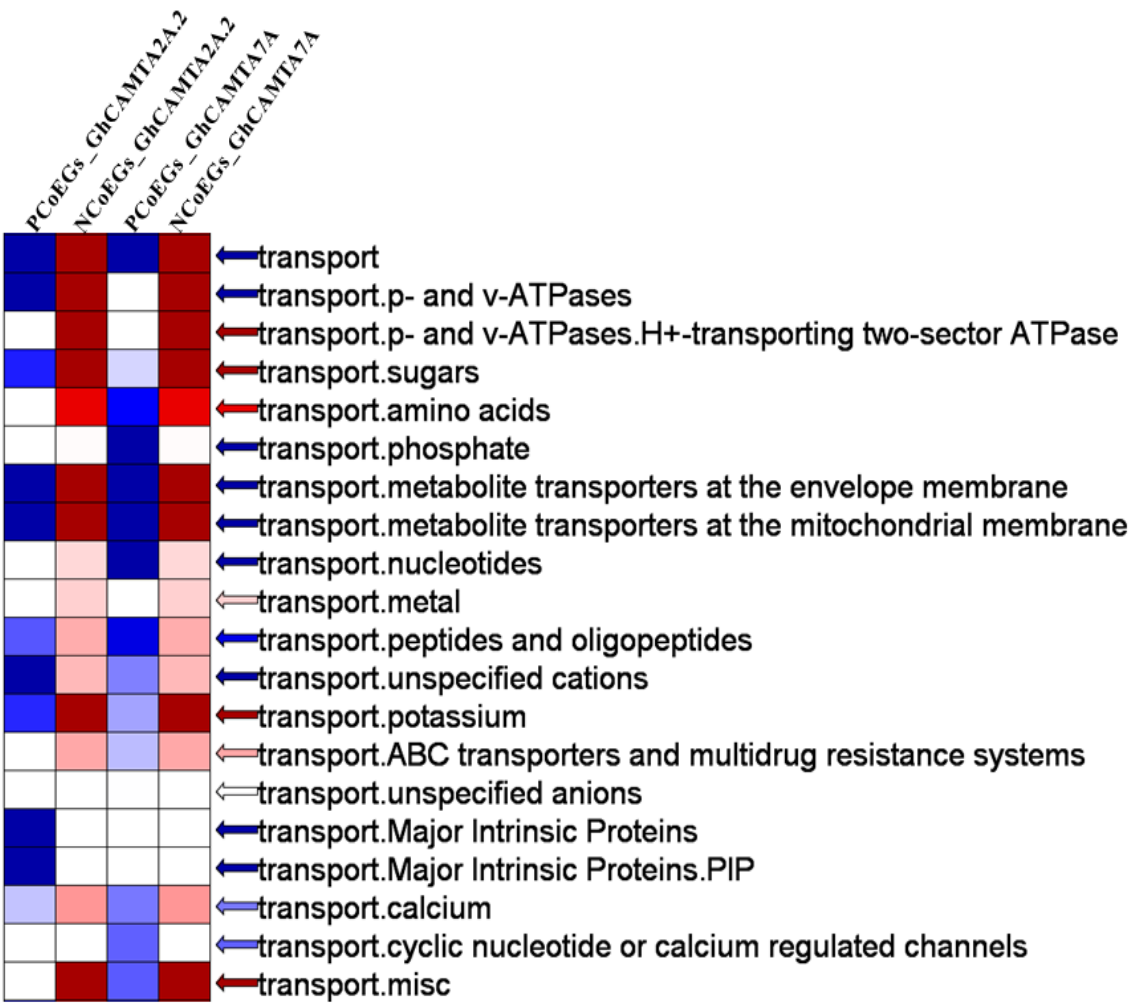


**Supplementary Fig. S6 MapMan analysis of the positively and negatively co-expressed genes with GhCAMTA2A.2 and GhCAMTA7A.** The significant functional categories of PCoEGs and NCoEGs with GhCAMTA2A.2 and GhCAMTA7A were determined by MapMan software. Expression values ($Log_2$ FPKM) of PCoEGs and NCoEGs during different cotton fiber developmental stages were taken as input in MapMan analysis. BINs coloured in green and red are significantly positively and negatively regulated groups respectively.

**Supplementary Table S1** List of Plants used for Phylogenetic analysis

| | Family | Plant | | |
|---|---|---|---|---|
| | | **Botanical Name** | **Common Name** | **Genome Size** |
| **Bryophyte** | Funariaceae | *Physcomitrella patens* | Spreading earth moss | ~500 Mbp |
| **Lycopodiophyta** | Selaginellaceae | *Selaginella moellendorffii* | | ~100 Mbp |
| **Monocots** | Musaceae | *Musa acuminata* | Banana | ~523 Mbp |
| | Poaceae | *Oryza sativa* | Rice | ~500 Mbp |
| | | *Zea mays* | Maize | ~2.4Gbp |
| | | *Setaria italica* | Foxtail millet | ~490Mbp |
| | | *Sorghum bicolor* | Great millet | ~730 Mb |
| | | *Triticum aestivum* | Bread wheat | ~17 Gbp |
| | | *Brachypodium distachyon* | Purple false brome/stiff brome | ~270 Mbp |
| **Eudicots** | Brassicaceae | *Arabidopsis thaliana* | Mouse-ear cress or arabidopsis | ~135 Mbp |
| | Malvaceae | *Gossypium arboreum* | Cotton | ~1,746 Mbp |
| | | *Gossypium raimondii* | Cotton | ~880Mbp |
| | | *Gossypium hirsutum* | Cotton | ~2400 Mbp |
| | | *Theobroma cacao* | Cacao or chocolate tree | ~445 Mbp |
| | Salicaceae | *Poplar tricocharpa* | Populus | ~500Mbp |
| | Vitaceae | *Vitis vinifera* | Grape | ~500Mbp |
| | Euphorbiaceae | *Ricinus communis* | Castor bean | ~320 Mbp |
| | Fabaceae | *Glycine max* | Soybean | ~1,115 Mbp |
| | Caricaceae | *Carica papaya* | Papaya | ~372 Mbp |
| **Gymnosperm** | Pinaceae | *Pinus taeda* | Loblolly pine | ~20.1 Gbp |

**Supplementary Table S2** The molecular evolutionary rates (Ka, Ks, Ka/Ks ratios) of orthologs CAMTA gene pairs among *Gossypium* taxa.

| | | A Vs D | | | |
|---|---|---|---|---|---|
| **A** | **D** | **Ks** | **Ka** | **Ka/Ks** | **Selective pressure** |
| GaCAMTA2.2 | GrCAMTA2.2 | 0.0322 | 0.0117 | 0.3632 | Purifying selection |
| GaCAMTA5.1 | GrCAMTA5.2 | 0.0327 | 0.0184 | 0.5618 | Purifying selection |
| GaCAMTA2.1 | GrCAMTA2.1 | 0.0404 | 0.0127 | 0.3143 | Purifying selection |
| GaCAMTA5.2 | GrCAMTA5.3 | 0.0458 | 0.0143 | 0.3121 | Purifying selection |
| GaCAMTA7 | GrCAMTA7 | 0.051 | 0.0312 | 0.6123 | Purifying selection |
| | | **$A_T$ Vs $D_T$** | | | |
| **$A_T$** | **$D_T$** | **Ks** | **Ka** | **Ka/K** | **Selective pressure** |
| GhCAMTA7A | GhCAMTA7D | 0.0478 | 0.0267 | 0.5594 | Purifying selection |
| GhCAMTA2A.2 | GhCAMTA2D.1 | 0.0319 | 0.0116 | 0.364 | Purifying selection |
| GhCAMTA3A.1 | GhCAMTA3D.1 | 0.0324 | 0.0187 | 0.5757 | Purifying selection |
| | | **$D_T$ Vs D** | | | |
| **$D_T$** | **D** | **Ks** | **Ka** | **Ka/Ks** | **Selective pressure** |
| GhCAMTA5D.1 | GrCAMTA5.3 | 0.0178 | 0.0049 | 0.2752 | Purifying selection |
| GhCAMTA2D.1 | GrCAMTA2.2 | 0.0159 | 0.0034 | 0.2163 | Purifying selection |
| GhCAMTA3D.1 | GrCAMTA3.1 | 0.0277 | 0.019 | 0.687 | Purifying selection |
| GhCAMTA7D | GrCAMTA7 | 0.0153 | 0.0064 | 0.4173 | Purifying selection |
| | | **$A_T$ Vs A** | | | |
| **$A_T$** | **A** | **Ks** | **Ka** | **Ka/Ks** | **Selective pressure** |
| GhCAMTA2A.1 | GaCAMTA2.1 | 0.0044 | 0.0008 | 0.1851 | Purifying selection |
| GhCAMTA7A | GaCAMTA7 | 0.0064 | 0.0056 | 0.8751 | Purifying selection |

**Supplementary Table S3** List of Primers used for qRT-PCR.

| Primer Name | Sequence 5' to 3' |
|---|---|
| GhCAMTA7A_F | GTTTACTAGTGGACCCAGGATCACA |
| GhCAMTA7A _R | TGGGAGATACCTGAATAGTAGCAGCA |
| GhCAMTA2A.2 _F | GAGAGTCCAGTGATGCTGTGTCTGAT |
| GhCAMTA2A.2 _R | CTTTGAAATGGCCTGCGTGTCT |
| GhCAMTA3D.1 _F | CCTCGGCTCCAGTTCTCCC |
| GhCAMTA3D.1 _R | TGTCCTCCTCCAGCAATGAATTAATT |
